# Supplementary material for: eUTOPIA: solUTion for Omics data PreprocessIng and Analysis
Source: Source Code Biol Med. 2019 Jan 29;14:1. doi: 10.1186/s13029-019-0071-7 (PMC6352382; doi:10.1186/s13029-019-0071-7)
Supplement: Supplementary file 1 — eUTOPIA user manual with sample analysis. (DOCX 15056 kb) [file 13029_2019_71_MOESM1_ESM.docx]

eUTOPIA User Manual

*with Sample Data Analysis*

Contents

[About eUTOPIA 3](#_Toc511642542)

[Sample Data 4](#_Toc511642543)

[Setup 5](#_Toc511642544)

[Launch 6](#_Toc511642545)

[Initialize 7](#_Toc511642546)

[Workflow Interface 8](#_Toc511642547)

[Phenotype Specification 9](#_Toc511642548)

[Remove Samples 14](#_Toc511642549)

[Raw Data 14](#_Toc511642550)

[Quality Control 15](#_Toc511642551)

[Filter Probes 15](#_Toc511642552)

[Normalization 16](#_Toc511642553)

[Normalization Plots 17](#_Toc511642554)

[Technical Variation 19](#_Toc511642555)

[Batch Correction 23](#_Toc511642556)

[Known Batch Correction 23](#_Toc511642557)

[Technical Variation After Known Correction 26](#_Toc511642558)

[Unknown Batch Correction 27](#_Toc511642559)

[Technical Variation After Unknown Correction 30](#_Toc511642560)

[Annotation 30](#_Toc511642561)

[Differential Analysis 33](#_Toc511642562)

[Differential Results 35](#_Toc511642563)

[Intersection Plot 37](#_Toc511642564)

[Volcano Plot 39](#_Toc511642565)

[Visualize Expression/Methylation 40](#_Toc511642566)

[Reporting 47](#_Toc511642567)

[Terminate eUTOPIA Session 49](#_Toc511642568)

[eUTOPIA dependencies 50](#_Toc511642569)

[References 51](#_Toc511642570)

# About eUTOPIA

eUTOPIA is designed to perform preprocessing and analysis of microarray data from different microarray platforms.

1. Agilent 2-color
2. Agilent 1-color
3. Affymetrix expression
4. Illumina methylation (450k, EPIC)

eUTOPIA processes the microarray raw data through a guided workflow with defined steps that are executed by the user from the graphical interface. The workflow is designed to be intuitive and enables the user to make decisions at important steps to best suit their analytical goals.

Preprocessing involves quality control reporting of raw data, filtering poor quality probes, normalization of raw data to account for expression distributions from different arrays. Most importantly user can perform correction of technical variability not represented by the biological variables. Correction of data can be performed for known technical variables, while surrogate variables not known beforehand can be identified for correction. Data correction must be performed with care and eUTOPIA’s workflow allows the user to understand the representation of the known and surrogate variables by representing the technical variation graphically in plots. Annotation matching the raw microarray data must be provided by the user alternatively eUTOPIA can use the annotation from the raw data to aggregate the probes to genomic annotation features. The user can choose to export preprocessed data at different levels of processing.

Differential analysis of preprocessed data is performed by defining the model to use in limma analysis with the specification of the *variable of interest* and additive *covariates*, in addition to this the user defines the comparisons for differential analysis from the variable of interest. Results from differential analysis can be filtered by specifying the logFC and p.value thresholds. These results are supplemented with different graphical representations to see; the intersection of features identified in different comparisons, representation of features by logFC and p.value as volcano plot, representation of differential features as heatmap to see the grouping of samples. These results can be exported by the user and the graphical representation can be exported as a PDF report of plots generated at different levels of preprocessing and analysis.

# Sample Data

## Sample Expression Data

<https://www.ncbi.nlm.nih.gov/geo/query/acc.cgi?acc=GSE92900>.

Distinct sets of genes representing overlapping biological functions are altered by intrinsic properties of carbon nanomaterials in vitro and in vivo [mouse], GEO (Barrett *et al.*, 2013) accession GSE92900 (Kinaret *et al.*, 2017). [Raw data download link](ftp://ftp.ncbi.nlm.nih.gov/geo/series/GSE92nnn/GSE92900/suppl/GSE92900_raw_data_files.tar.gz).

## Phenotype Information

| **SampleID** | **group** | **dye** | **slide** | **area** | **array** | **RIN** | **Qubit_conc** | **dye_conc** | **dye_activity** | **n.mice** | **operator** | **date** | **file** |
| --- | --- | --- | --- | --- | --- | --- | --- | --- | --- | --- | --- | --- | --- |
| full_23 | Fullerene | cy3 | s252800520993 | 1_1 | 252800520993_1_1 | 8.6 | 422.8 | 325.25 | 18.263 | 2 | natm | 17.9.2014 | US11263921_252800520993_S01_GE2_1105_Oct12_1_1.txt |
| gnf_16 | GNF | cy3 | s252800520993 | 1_2 | 252800520993_1_2 | 8.5 | 286.32 | 349.14 | 17.815 | 2 | natm | 17.9.2014 | US11263921_252800520993_S01_GE2_1105_Oct12_1_2.txt |
| bayt_17 | Baytubes | cy3 | s252800520993 | 1_3 | 252800520993_1_3 | 8.7 | 512.61 | 226.49 | 15.453 | 2 | natm | 17.9.2014 | US11263921_252800520993_S01_GE2_1105_Oct12_1_3.txt |
| tcnt_8 | tCNT | cy3 | s252800520993 | 2_1 | 252800520993_2_1 | 9.1 | 335.01 | 299.57 | 0 | 2 | natm | 17.9.2014 | US11263921_252800520993_S01_GE2_1105_Oct12_2_1.txt |
| ses_12 | SES | cy3 | s252800520993 | 2_3 | 252800520993_2_3 | 8.6 | 315.98 | 303.43 | 0.165 | 3 | natm | 17.9.2014 | US11263921_252800520993_S01_GE2_1105_Oct12_2_3.txt |
| bayt_18 | Baytubes | cy3 | s252800520993 | 2_4 | 252800520993_2_4 | 8.7 | 212.93 | 308.33 | 14.757 | 2 | natm | 17.9.2014 | US11263921_252800520993_S01_GE2_1105_Oct12_2_4.txt |
| ctrl_3 | Ctrl | cy3 | s252800520994 | 1_1 | 252800520994_1_1 | 8.9 | 124.26 | 340.16 | 18.932 | 2 | natm | 22.9.2014 | US11263921_252800520994_S01_GE2_1105_Oct12_1_1.txt |
| ses_10 | SES | cy3 | s252800520994 | 1_2 | 252800520994_1_2 | 8.7 | 298.68 | 299.16 | 24.97 | 2 | natm | 22.9.2014 | US11263921_252800520994_S01_GE2_1105_Oct12_1_2.txt |
| gnf_14 | GNF | cy3 | s252800520994 | 1_4 | 252800520994_1_4 | 8.5 | 280.89 | 304.96 | 18.002 | 2 | natm | 22.9.2014 | US11263921_252800520994_S01_GE2_1105_Oct12_1_4.txt |
| ctrl_1 | Ctrl | cy3 | s252800520994 | 2_1 | 252800520994_2_1 | 9 | 220.83 | 193.02 | 18.962 | 2 | natm | 22.9.2014 | US11263921_252800520994_S01_GE2_1105_Oct12_2_1.txt |
| rcnt_5 | rCNT | cy3 | s252800520995 | 1_1 | 252800520995_1_1 | 9.1 | 467.62 | 197.3 | 25.291 | 2 | tsui | 6.10.2014 | US11263921_252800520995_S01_GE2_1105_Oct12_1_1.txt |
| full_24 | Fullerene | cy3 | s252800520995 | 2_3 | 252800520995_2_3 | 8.5 | 289.37 | 339.05 | 23.448 | 2 | tsui | 6.10.2014 | US11263921_252800520995_S01_GE2_1105_Oct12_2_3.txt |
| gnf_15 | GNF | cy5 | s252800520993 | 1_1 | 252800520993_1_1 | 8.5 | 278.86 | 334.62 | 19.814 | 2 | natm | 17.9.2014 | US11263921_252800520993_S01_GE2_1105_Oct12_1_1.txt |
| rcnt_4 | rCNT | cy5 | s252800520993 | 1_2 | 252800520993_1_2 | 8.7 | 253.34 | 162.21 | 14.487 | 3 | natm | 17.9.2014 | US11263921_252800520993_S01_GE2_1105_Oct12_1_2.txt |
| tcnt_7 | tCNT | cy5 | s252800520993 | 1_3 | 252800520993_1_3 | 8.4 | 267.68 | 252.83 | 20.132 | 3 | natm | 17.9.2014 | US11263921_252800520993_S01_GE2_1105_Oct12_1_3.txt |
| ses_11 | SES | cy5 | s252800520993 | 2_1 | 252800520993_2_1 | 8.8 | 243.79 | 330.15 | 2.151 | 2 | natm | 17.9.2014 | US11263921_252800520993_S01_GE2_1105_Oct12_2_1.txt |
| bayt_19 | Baytubes | cy5 | s252800520993 | 2_3 | 252800520993_2_3 | 8.7 | 345.04 | 231.78 | 1.898 | 2 | natm | 17.9.2014 | US11263921_252800520993_S01_GE2_1105_Oct12_2_3.txt |
| ctrl_2 | Ctrl | cy5 | s252800520993 | 2_4 | 252800520993_2_4 | 8.9 | 225.7 | 362.35 | 19.705 | 3 | natm | 17.9.2014 | US11263921_252800520993_S01_GE2_1105_Oct12_2_4.txt |
| bayt_20 | Baytubes | cy5 | s252800520994 | 1_1 | 252800520994_1_1 | 8.7 | 316.62 | 382.83 | 26.853 | 2 | natm | 22.9.2014 | US11263921_252800520994_S01_GE2_1105_Oct12_1_1.txt |
| full_22 | Fullerene | cy5 | s252800520994 | 1_2 | 252800520994_1_2 | 8.5 | 299.59 | 363.73 | 27.108 | 2 | natm | 22.9.2014 | US11263921_252800520994_S01_GE2_1105_Oct12_1_2.txt |
| rcnt_6 | rCNT | cy5 | s252800520994 | 1_4 | 252800520994_1_4 | 9.2 | 554.125 | 326.2 | 23.237 | 2 | natm | 22.9.2014 | US11263921_252800520994_S01_GE2_1105_Oct12_1_4.txt |
| gnf_13 | GNF | cy5 | s252800520994 | 2_1 | 252800520994_2_1 | 8.5 | 256.89 | 3.69 | 37.94 | 2 | natm | 22.9.2014 | US11263921_252800520994_S01_GE2_1105_Oct12_2_1.txt |
| full_21 | Fullerene | cy5 | s252800520995 | 1_1 | 252800520995_1_1 | 8.3 | 365.75 | 271.99 | 21.582 | 2 | tsui | 6.10.2014 | US11263921_252800520995_S01_GE2_1105_Oct12_1_1.txt |
| tcnt_9 | tCNT | cy5 | s252800520995 | 2_3 | 252800520995_2_3 | 9 | 347.57 | 356.21 | 21.346 | 2 | tsui | 6.10.2014 | US11263921_252800520995_S01_GE2_1105_Oct12_2_3.txt |

Phenotype Table Description

| **Column Name** | **Column Description** |
| --- | --- |
| SampleID | Unique identifier for the samples |
| group | Grouping variable for samples by nanomaterial exposure |
| dye | Microarray dye information. Cy5 for red channel and Cy3 for the green channel |
| slide | Identifier for the microarray slide |
| area | Identifier for the area in the slide |
| array | Identifier for the array from a specific slide |
| RIN | RNA Integrity Number as a quantitative measure of RNA quality from Agilent BioAnalyzer |
| Qubit_conc | RNA integrity measure from Qubit assay |
| dye_conc | Dye concentration reported by NanoDrop quantification |
| dye_activity | Specific activity determined from the NanoDrop quantification |
| n.mice | Number of mouse samples |
| operator | Code of the person responsible for the microarray experiment |
| date | Microarray experiment date |
| File | Filename for the microarray raw data file (base filename without directory path) |

Color Code

| Array Information | Information about microarray |
| --- | --- |
| Sample Information. | Information about samples associated with microarrays |
| RNA quality & Sample Preparation | Experimental quality estimation of RNA extracted from samples |
| Technical Information | Technical information associated with samples |
| Experiment Information | Information associated with microarray experimentation |

# Setup

## Install R Dependencies

#Install impute dependency

source("http://bioconductor.org/biocLite.R")

biocLite("impute")

#Install CRAN dependencies

cran_pkgs <- c("swamp", "infotheo", "gplots", "RColorBrewer", "shiny", "shinyjs", "shinyBS", "shinydashboard", "shinyFiles",

"DT", "shinycssloaders", "ggplot2", "ggrepel", "WriteXLS", "rmarkdown", "VennDiagram", "grid", "futile.logger", "reshape2",

"htmlTable", "devtools", "httr", "randomcoloR")

cran_pkgs.inst <- cran_pkgs[!(cran_pkgs %in% rownames(installed.packages()))]

if(length(cran_pkgs.inst)>0){

print(paste0("Missing ", length(cran_pkgs.inst), " CRAN Packages:"))

for(pkg in cran_pkgs.inst){

print(paste0("Installing Package:'", pkg, "'..."))

install.packages(pkg, repo="http://cran.rstudio.org", dependencies=TRUE)

print("Installed!!!")

}

}

#Install latest version of rhandsontable from GitHub

print("Installing rhandsontable from GitHub!")

devtools::install_github("jrowen/rhandsontable")

#Install latest version of UpSetR from GitHub

print("Installing UpSetR from GitHub!")

devtools::install_github("hms-dbmi/UpSetR")

#Install Bioconductor dependencies

source("http://bioconductor.org/biocLite.R")

bioc_pkgs <- c("limma", "sva", "Biobase", "biomaRt", "affy", "affyQCReport", "arrayQualityMetrics", "made4", "vsn", "GEOquery", "minfi",

"IlluminaHumanMethylation450kmanifest", "IlluminaHumanMethylation450kanno.ilmn12.hg19", "IlluminaHumanMethylationEPICmanifest",

"IlluminaHumanMethylationEPICanno.ilm10b2.hg19", "affyio", "simpleaffy", "yaqcaffy", "GO.db", "shinyMethyl")

bioc_pkgs.inst <- bioc_pkgs[!(bioc_pkgs %in% rownames(installed.packages()))]

if(length(bioc_pkgs.inst)>0){

source("http://bioconductor.org/biocLite.R")

print(paste0("Missing ", length(bioc_pkgs.inst), " Bioconductor Packages:"))

for(pkg in bioc_pkgs.inst){

print(paste0("Installing Package:'", pkg, "'..."))

biocLite(pkg, suppressUpdates=TRUE)

print("Installed!!!")

}

}

#Install latest version of GOSemSim from GitHub

print("Installing GOSemSim from GitHub!")

devtools::install_github("GuangchuangYu/GOSemSim")

# Launch

## Run eUTOPIA From GitHub

# Load 'shiny' library

library(shiny)

# Using runGitHub

runGitHub("eUTOPIA", "Greco-Lab", subdir="eUTOPIA-app")

# Using the archived file

runUrl("https://github.com/Greco-Lab/eUTOPIA/archive/master.tar.gz", subdir="eUTOPIA-app")

runUrl("https://github.com/Greco-Lab/eUTOPIA/archive/master.zip", subdir="eUTOPIA-app")

## Download eUTOPIA and Run Locally

# Clone the git repository

git clone https://github.com/Greco-Lab/eUTOPIA eUTOPIA_clone

# Start R session and run by using runApp()

setwd("./eUTOPIA_clone")

library(shiny)

runApp("eUTOPIA-app/")

## Launch eUTOPIA from R console

An instance of eUTOPIA can be started locally by 1. Starting the R console, 2. Attaching the R library shiny *library(shiny)*, and 3. Submitting command *runApp(“eUTOPIA-app”)*, where ‘eUTOPIA-app’ is the path of the directory *eUTOPIA-app* that contains the required R shiny script.


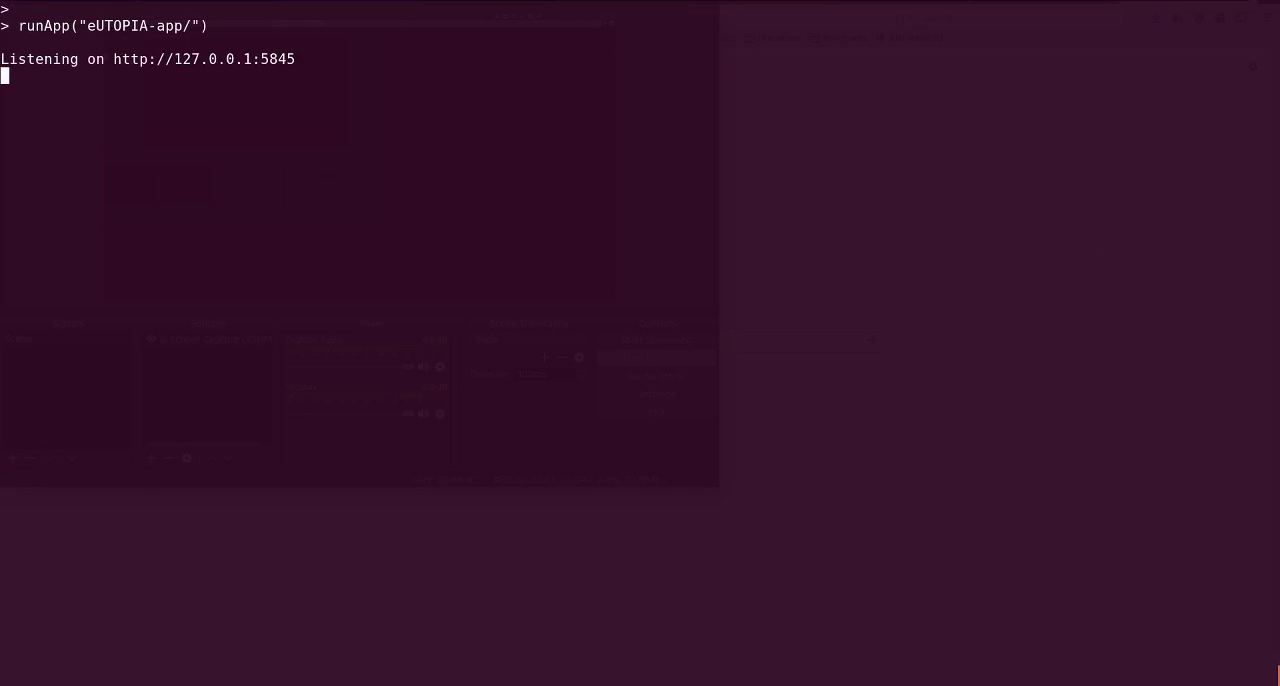


# Initialize

## Welcome Screen


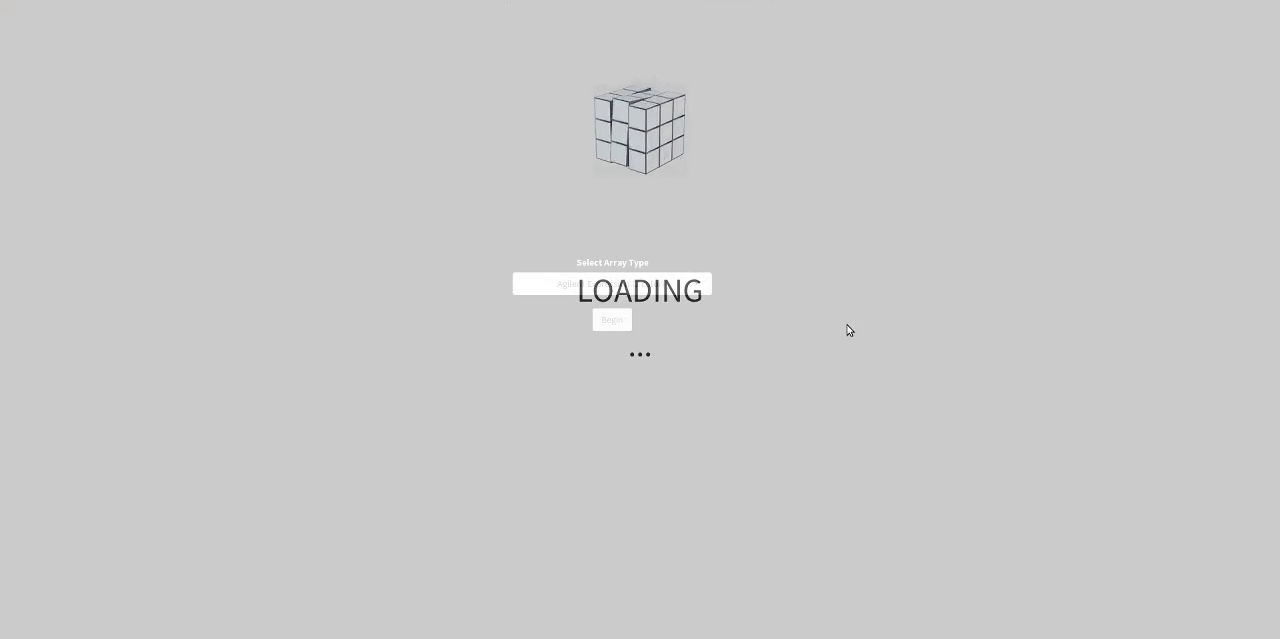


## Platform Selection

INfORM supports Agilent two color, Agilent one color, Affymetrix Expression, and Illumina Methylation platforms for pre-processing and preliminary analysis.


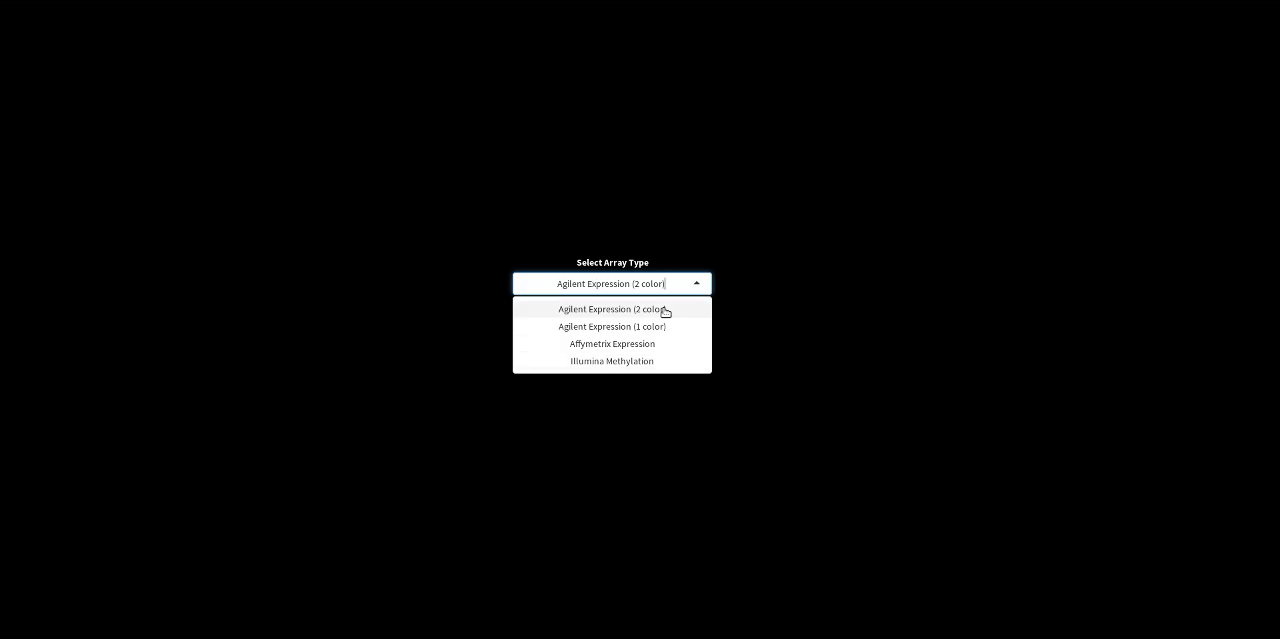


## Begin Analysis


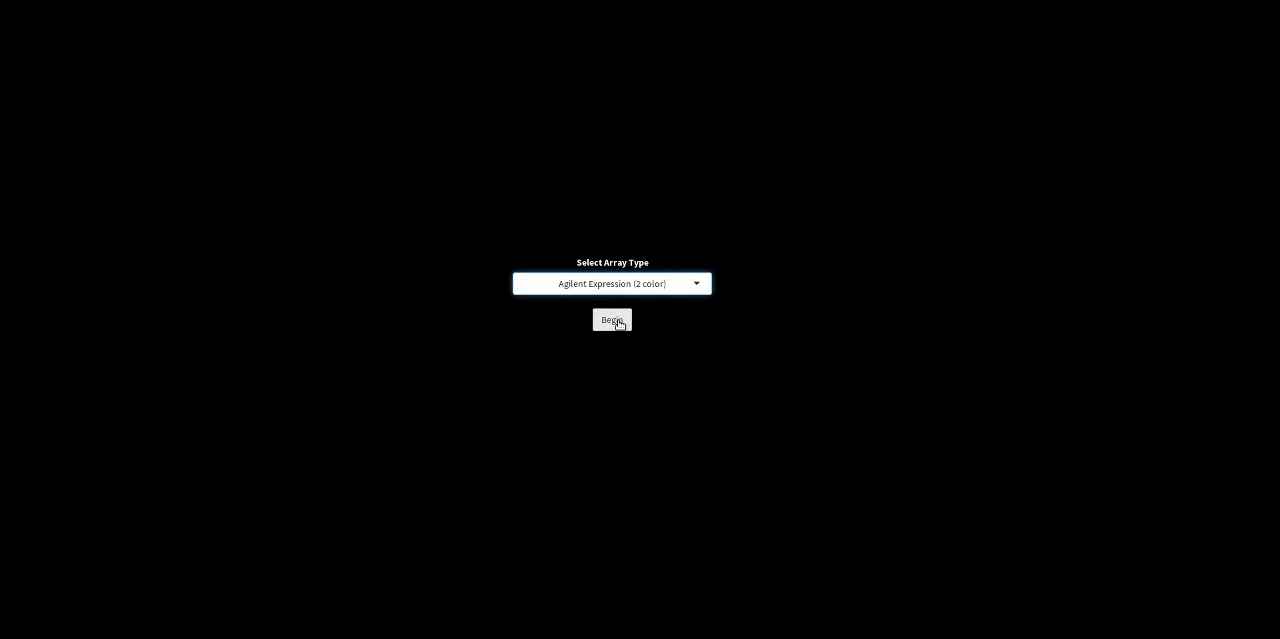


# Workflow Interface

The workflow interface layout has a sidebar with input controls to configure and execute various steps (marked with green outline) and the output of the steps are visualized from the main display area (marked with red outline). The input controls in the sidebar are altered according to the chosen platform. This example analysis is for Agilent two-color platform data.


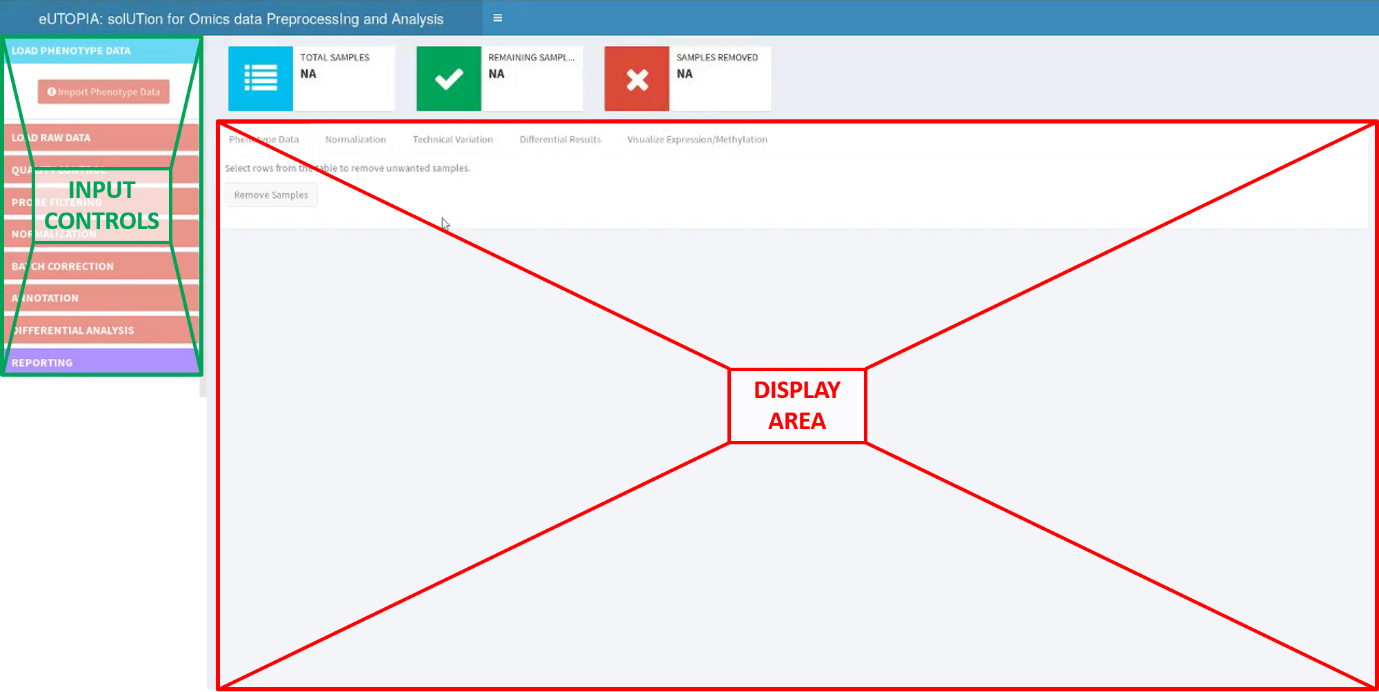


# Phenotype Specification

The phenotype file is a table that contains information about the arrays used in the experimentation and the phenotype information of the samples associated with those arrays. The array information will vary according to the chose microarray platform, it is compulsory that phenotype file contains the array raw data file names (without directory structure), unique identifiers for the samples, and the dye information for the two-color experiment data. For Affymetrix expression, Agilent 1-color, and Agilent 2-color platforms the raw data file names must contain name of the file with extension (.txt or .CEL), while for Illumina methylation (450k and EPIC) platform file basename without the extension must be provided as there are two separate dye specific files for each basename that are identified internally by the pipeline.

## Load Phenotype

Launch a popup window containing controls to configure the phenotype file import.


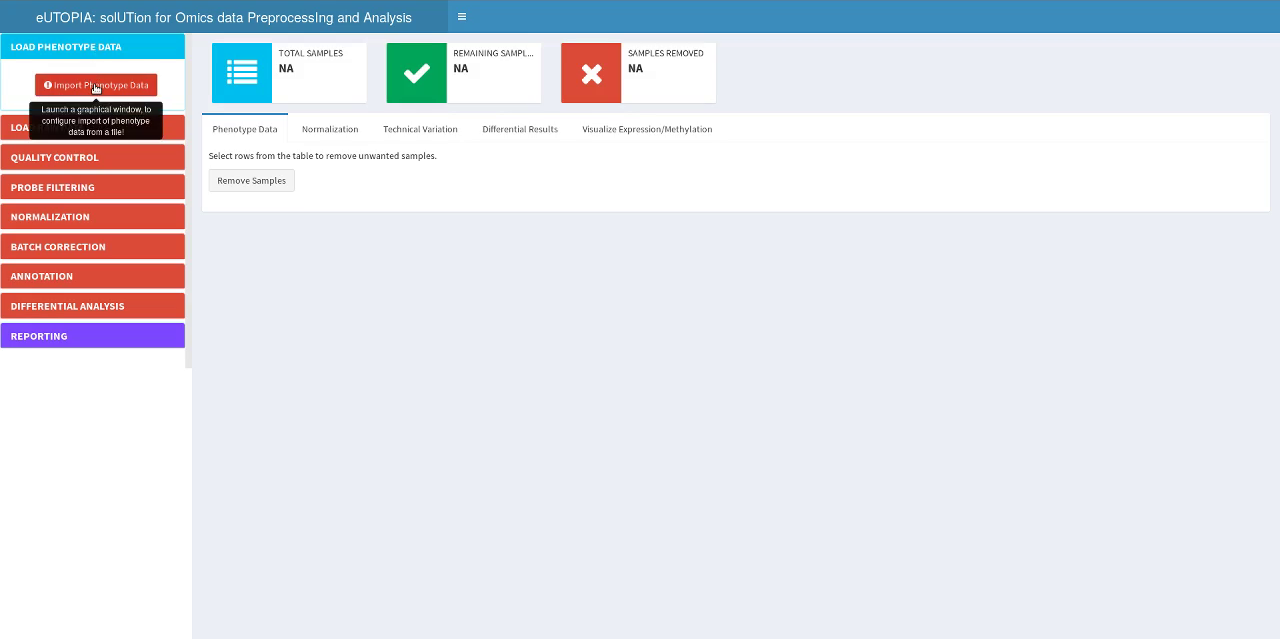


## Select Phenotype File

Browse the file directories and select the file containing the phenotype information.


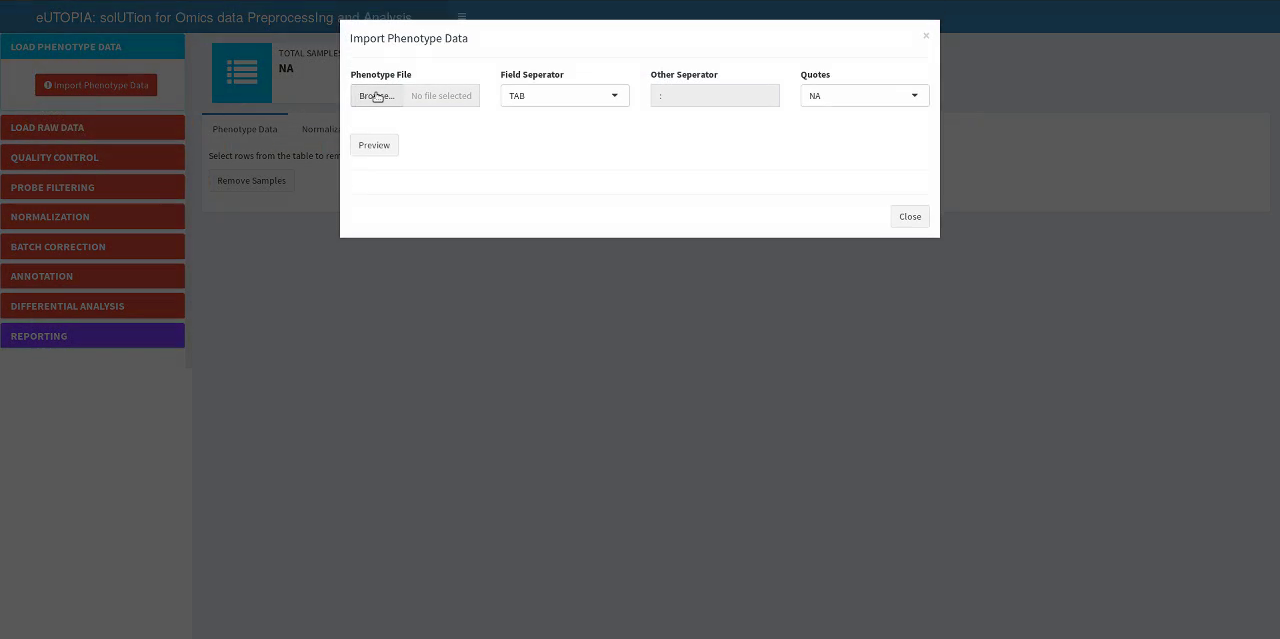


## Specify Field Separator

*Field Separator* options allow the user to specify either tab, comma, semi-colon, space, or other as a separator of columns.


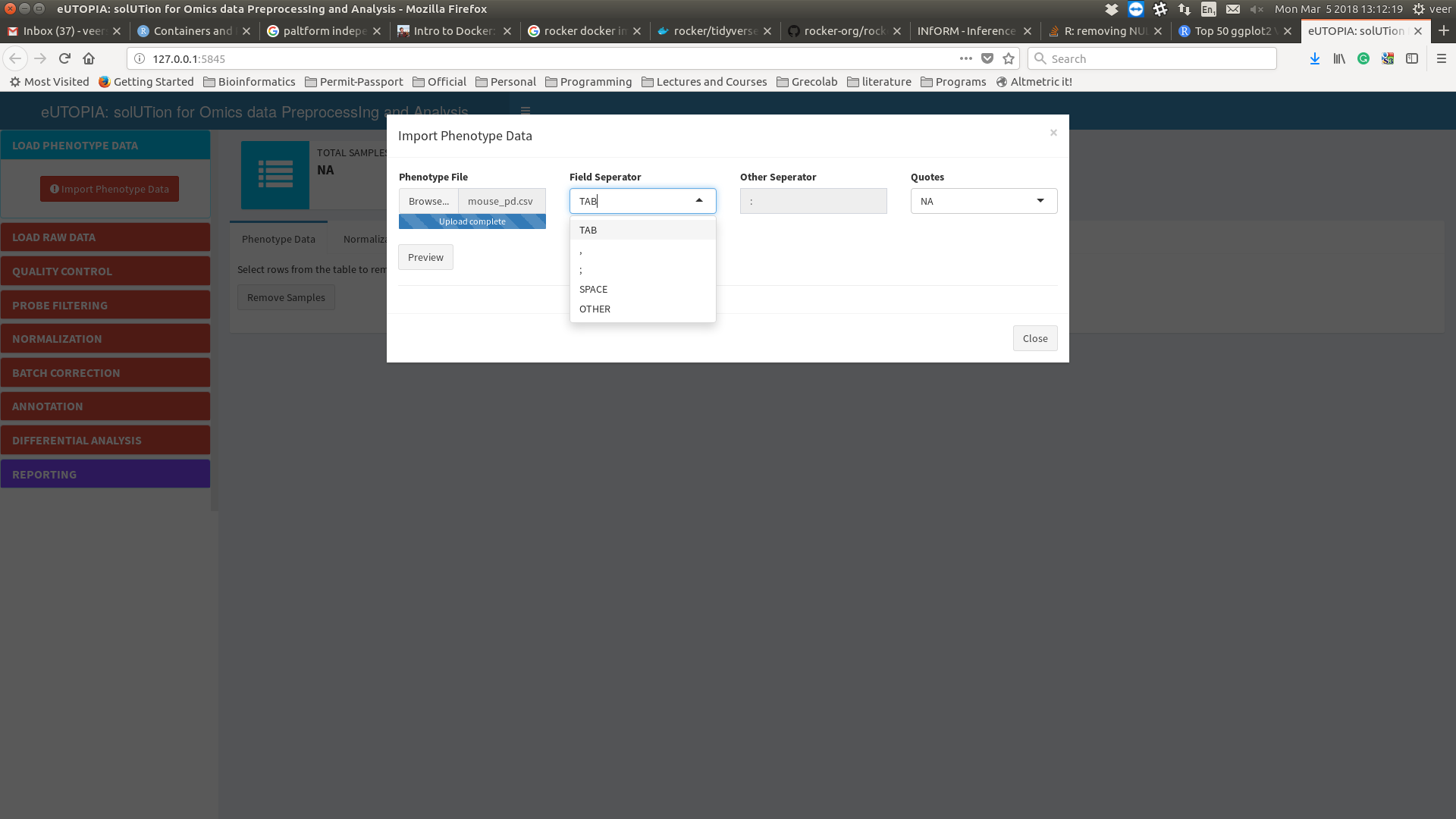


## Custom Field Separator

*OTHER* option enables a free text *Other Separator* input box, where the user can specify any other operator which is not predefined in *Field Separator*.


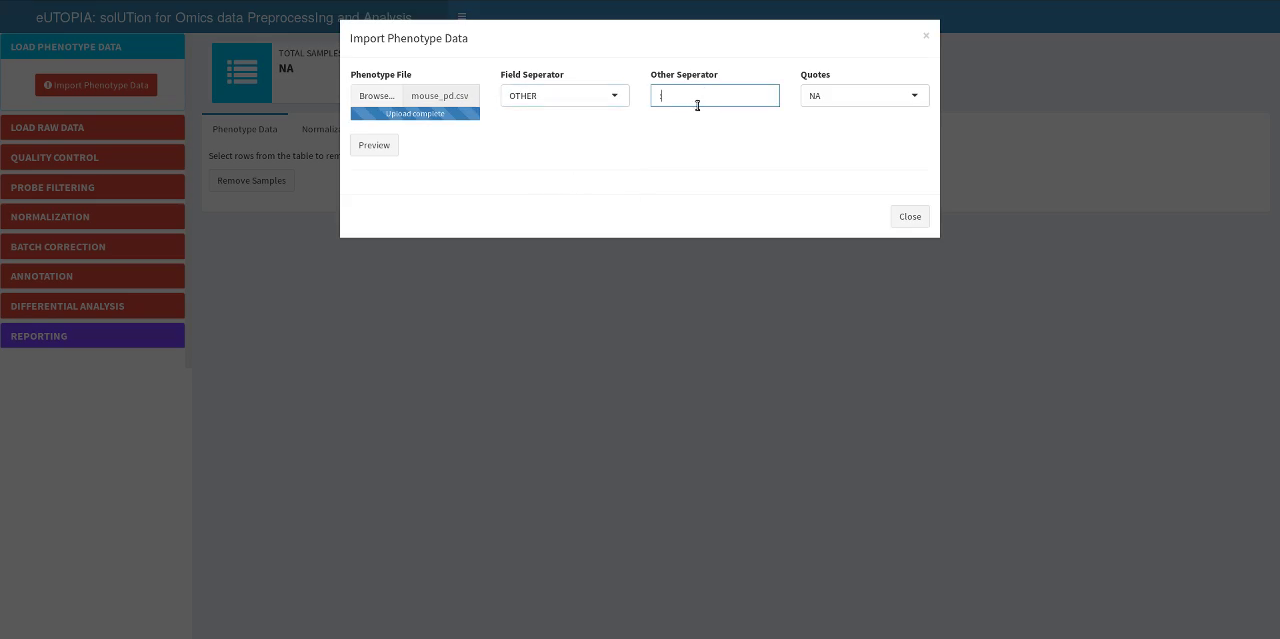


## Specify Quotation Type

In case the input file contains quotations to specify field boundaries then the user can specify either single or double quotes from the *Quotes* input control.


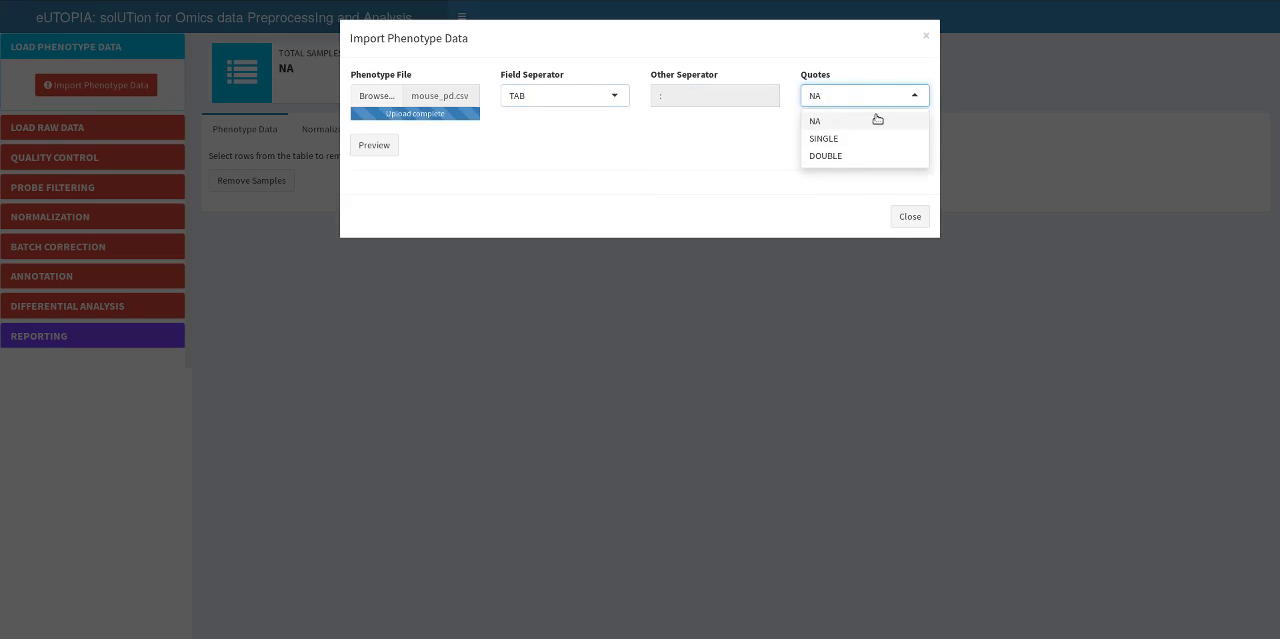


## Phenotype Preview

Preview of the phenotype file displays the columns from the phenotype file as variables. Each variable has an associated R class *character*, *numeric*, or *integer* and data representation type as *factor* or *vector*. Number of samples and variables are reported as text labels above the preview.


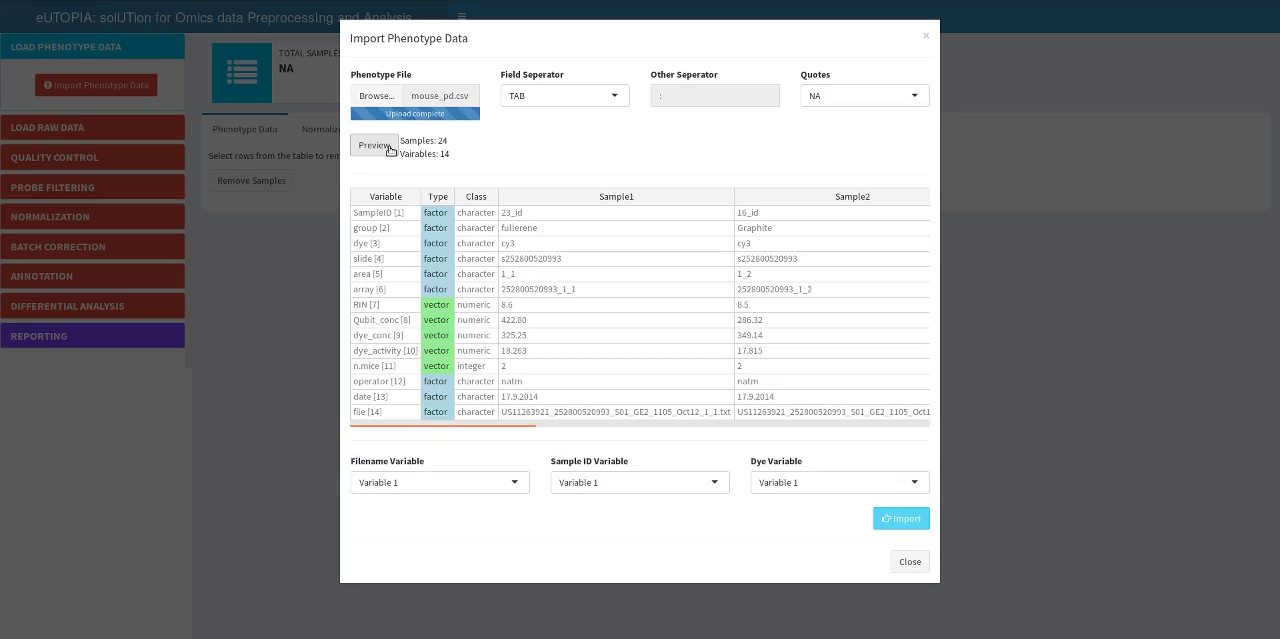


## Configure Variable R Format

The user can change the default data representation type by double-clicking on the representative cell and selecting the alternative option.


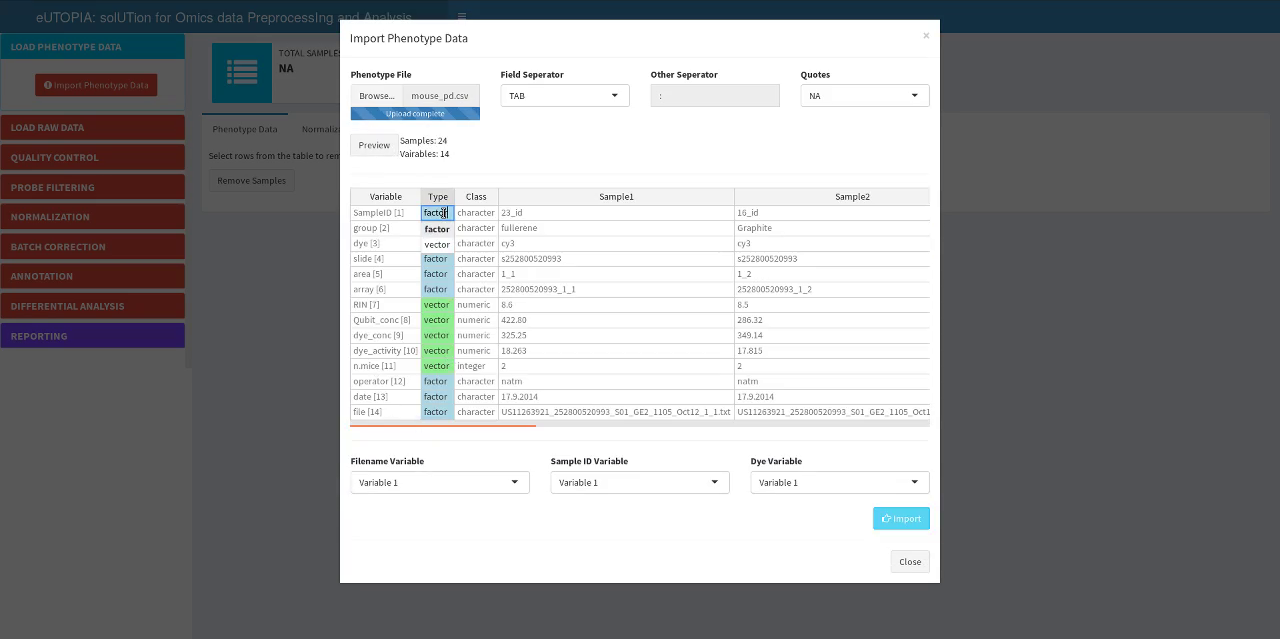


## Specify Filename, Sample ID, and Dye Variables

Platform-specific variables are specified by the corresponding variable index from the phenotype preview.

**
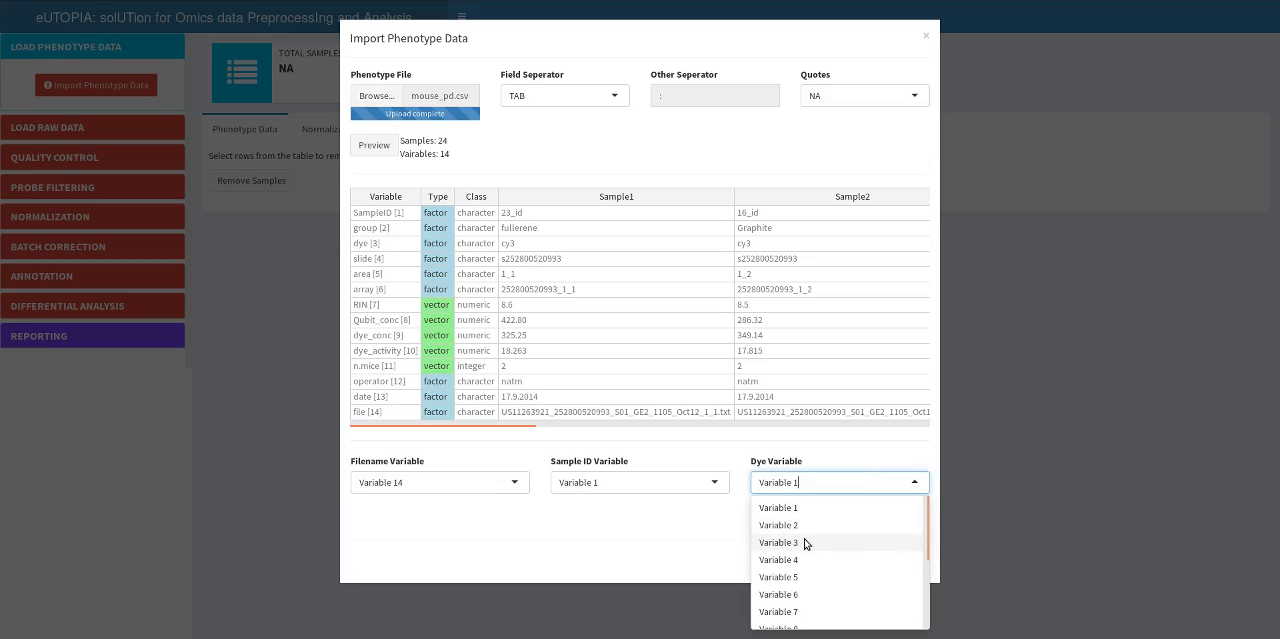
**

## Import Phenotype

Finally, click on the *Import* button to import configured phenotype file.

**
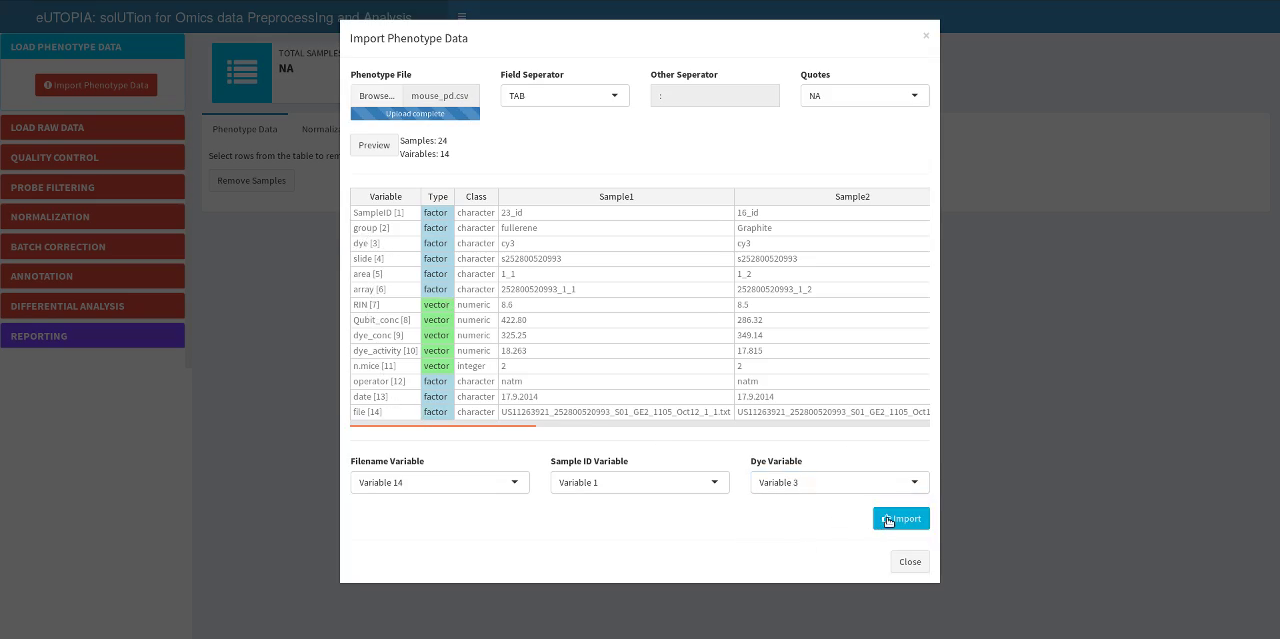
**

## Phenotype View

The imported phenotype file is displayed in the main display area in the main tab *Phenotype Data*.

**
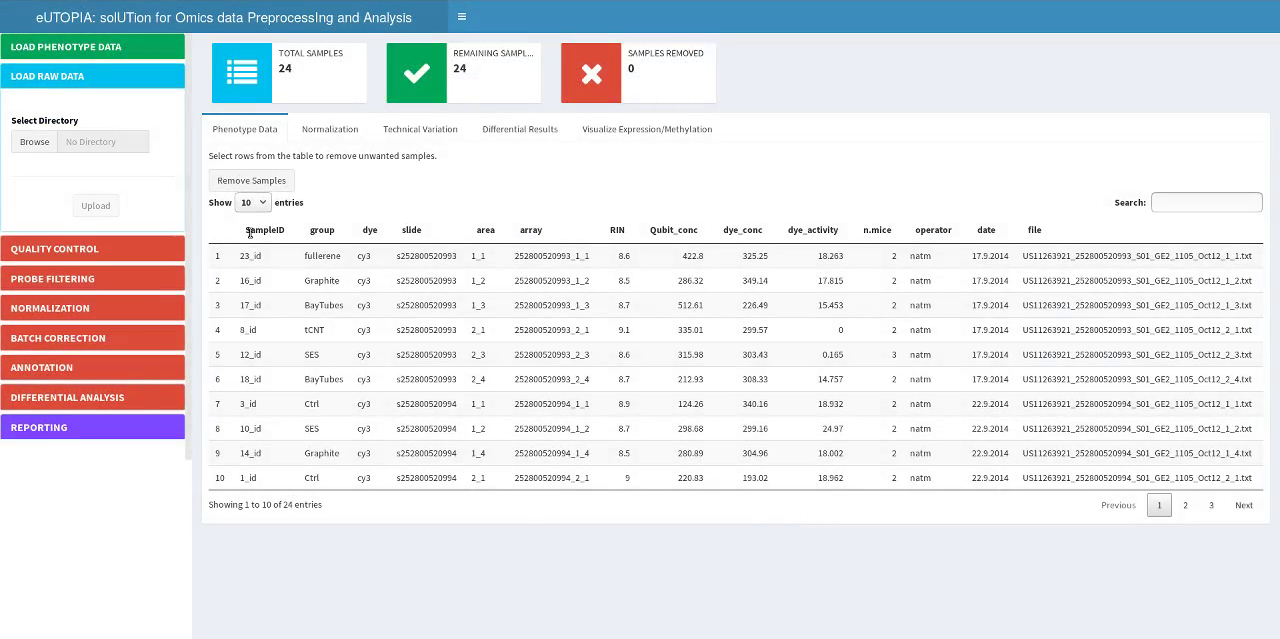
**

# Remove Samples

## Select Samples and Remove

Unwanted samples can be removed from the analysis by selecting the corresponding samples and clicking on *Remove Samples* button.

**
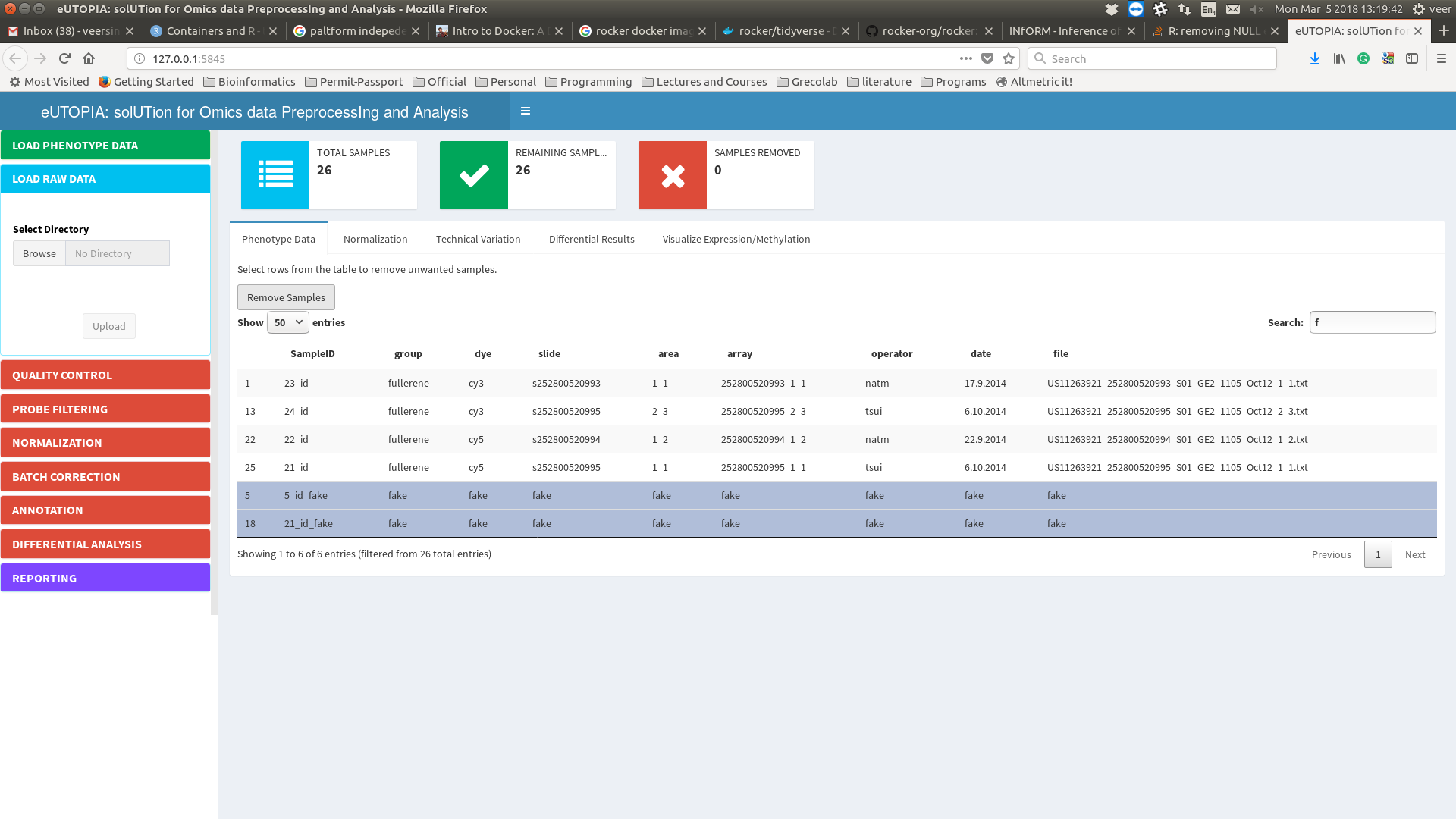
**

## Samples Removed

Sample counts are displayed via the information boxes above the display area.

**
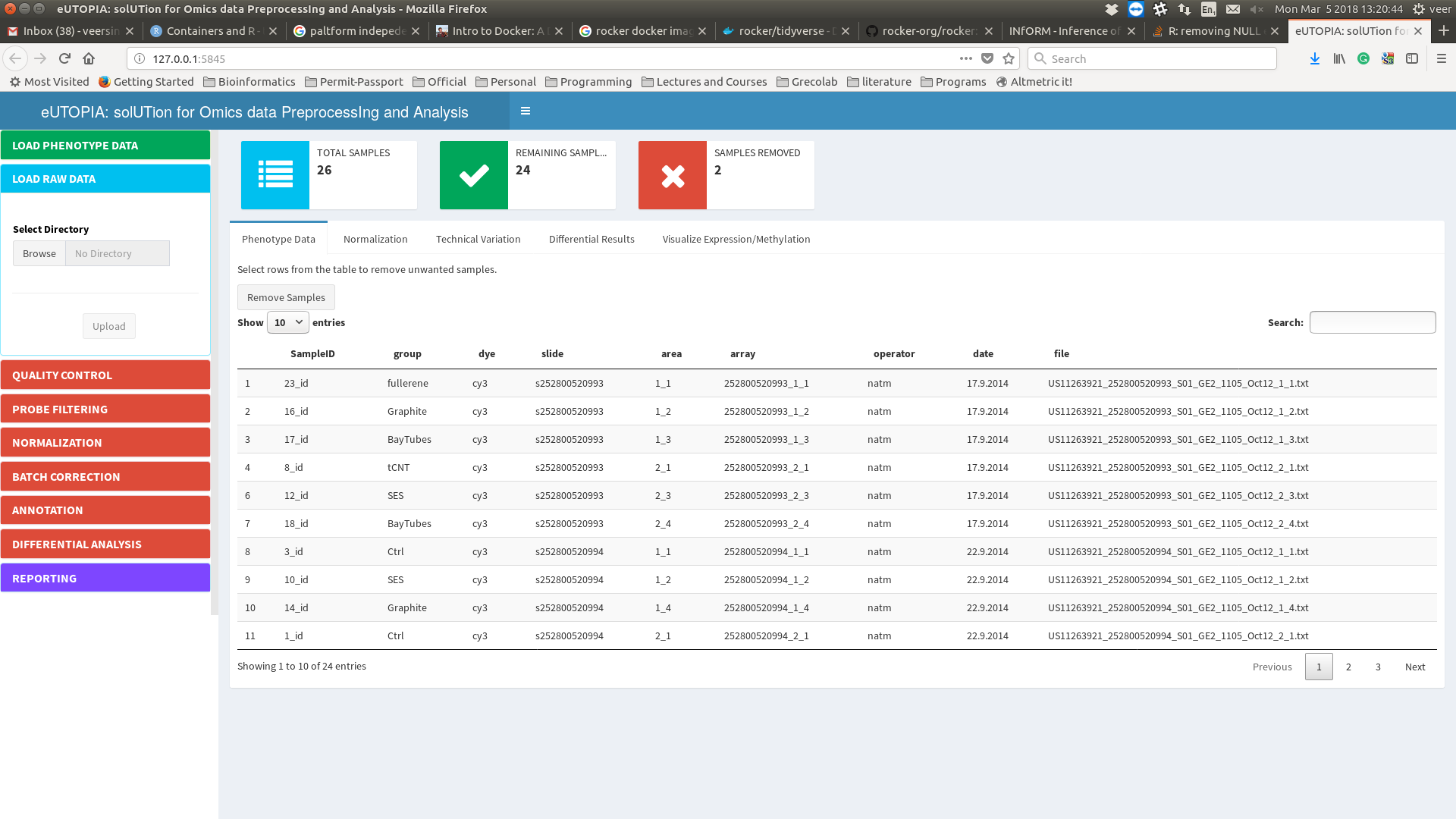
**

# Raw Data

Raw data is uploaded by selecting the directory containing the raw data files. Raw data filenames are obtained from the phenotype file uploaded previously. A directory browser window is launched by clicking on the *Browse* button of the *Select Directory* input control. The user can traverse the directory structure by clicking on the arrowhead symbols to show/hide child directories. Select the directory from the *Directories* pane, the contents of the selected directory are displayed in the adjacent *Content* pane, click on the *Select* button to confirm the selection. Finally, click on the *Upload* button to start the upload process. This step has an added annotation specification section for Affymetrix data (not shown here).

| Browse Directory | Select Raw Data Directory | Upload Raw Data |
| --- | --- | --- |
| **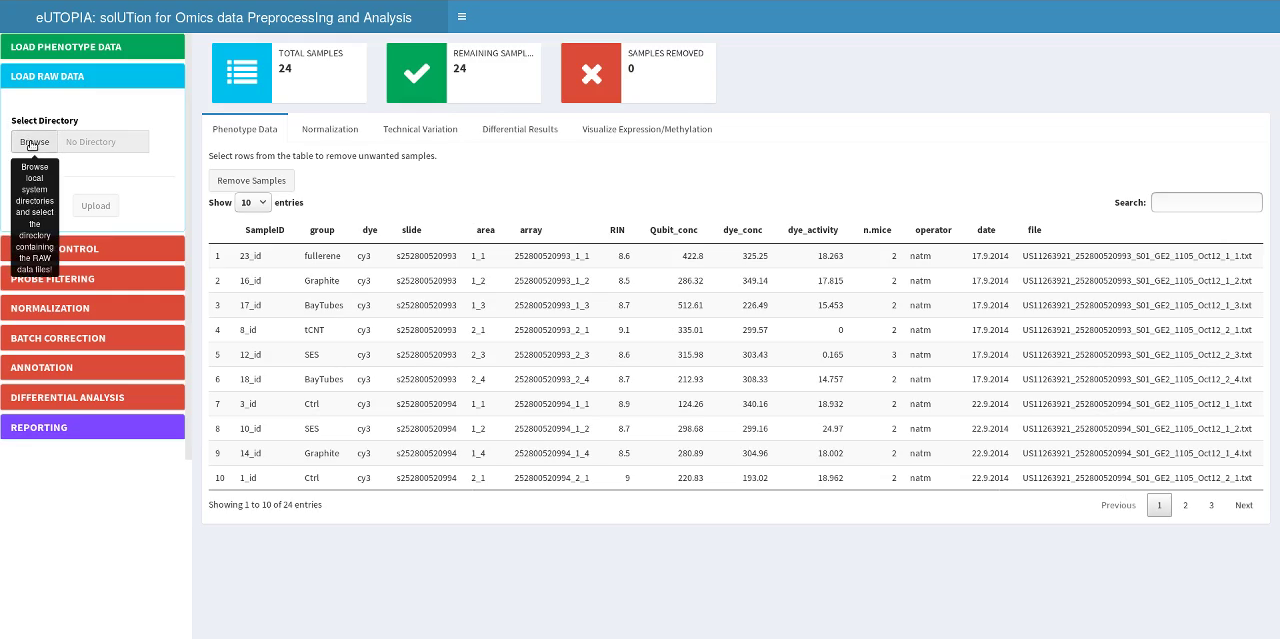** | **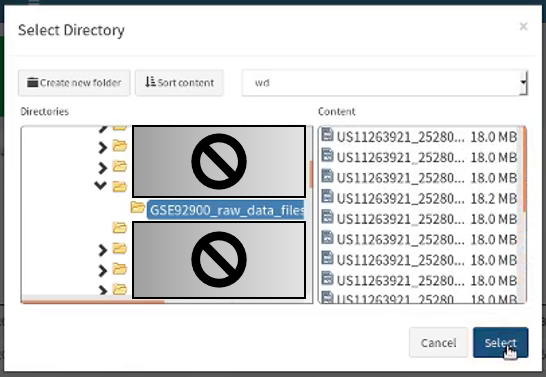** | **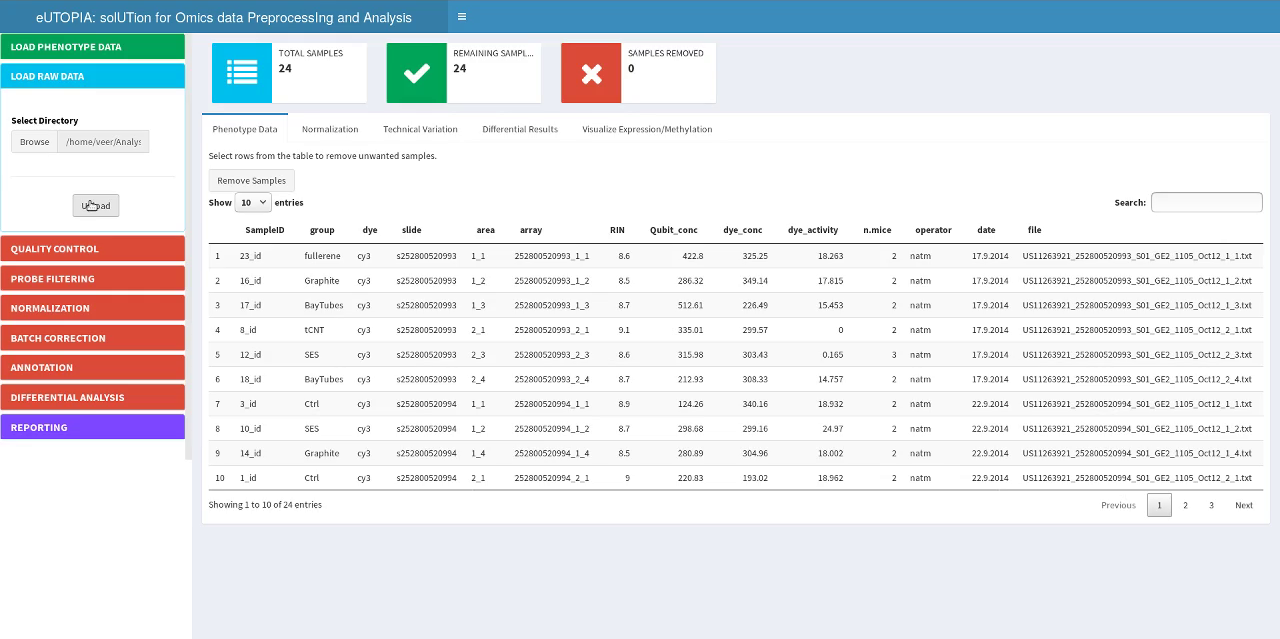** |

# Quality Control

QC report can be generated as PDF or an archived HTML report; alternately the user can choose to skip the QC step.

| Perform QC or Skip | Save QC Report |
| --- | --- |
| **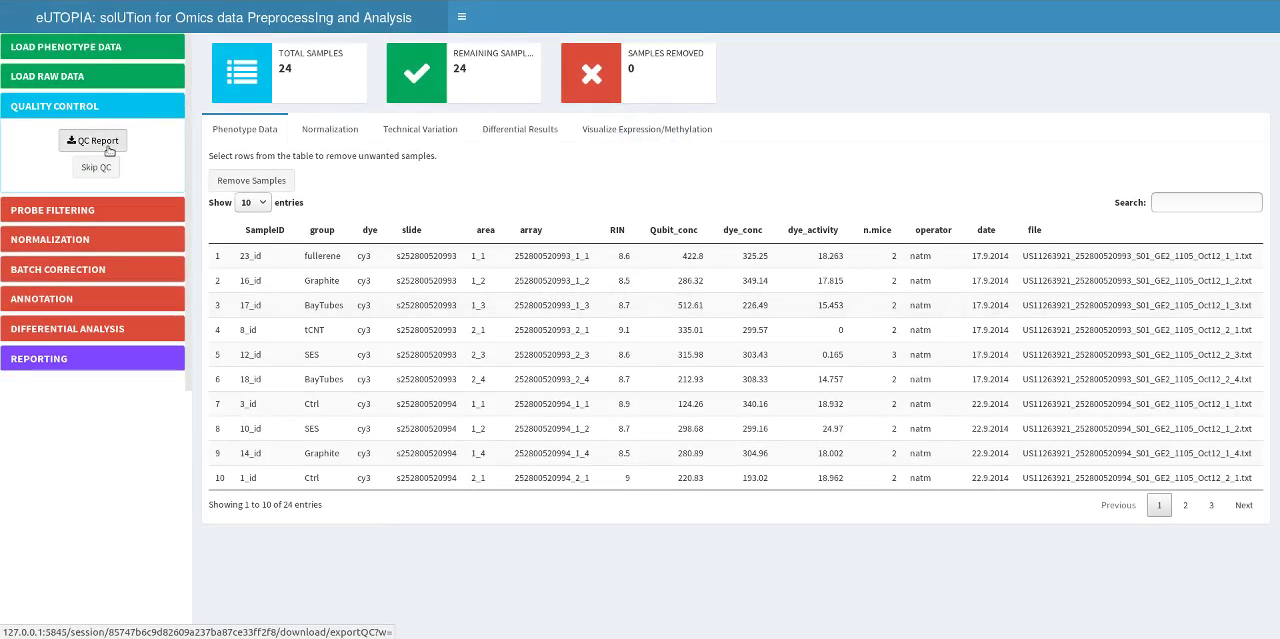** | **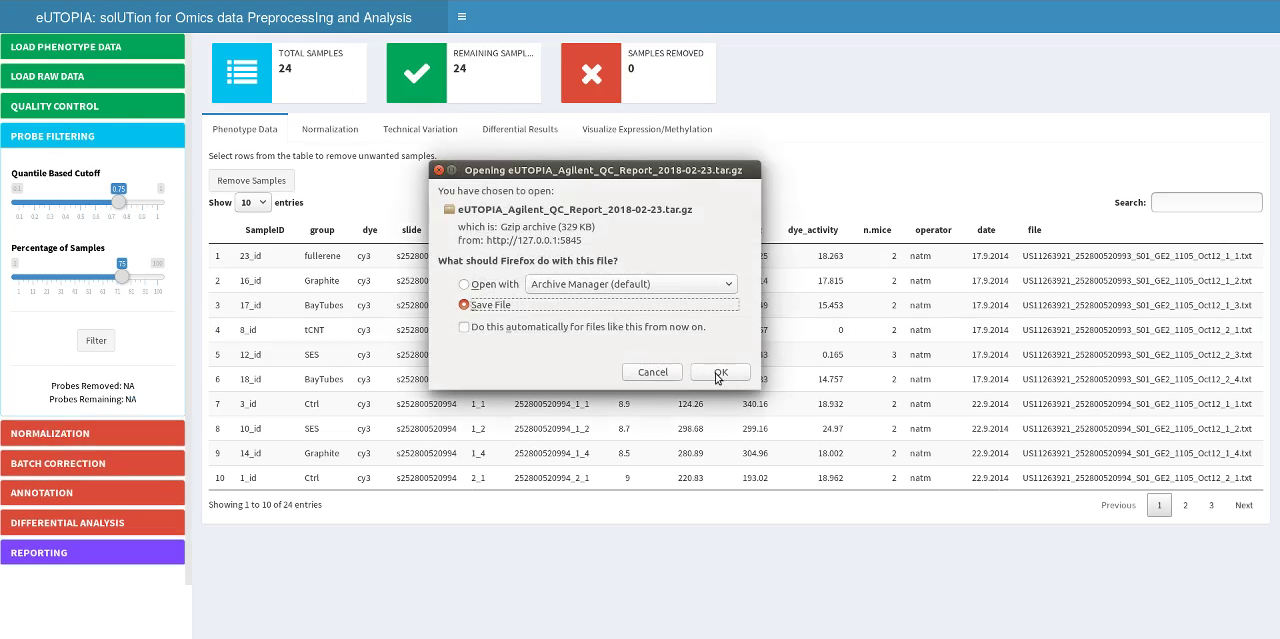** |

# Filter Probes

## Filtering Options

Control probe-based filter is provided for Agilent platforms. The user can set filter parameter *Quantile Based Cutoff* to set the expression value in negative control probes corresponding to the specified quantile as the cutoff value for validation of normal probes. *Percentage of Samples* parameter specifies the percentage of samples over which the probe should be validated to be greater than or equal to the specified cutoff expression value. Eg., *Quantile Base Cutoff* 0.75 means set the 75^th^ percentile of the expression value distribution in negative control probes is used as the cutoff, *Percentage of Samples* 75 means check that at least 75% samples have expression value greater than or equal to the cutoff value for each normal probe. Normal probes failing this filter are removed and the filtered set is taken forward for analysis. Different options are provided for filtering Illumina methylation platform data (not shown here).

| Specify Expression Quantile | Specify Percentage of Samples | Perform Filtering |
| --- | --- | --- |
| **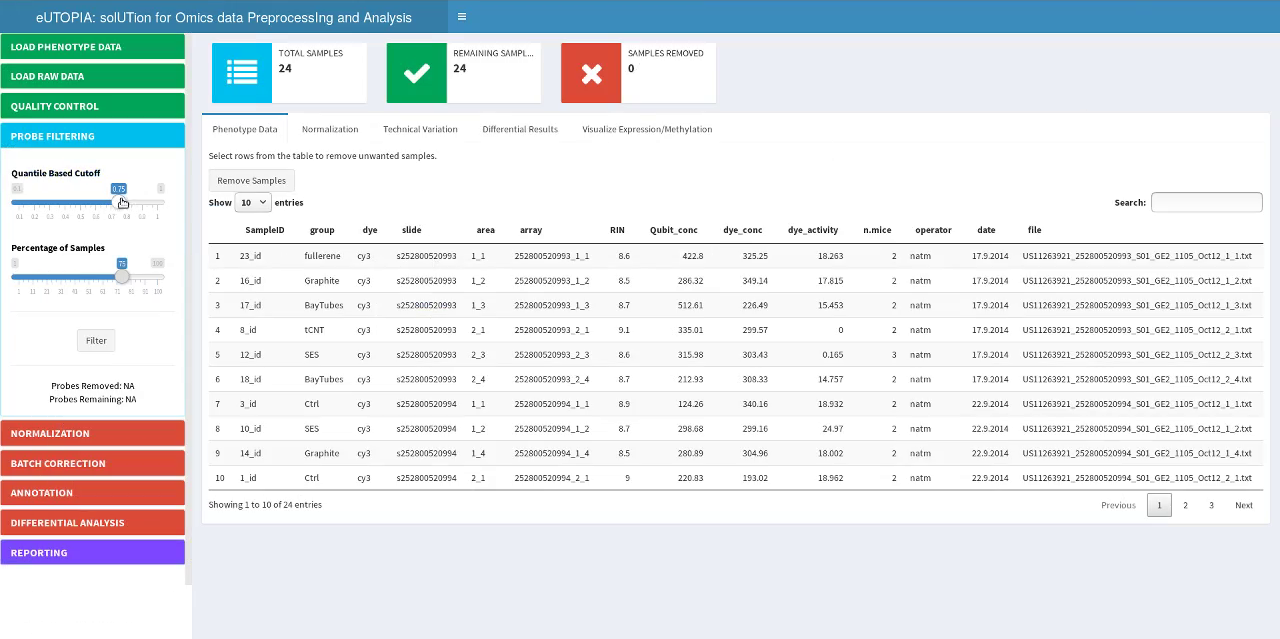** | **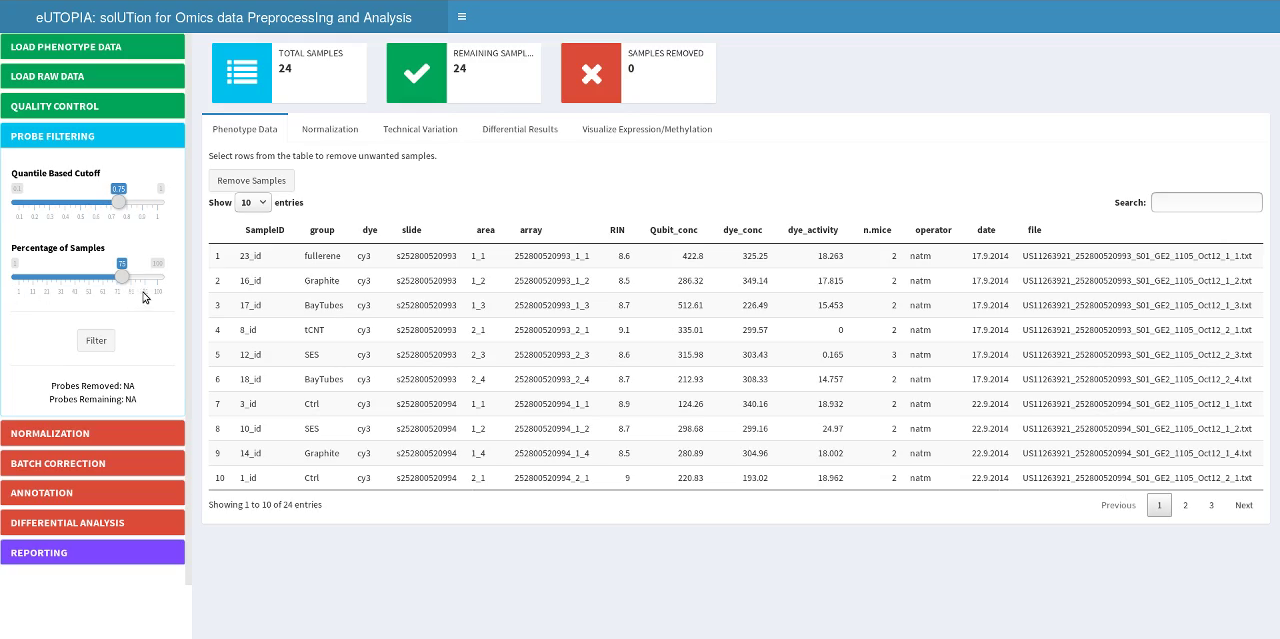** | **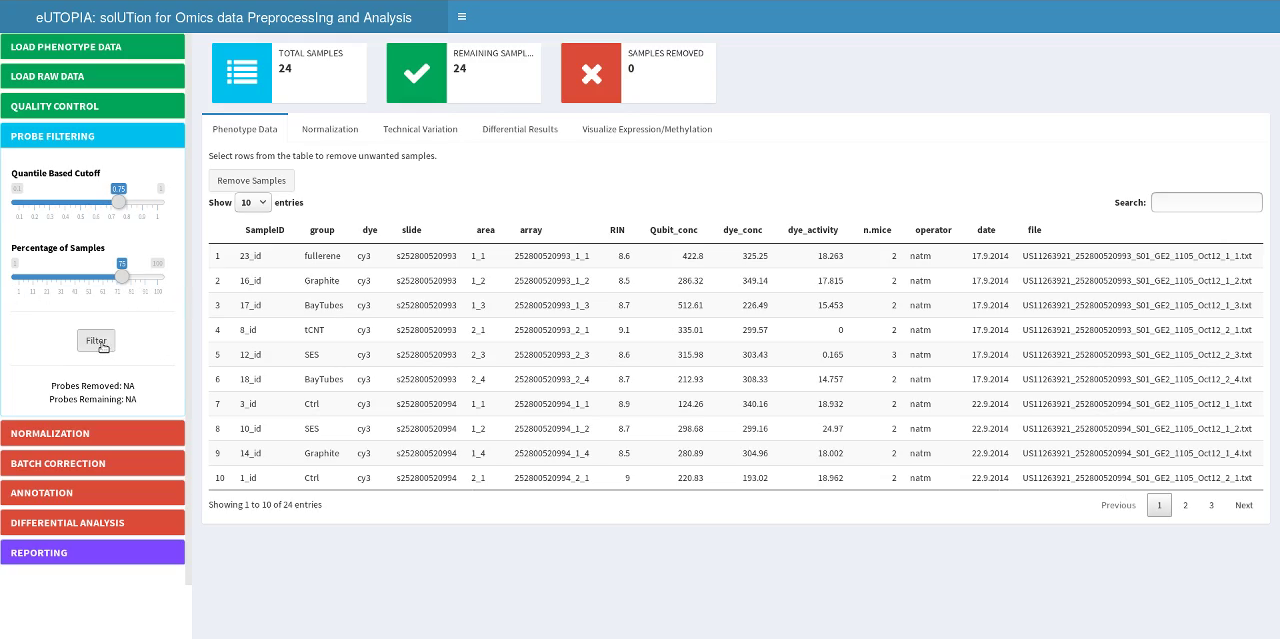** |

## Filtering in Progress

**
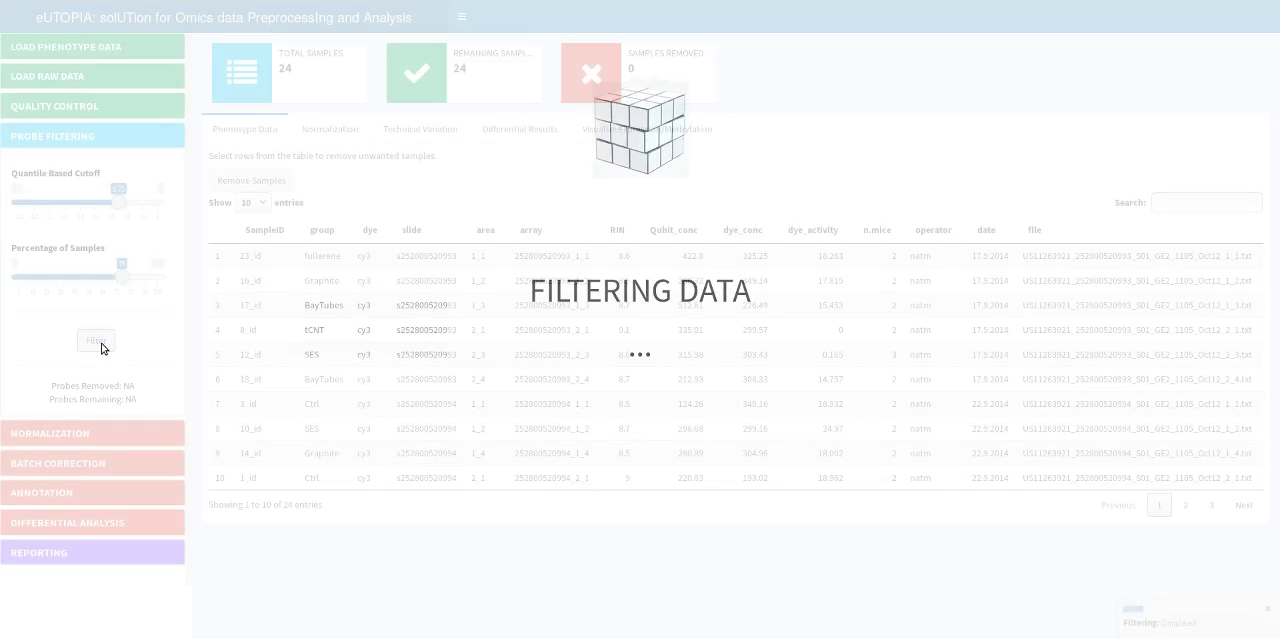
**

# Normalization

## Normalization Options

Methods from Limma R package are provided for normalization of data. The parameter *Normalization Type* provides the user with four options; if the user chooses *Between Arrays* option, then further method specification can be specified in the *Method* parameter.

| Select Normalization Type | Select Normalization Method | Run Normalization |
| --- | --- | --- |
| 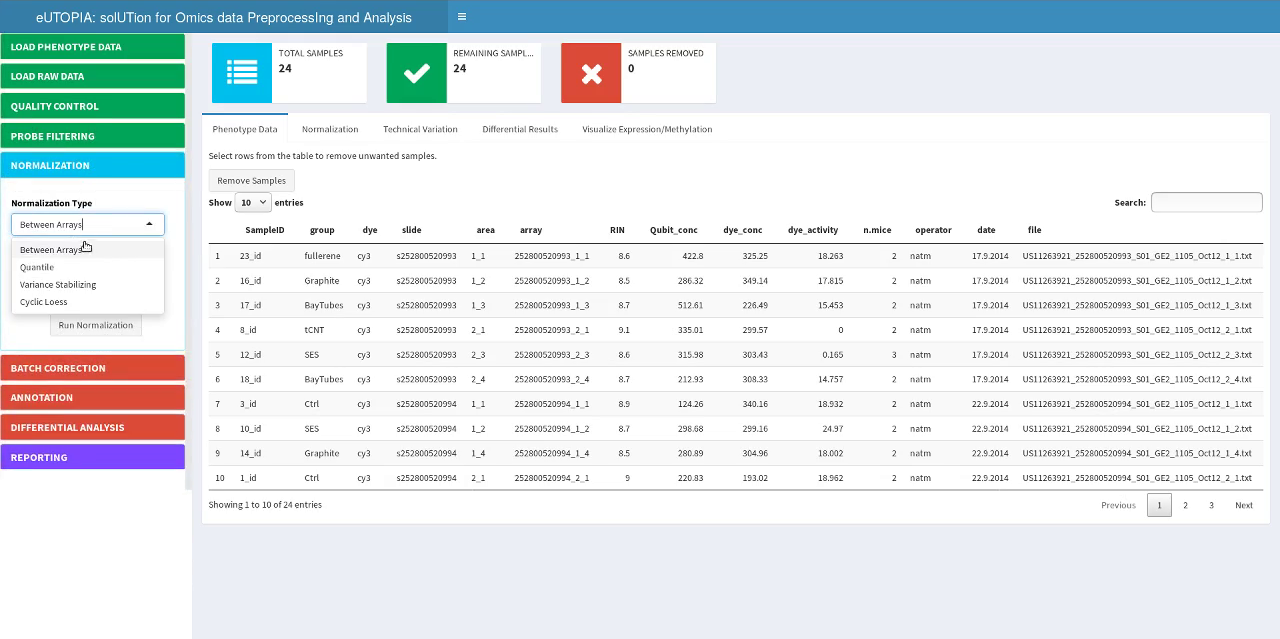 | **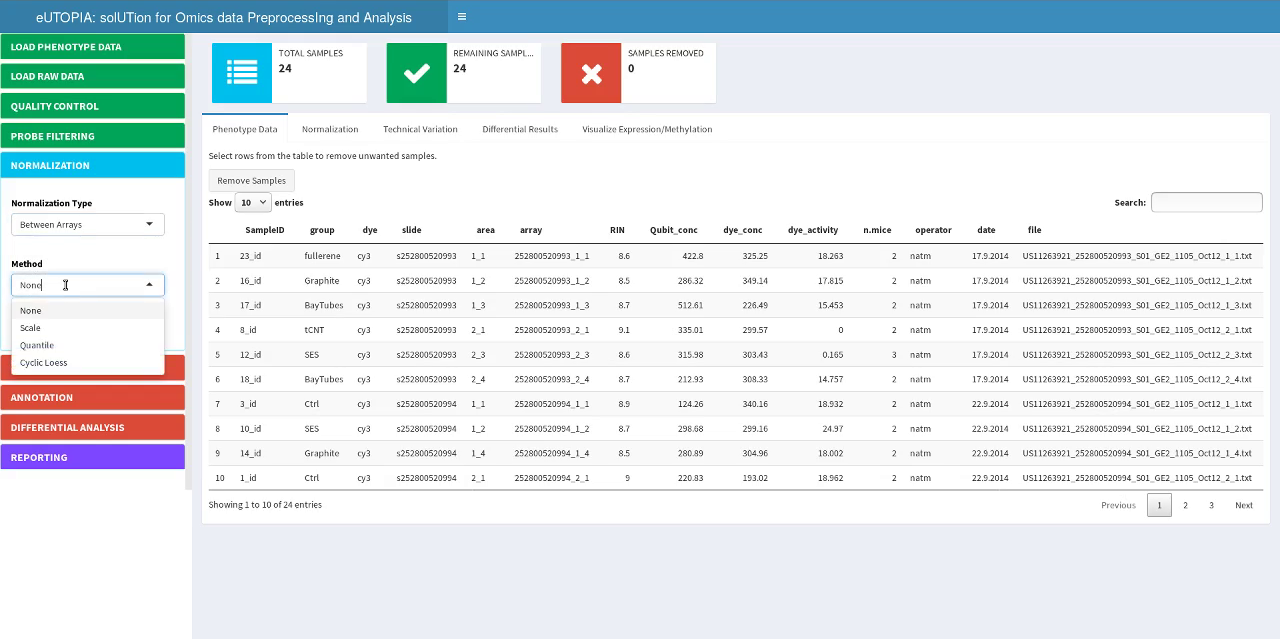** | **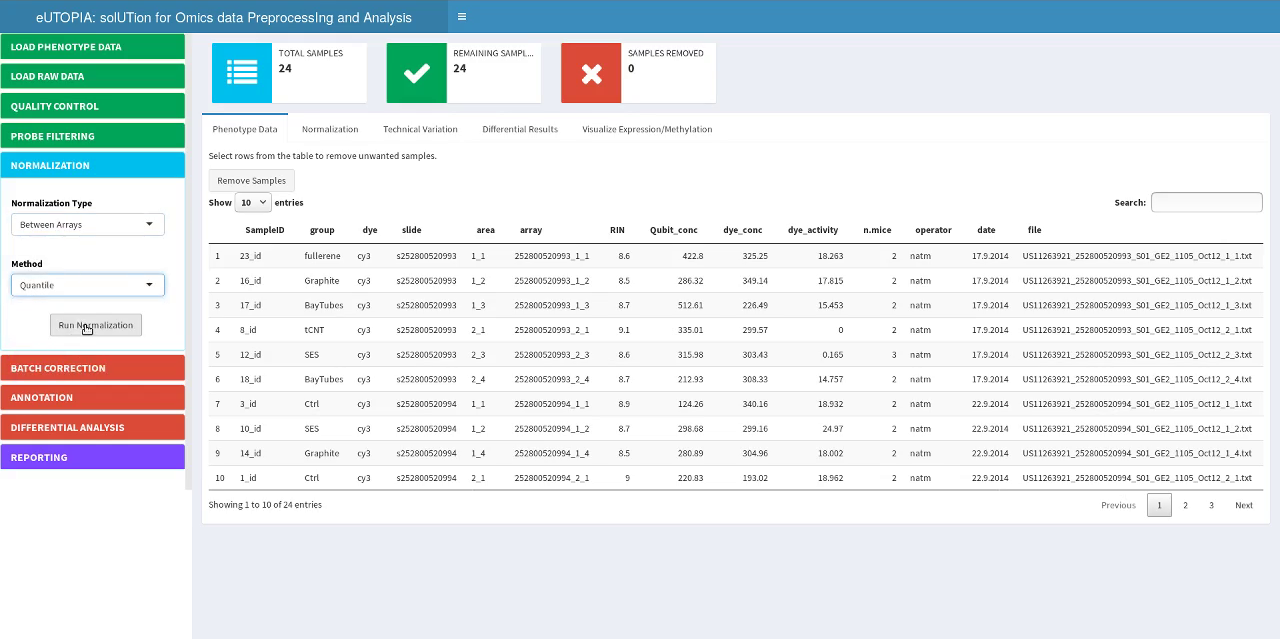** |

## Normalization in Progress

**
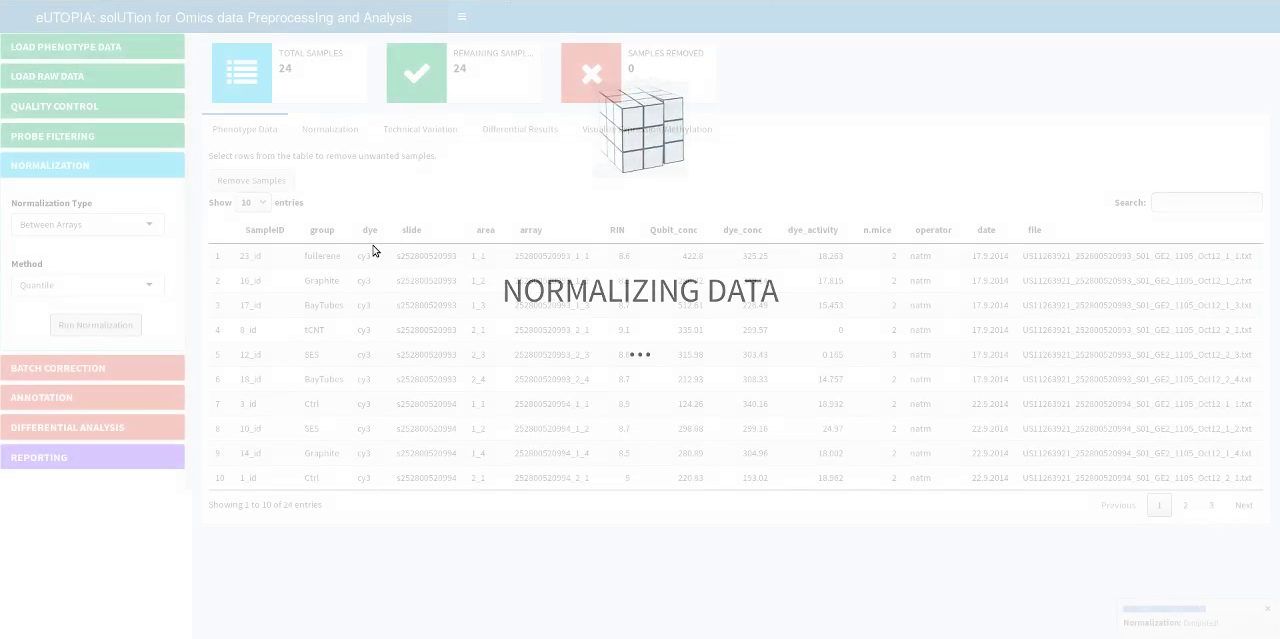
**

# Normalization Plots

## Box Plots

Box plots for the expression values before and after normalization can be viewed from the sub-tab *Box Plot* nested within the *Normalization* tab.

**
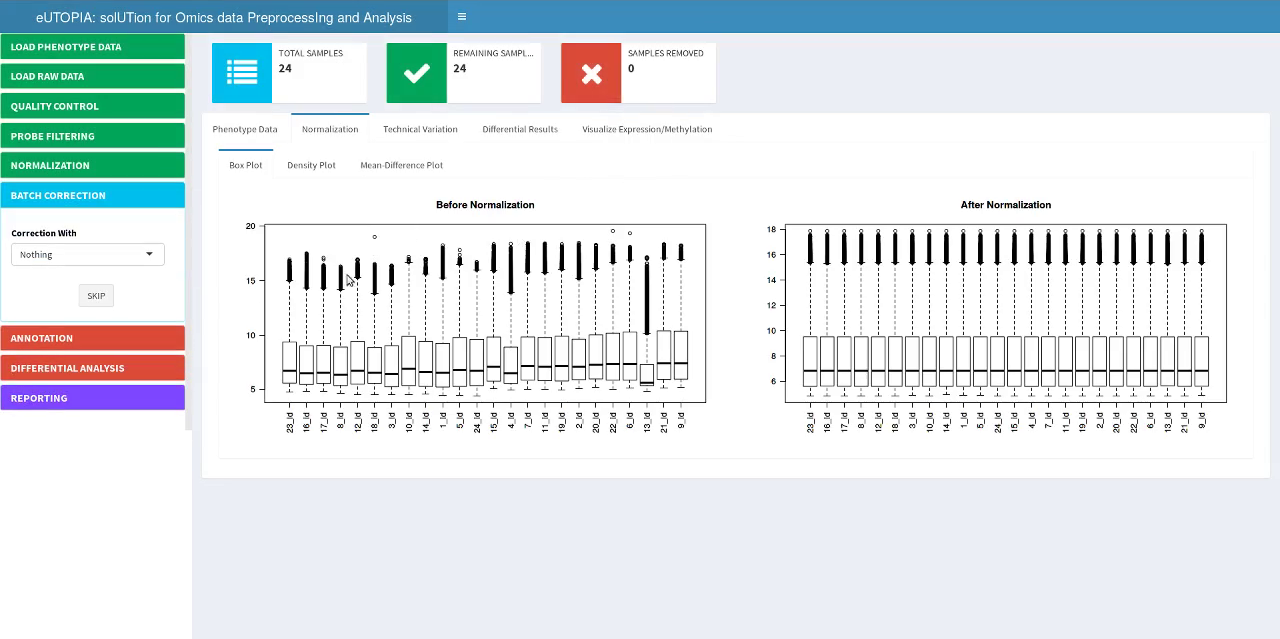
**

## Density Plots

Density plots for the expression values before and after normalization can be viewed from the sub-tab *Density Plot* nested within the *Normalization* tab. For Agilent 2-color platform this plot shows the expression density in red and green channels, for Illumina methylation (450k and EPIC) platform it shows the density of the Beta values (Methylated signal over total Methylated+Unmethylated signal). This plot is not available for Agilent 1-color and Affymetrix expression platforms.

**
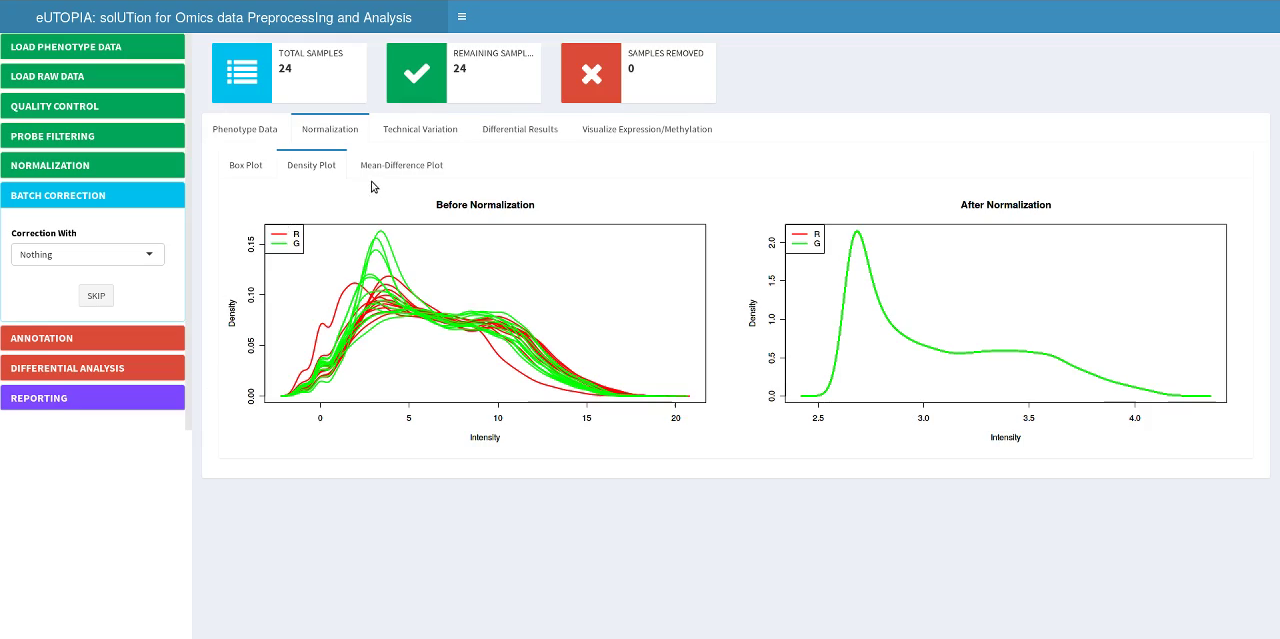
**

## Mean Difference Plots

Mean Difference plots for the expression values before and after normalization can be viewed from the sub-tab *Mean Difference Plot* nested within the *Normalization* tab.

**
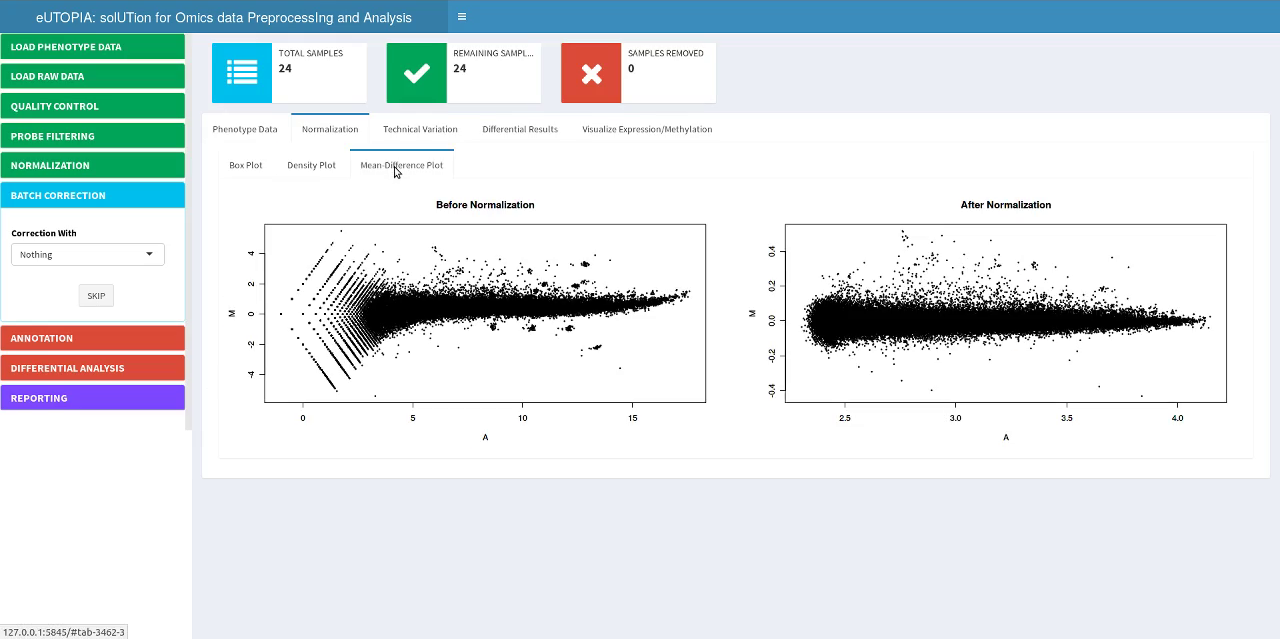
**

# Technical Variation

## Confounding Plot

Confounding plot displays the interrelatedness between the variables of the phenotype data; this is represented as a heatmap with correlation p.value represented as text and color gradient where red is highly correlated and white is non-correlated. This plot can be viewed from the sub-tab *Confounding Plot* nested within the *Technical Variation* tab. The p-value of interrelatedness is printed as text label in each cell and it is also represented as the color corresponding to the heatmap scale.

**
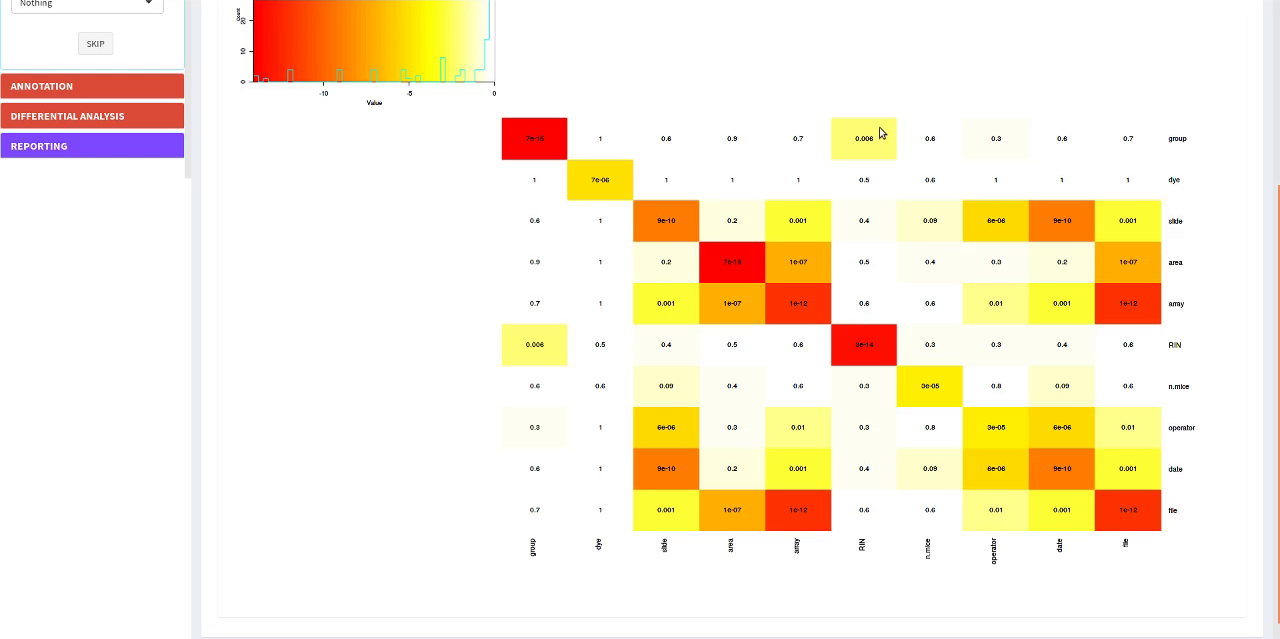
**

## Prince Plot

Prince plot displays the association between the phenotype variables and the principal components; this is represented as a heatmap where principal components are on the x-axis and phenotype variables on the y-axis, the correlation p.value is presented as text and color gradient where red is highly correlated and white is non-correlated. On the x-axis principal component label contains the percentage of variability associated to them in brackets, this information along with correlation p.value is used to identify the phenotype variables representing the most variability. This plot can be viewed from the *Before Correction* tab nested within the sub-tab *Prince Plot* nested within the *Technical Variation* tab. The heatmap displays the p-value computed by linear regression as text label in each cell and it is also represented as the color corresponding to the heatmap scale.

**
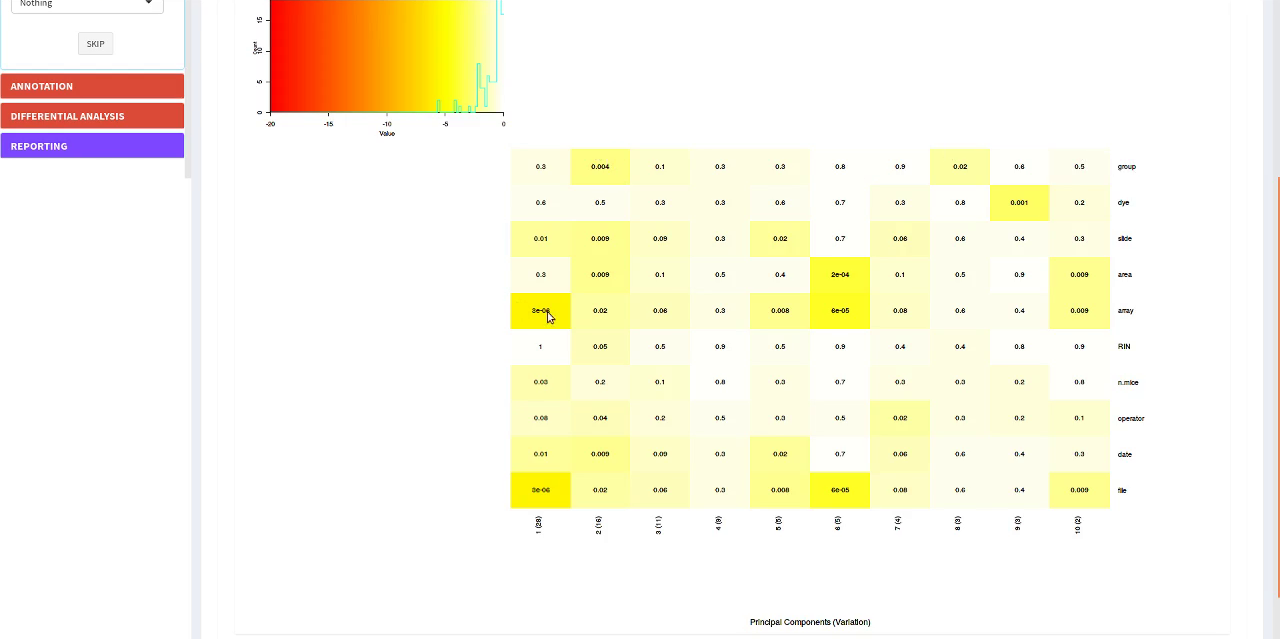
**

## Hierarchical Clustering Plot

Hierarchical clustering plot displays the clustering of the samples based on the expression profile, sample annotation provided in the phenotype variables is plotted below the cluster as color-coded bars. The color code is used to see the distribution of groups in the phenotype variables, groups in the phenotype variable of interest should be separated discretely. This plot can be viewed from the *Before Correction* tab nested within the sub-tab *Hierarchical Clustering* nested within the *Technical Variation* tab.

**
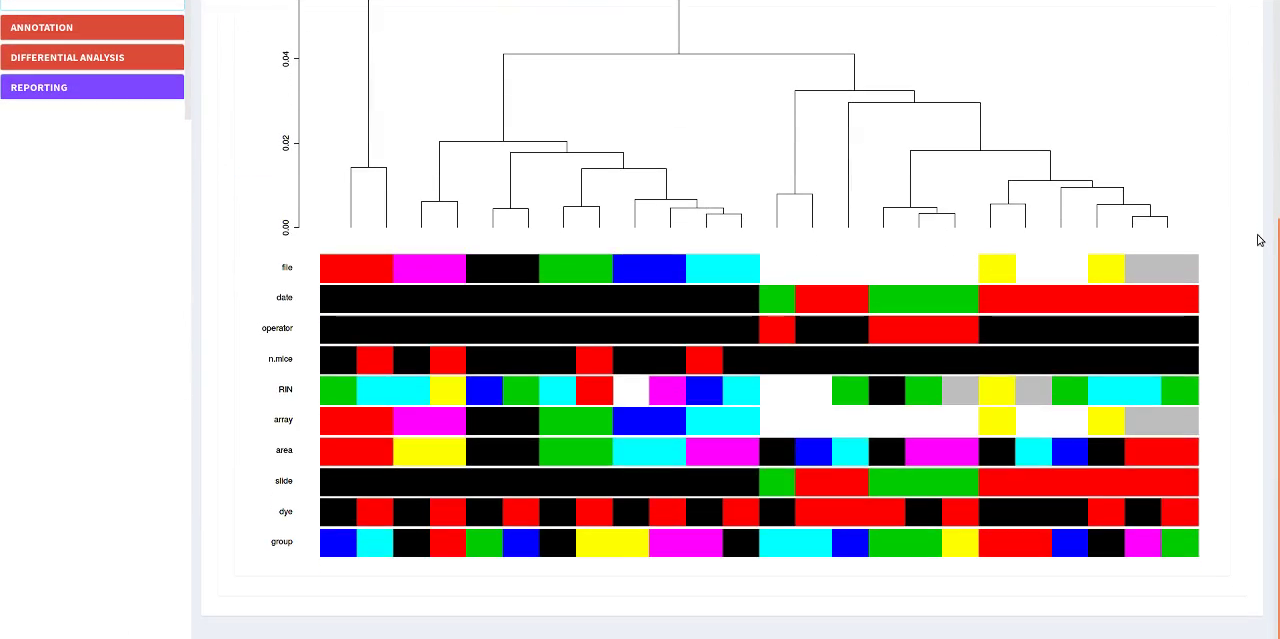
**

## Multi-Dimensional Scaling (MDS) Plot

Multi-Dimensional Scaling plot displays the distance between each pair of samples; it represents the log2 fold change between the samples as observed by the top selected genes. The two-dimensional scatterplot can be represented with phenotype variables as label text and label color. Samples from the same group in the phenotype variable of interest should be closer together while the samples from other groups in the phenotype variable should be distant which would represent the differential expression profile of the genes in these two groups of samples. This plot can be viewed from the *Before Correction* tab nested within the sub-tab *MDS Plots* nested within the *Technical Variation* tab.

**
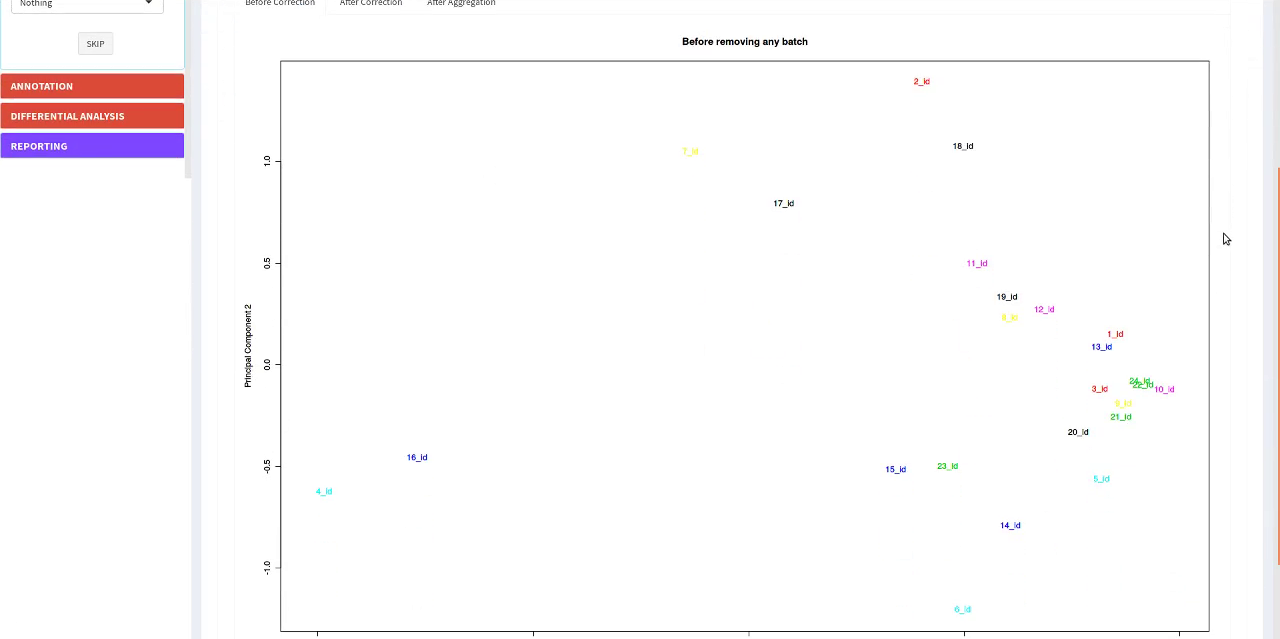
**

## MDS Plot Options

The label text can be specified from the *MDS Label Name* input and label color representation can be specified from the *MDS Label Color* input control.

**
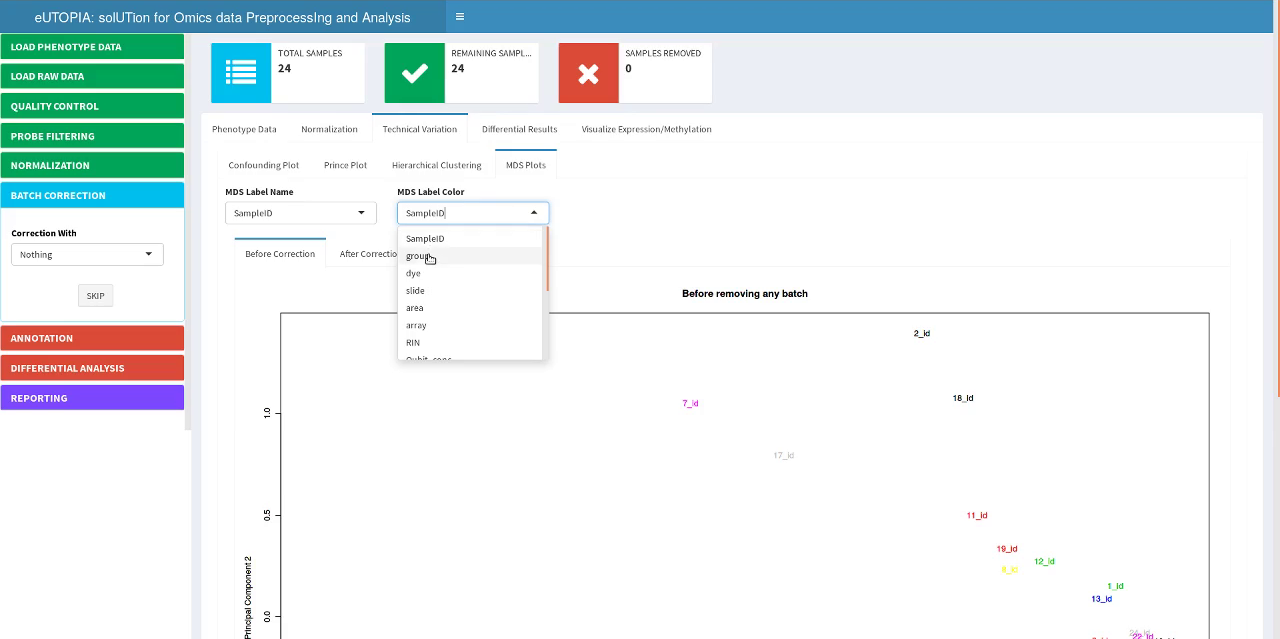
**

# Batch Correction

## Batch Correction Options

Batch correction is performed by ComBat for known variables or surrogate variables identified by SVA. The user has four different strategies to choose from *Nothing*, *SVA+ComBat*, *ComBat*, and *SVA*. Nothing option skips this step, *SVA+ComBat* option enables *Launch SVA Module* and *Launch ComBat Module* buttons, while *SVA* and *ComBat* options enables their corresponding Launch Module buttons. *ComBat* option is for correction of known variables, *SVA* option identifies surrogate variables that can be used as covariates in limma model definition while performing differential analysis, and *SVA+ComBat* option is used to first identify surrogate variables by SVA followed by correction of surrogate variables by ComBat.

| Different Options | ComBat Option | SVA+ComBat Option | SVA Option |
| --- | --- | --- | --- |
| **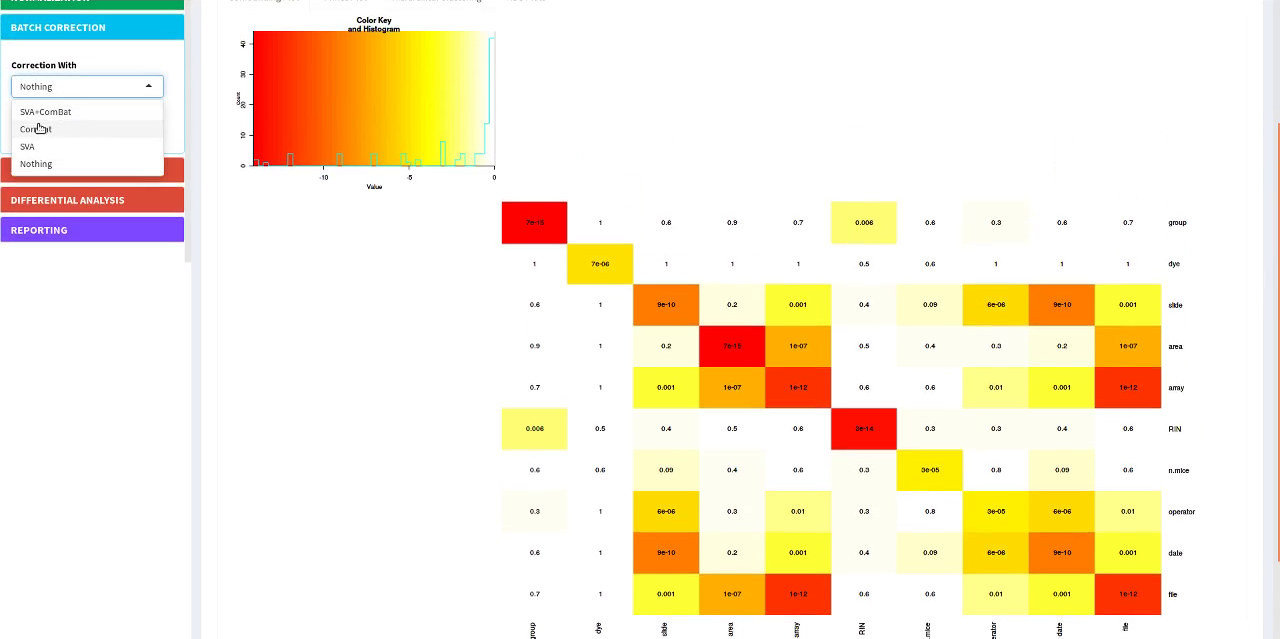** | **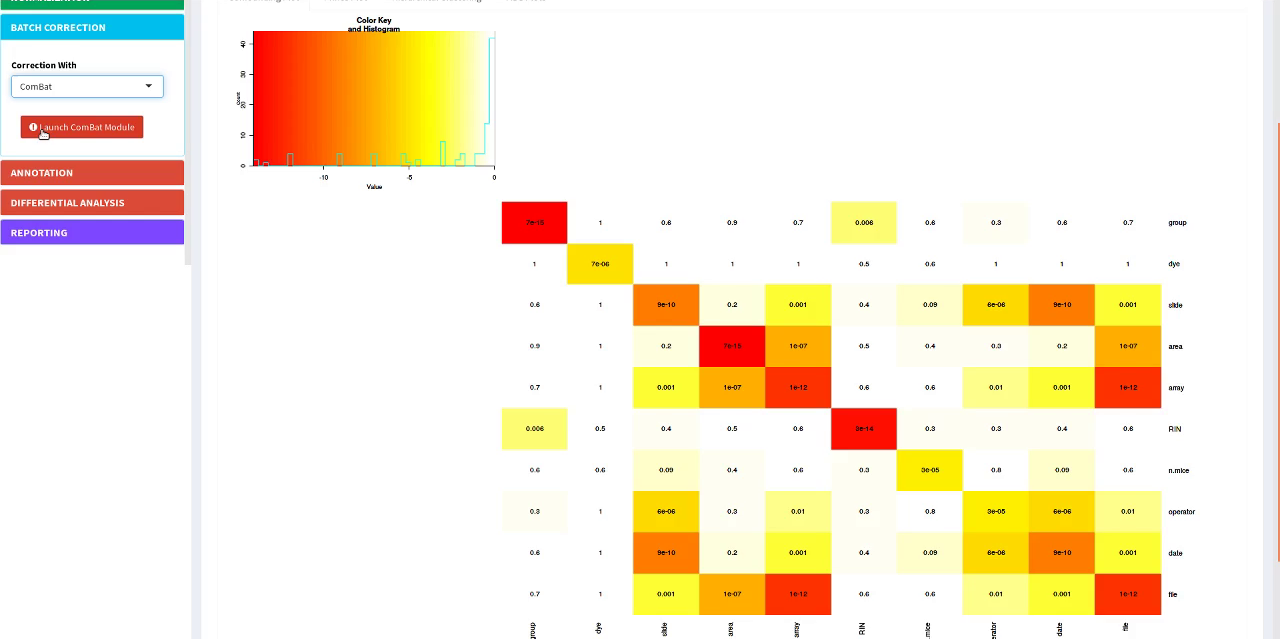** | **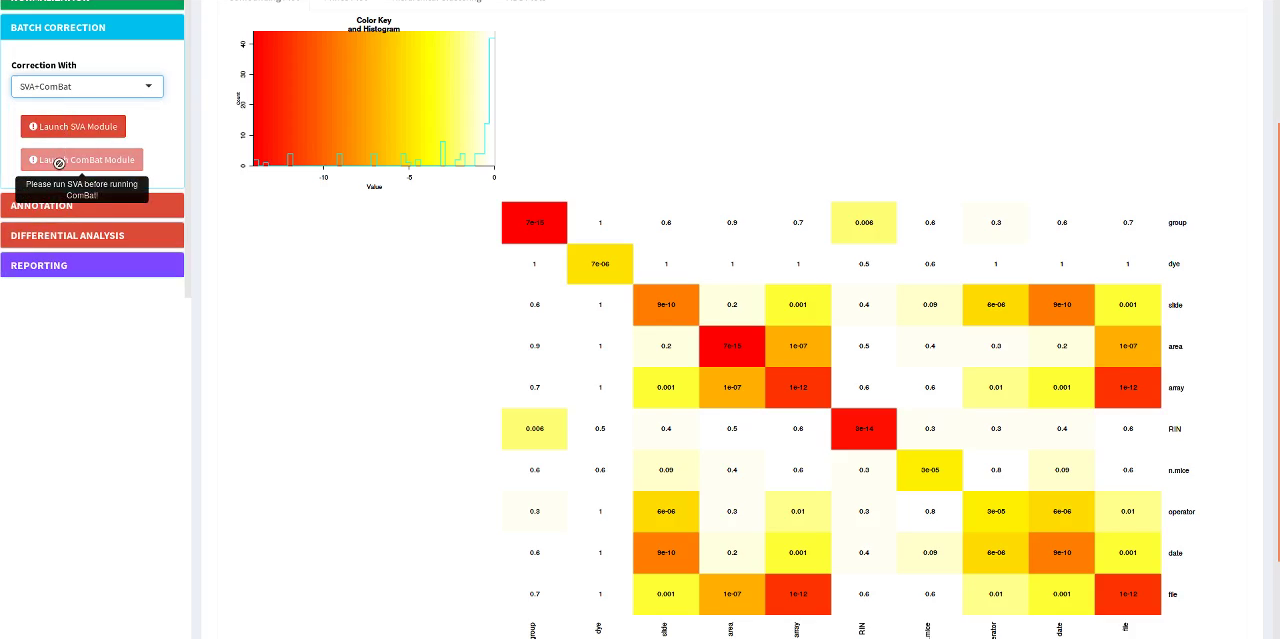** | **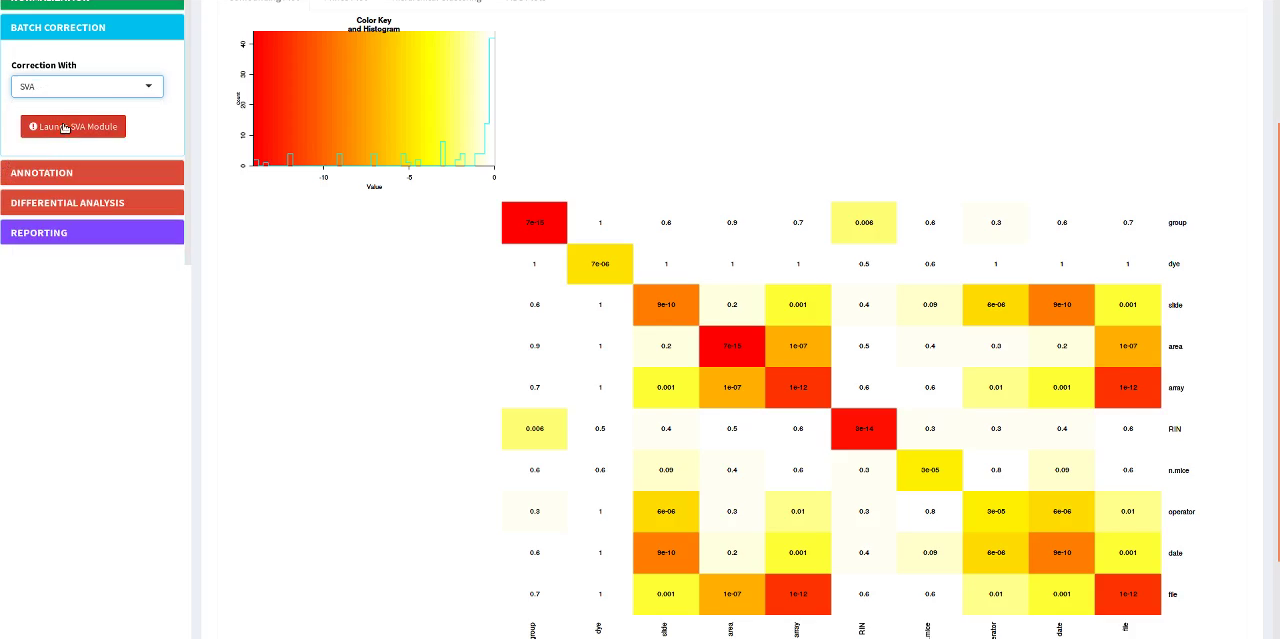** |

# Known Batch Correction

## ComBat Configuration

In the ComBat module window, user can specify the variable of interest, co-variates, and batches for correction.

**
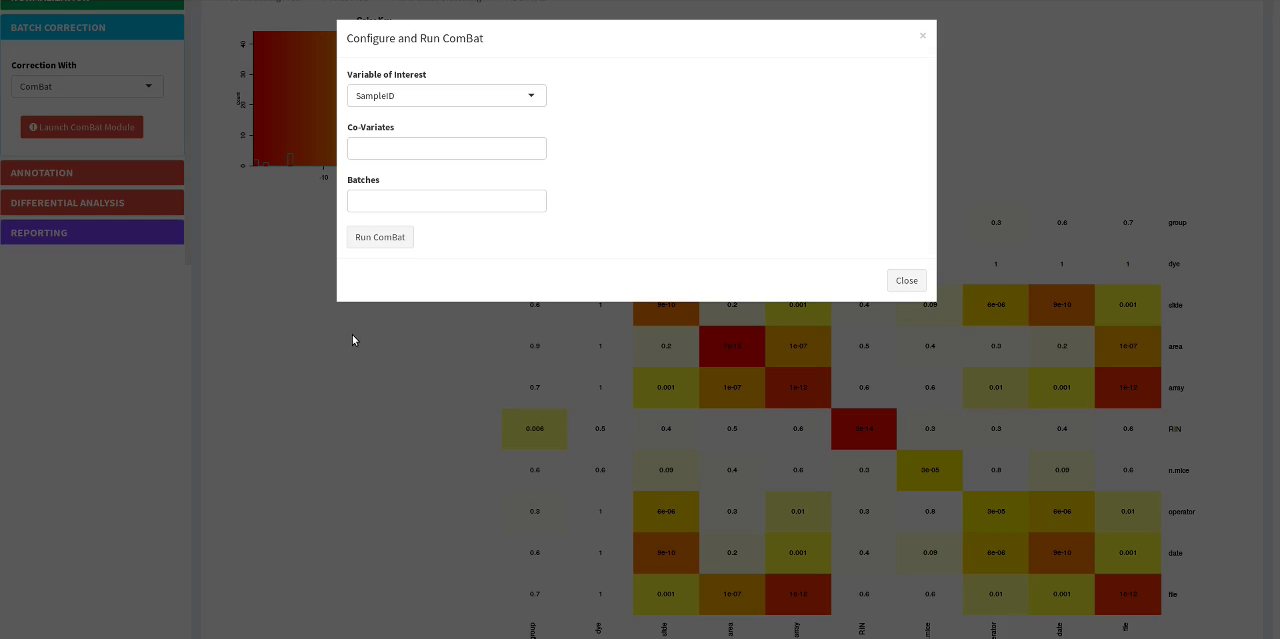
**

## ComBat Variable of Interest

**
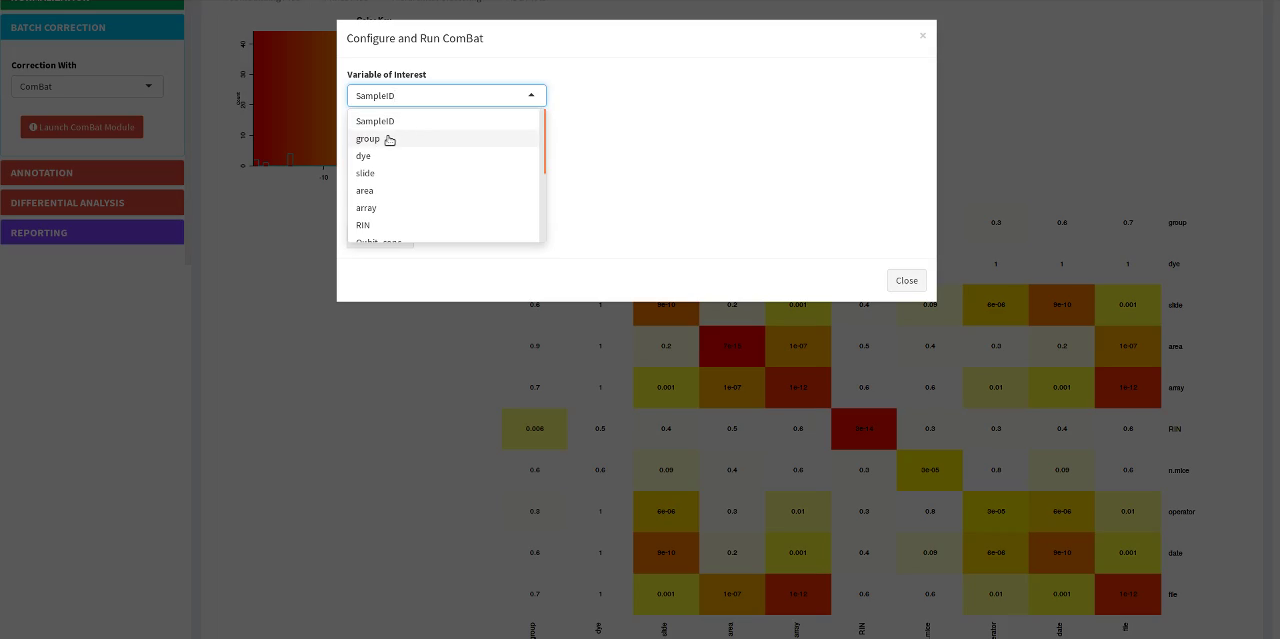
**

## ComBat Batches

**
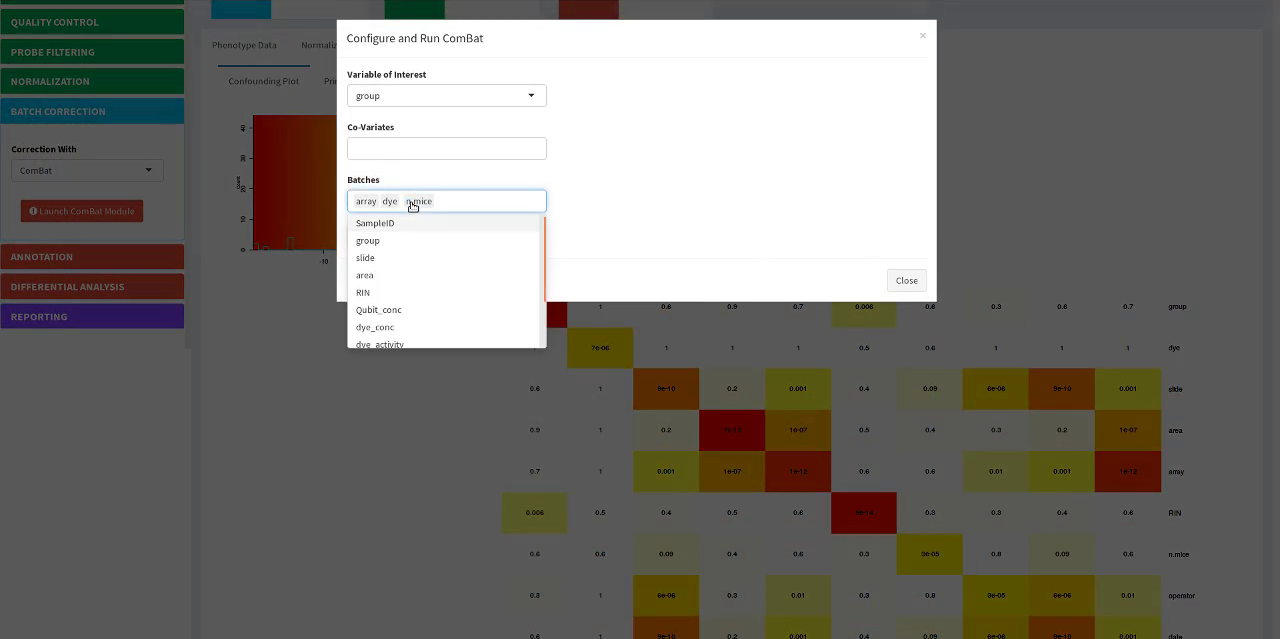
**

## Perform Batch Correction

**
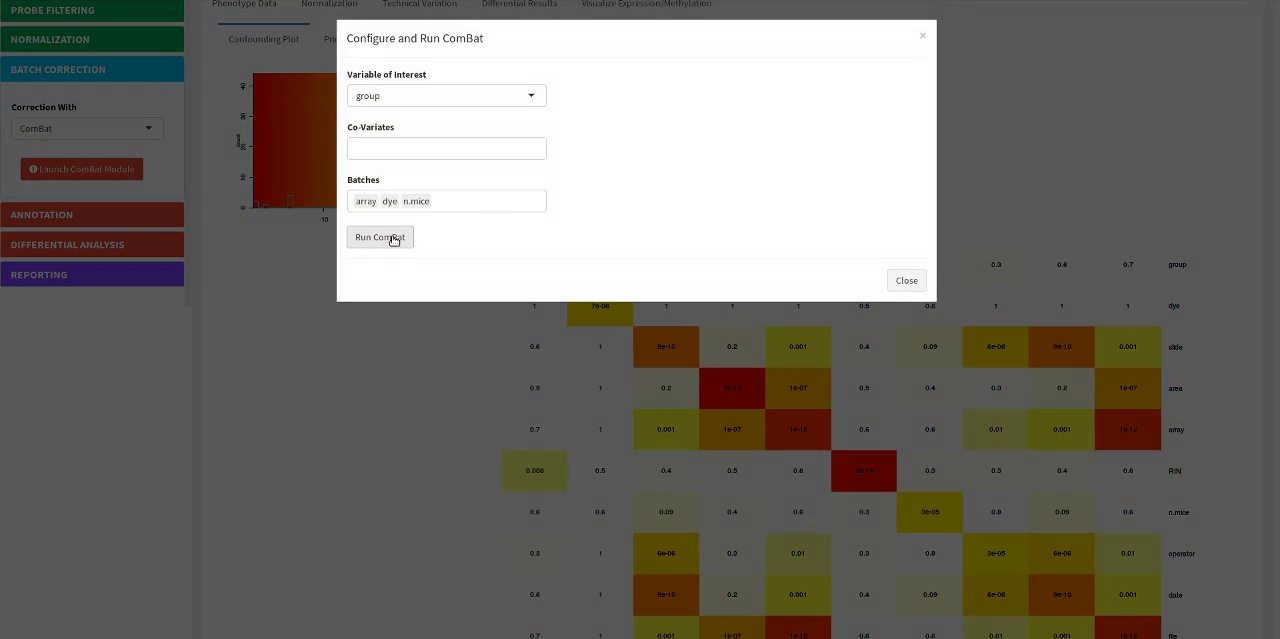
**

## Batch Correction in Progress

**
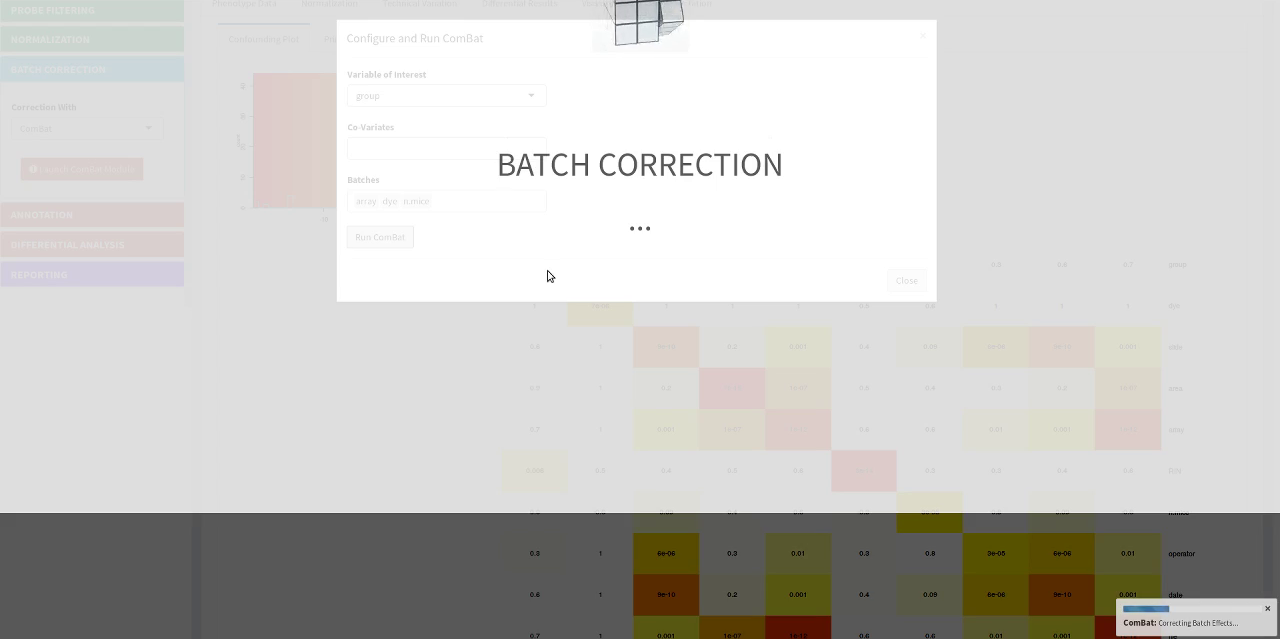
**

# Technical Variation After Known Correction

## MDS Plot

Multi-Dimensional Scaling plot displaying the distance between samples from the batch corrected data can be viewed from the *After Correction* tab nested within the sub-tab *MDS Plots* nested within the *Technical Variation* tab.

**
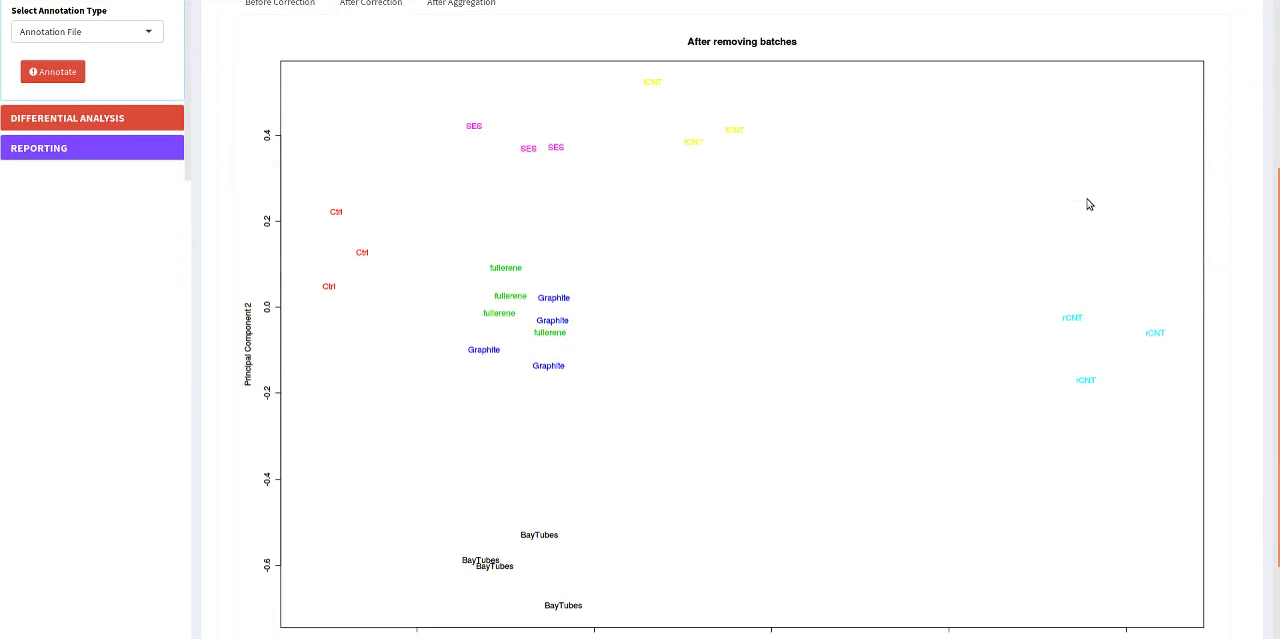
**

## Prince Plot

Prince plot displaying the association between the phenotype variables and the principal components from the batch corrected data can be viewed from the *After Correction* tab nested within the sub-tab *Prince Plot* nested within the *Technical Variation* tab.

**
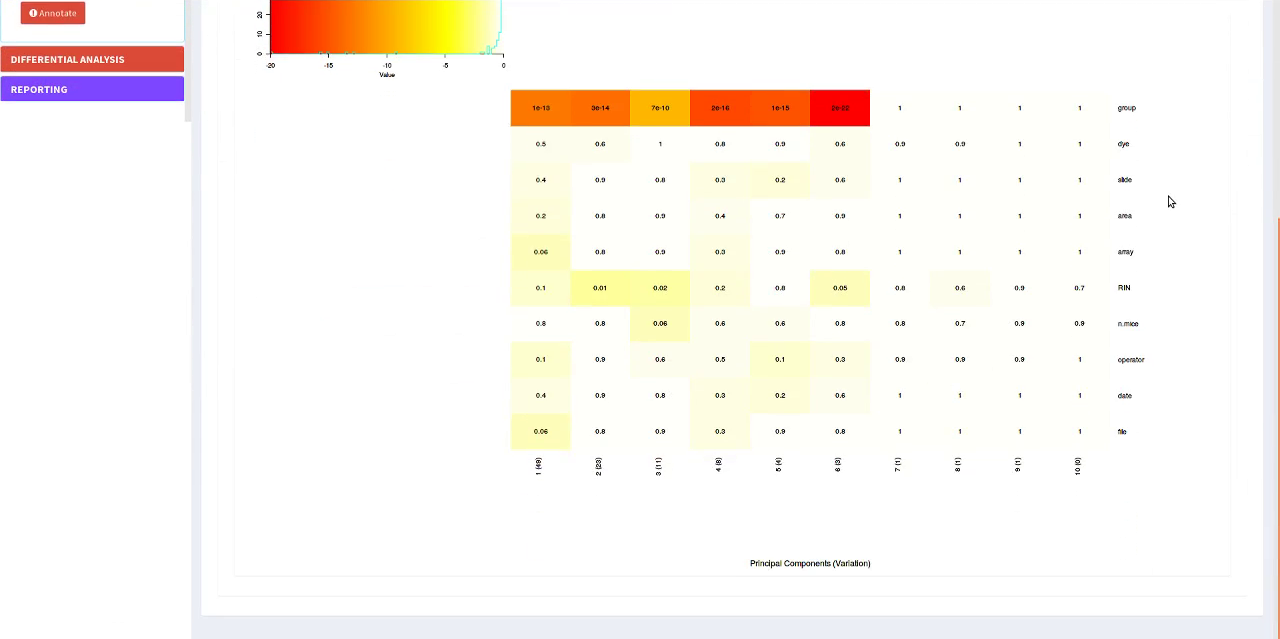
**

## Hierarchical Clustering Plot

Hierarchical clustering plot displaying the clustering over the batch corrected data can be viewed from the *After Correction* tab nested within the sub-tab *Hierarchical Clustering* nested within the *Technical Variation* tab.

**
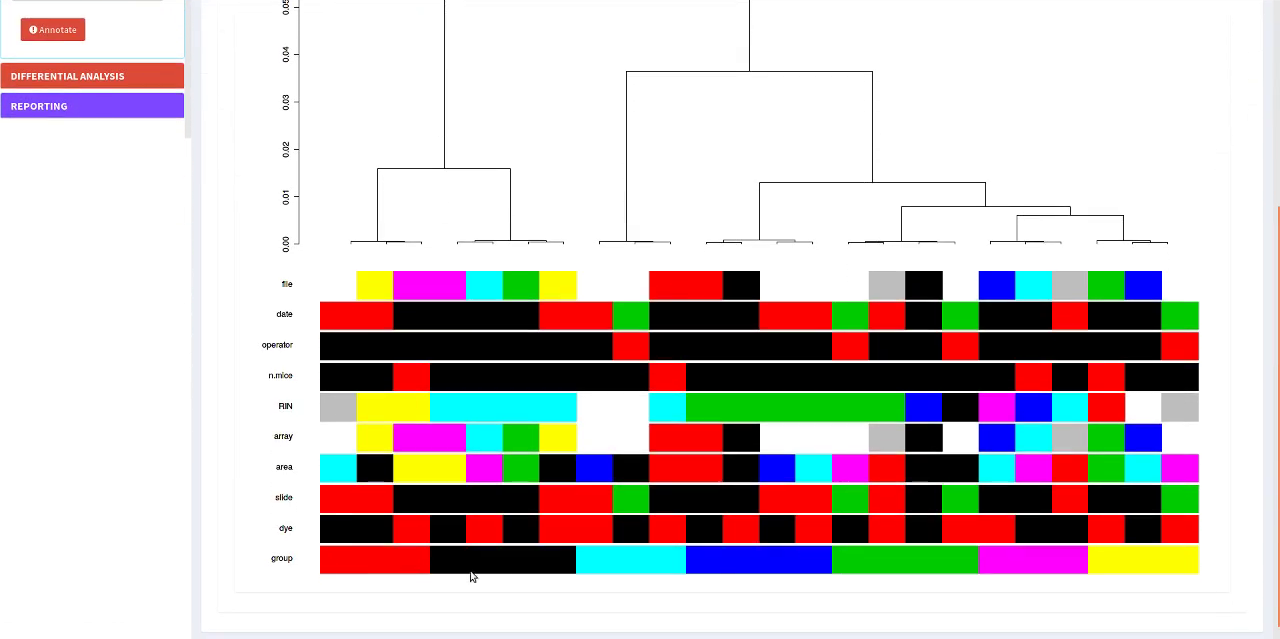
**

# Unknown Batch Correction

## Configure and Run SVA

In the SVA module window, user can specify the variable of interest and co-variates for identification of surrogate variables.


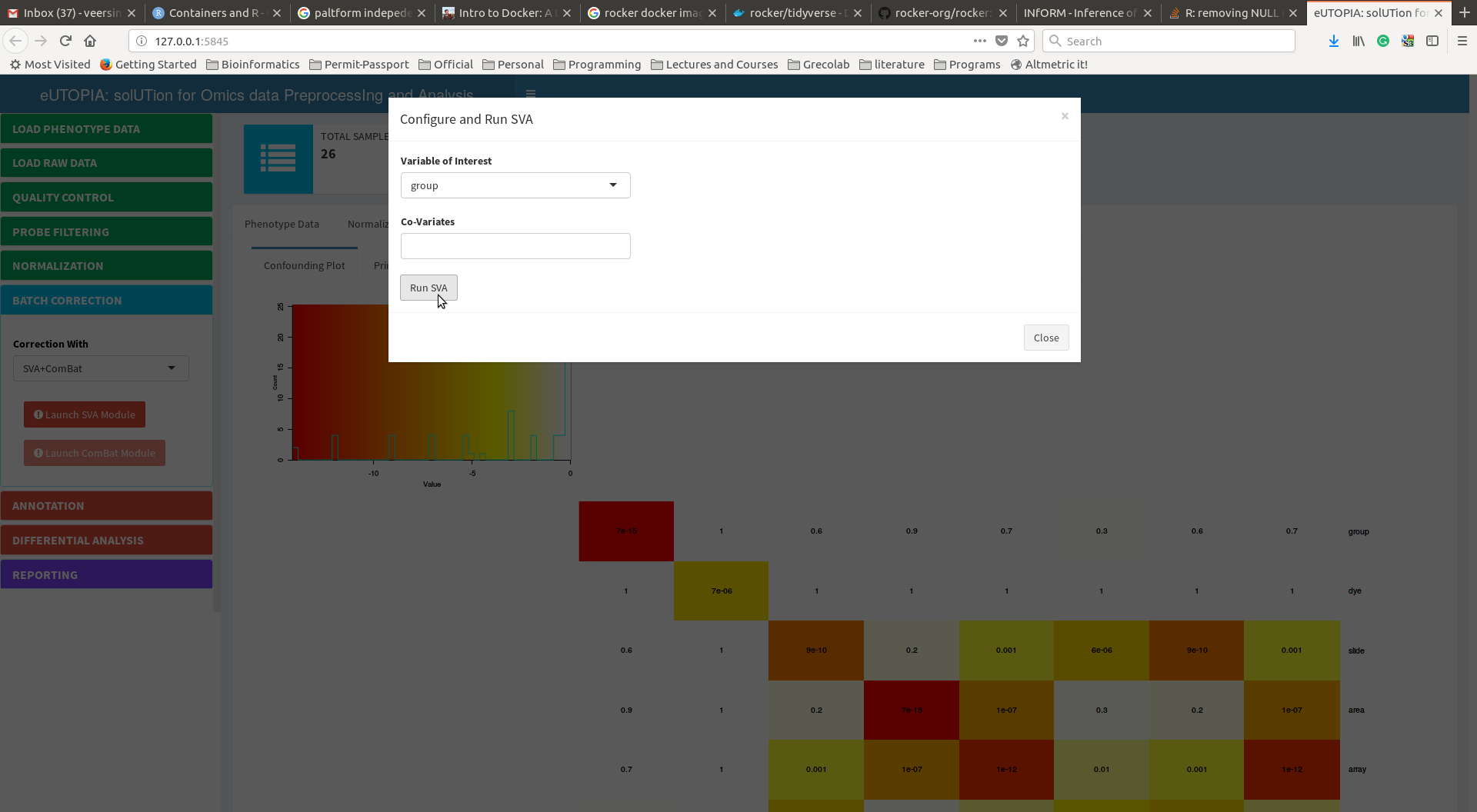


## Confounding Plot with SVA Variables

After completion of the SVA step, the *Confounding Plot* is updated with the identified surrogate variables to show the relatedness of the surrogate variables as well.

**
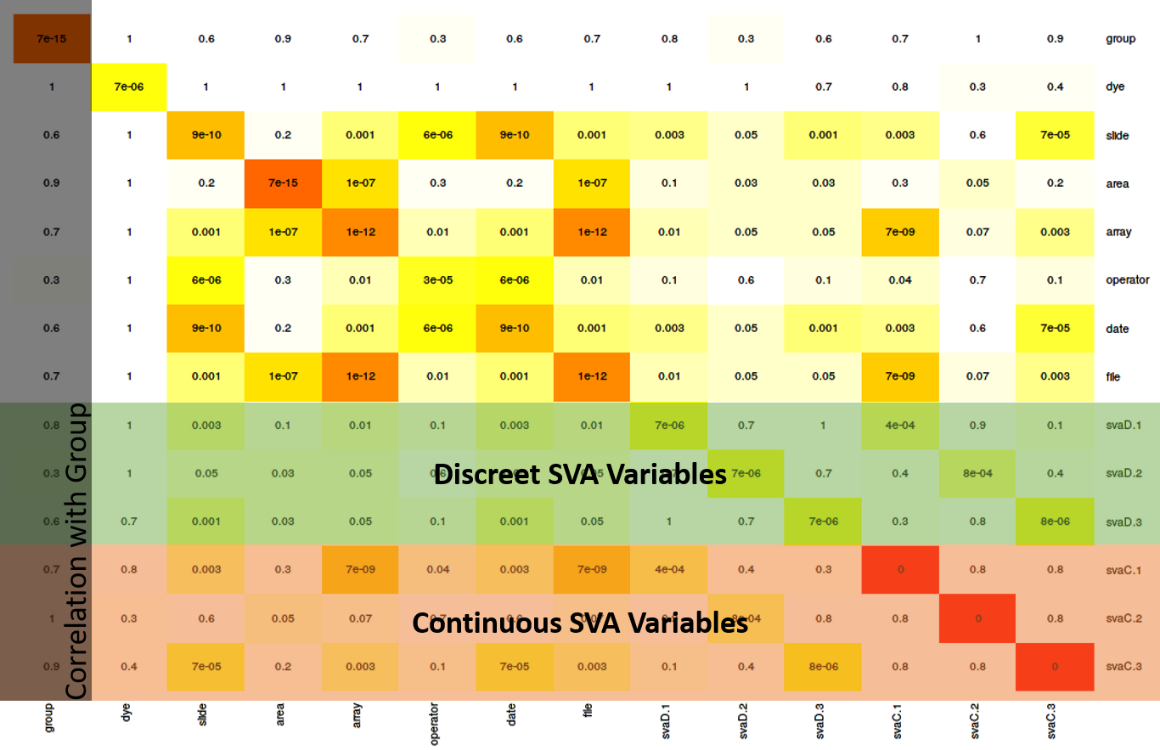
**

## Prince Plot with SVA Variables

After completion of the SVA step, the prince plot is updated with the identified surrogate variables to show their association with the principal components.

**
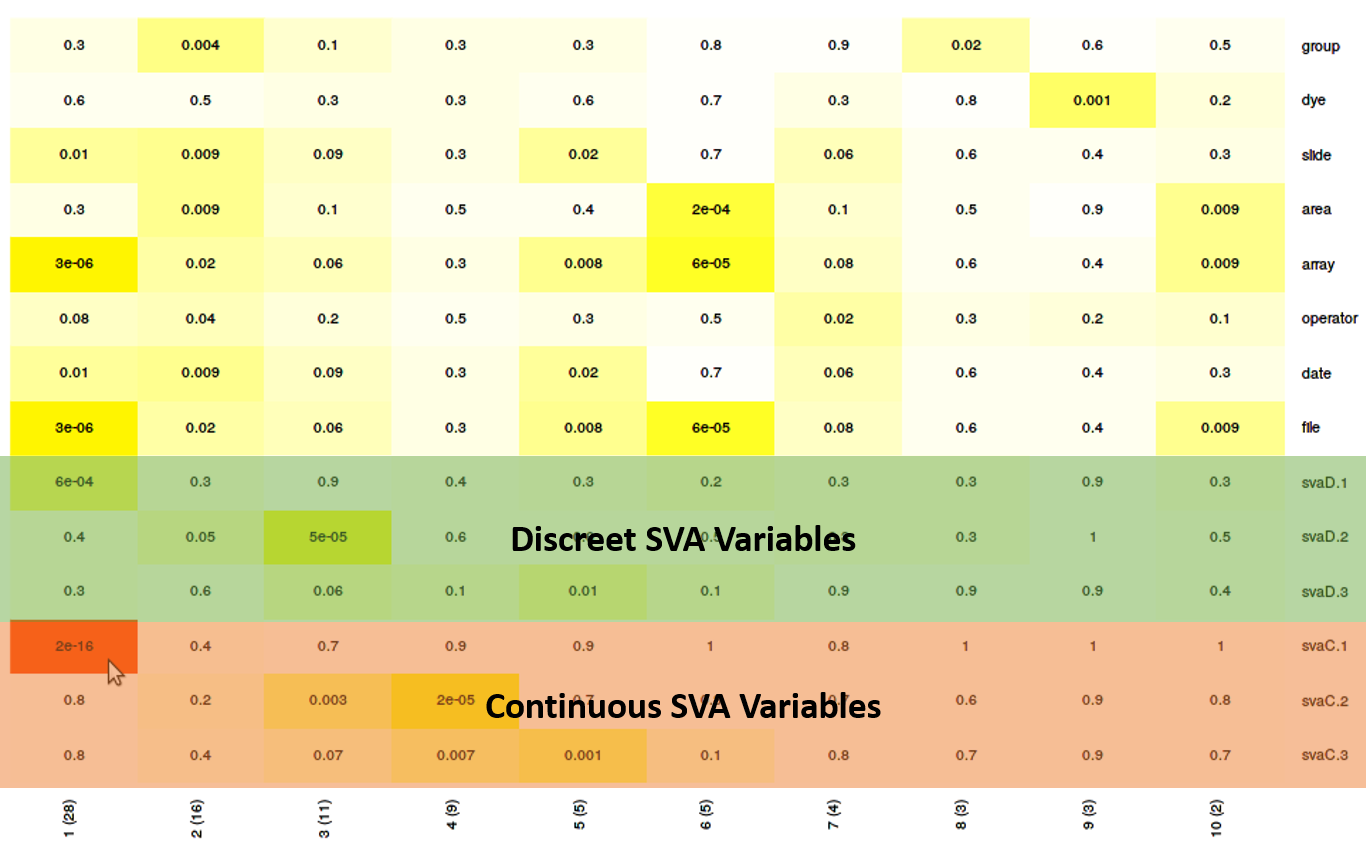
**

## Batch Correction with SVA Variables

ComBat module window display after SVA restricts the *Batches* option to discreet surrogate variables only, the *Variable of Interest* and *Co-Variates* options use the known variables.

**
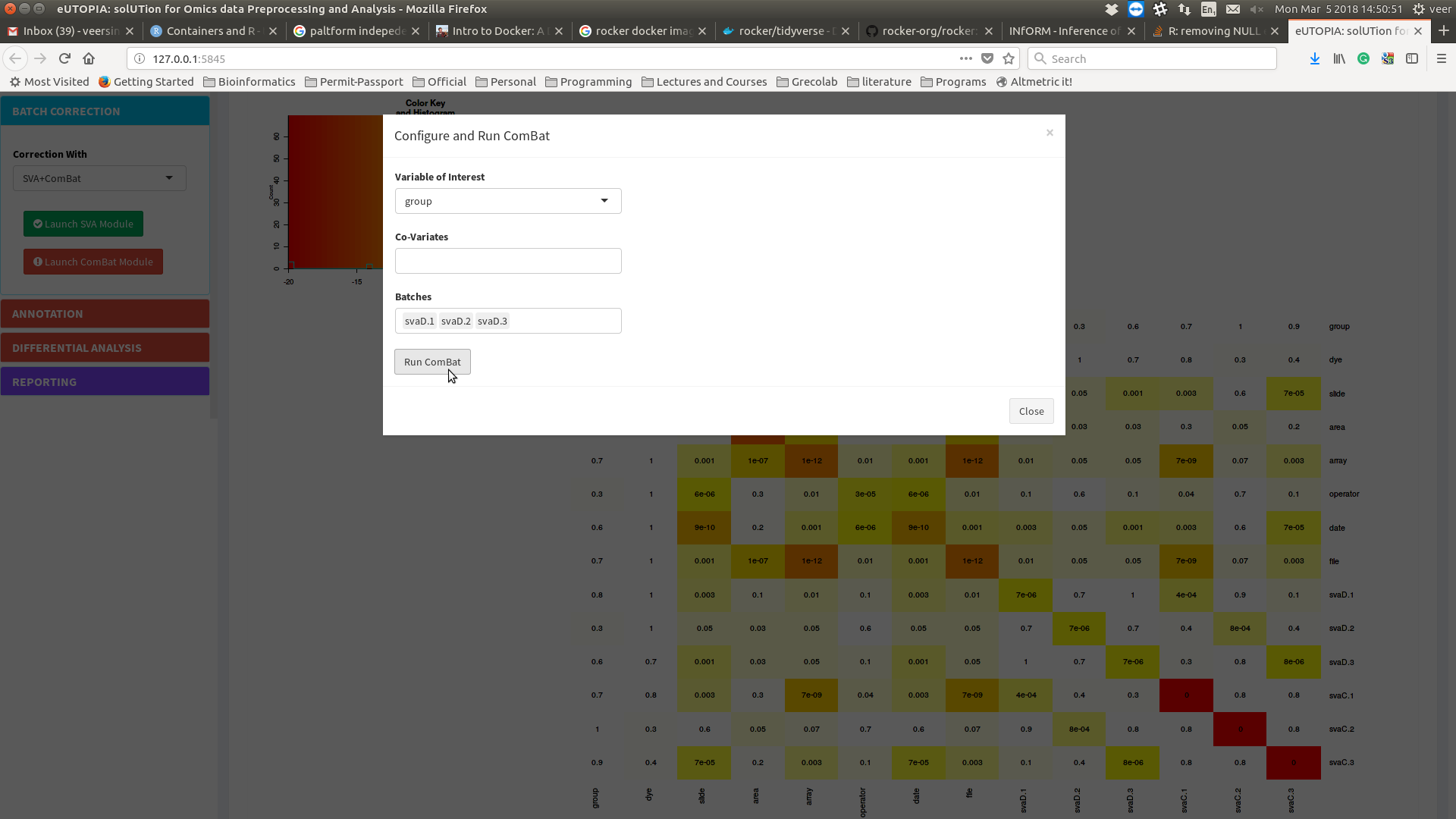
**

## Batch Correction in Progress

**
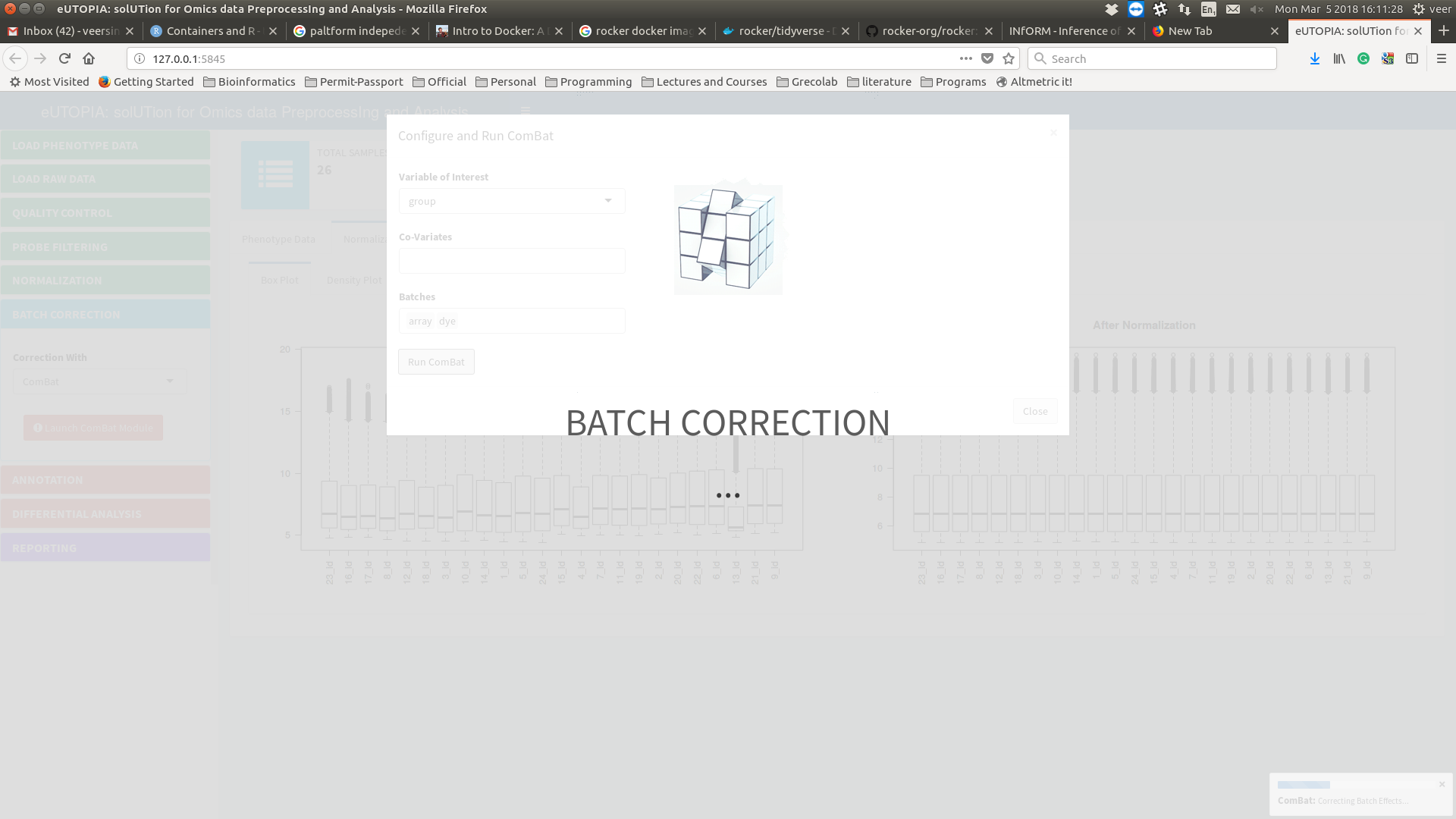
**

# Technical Variation After Unknown Correction

## Prince Plot

Prince plot after batch correction by using surrogate variables.

**
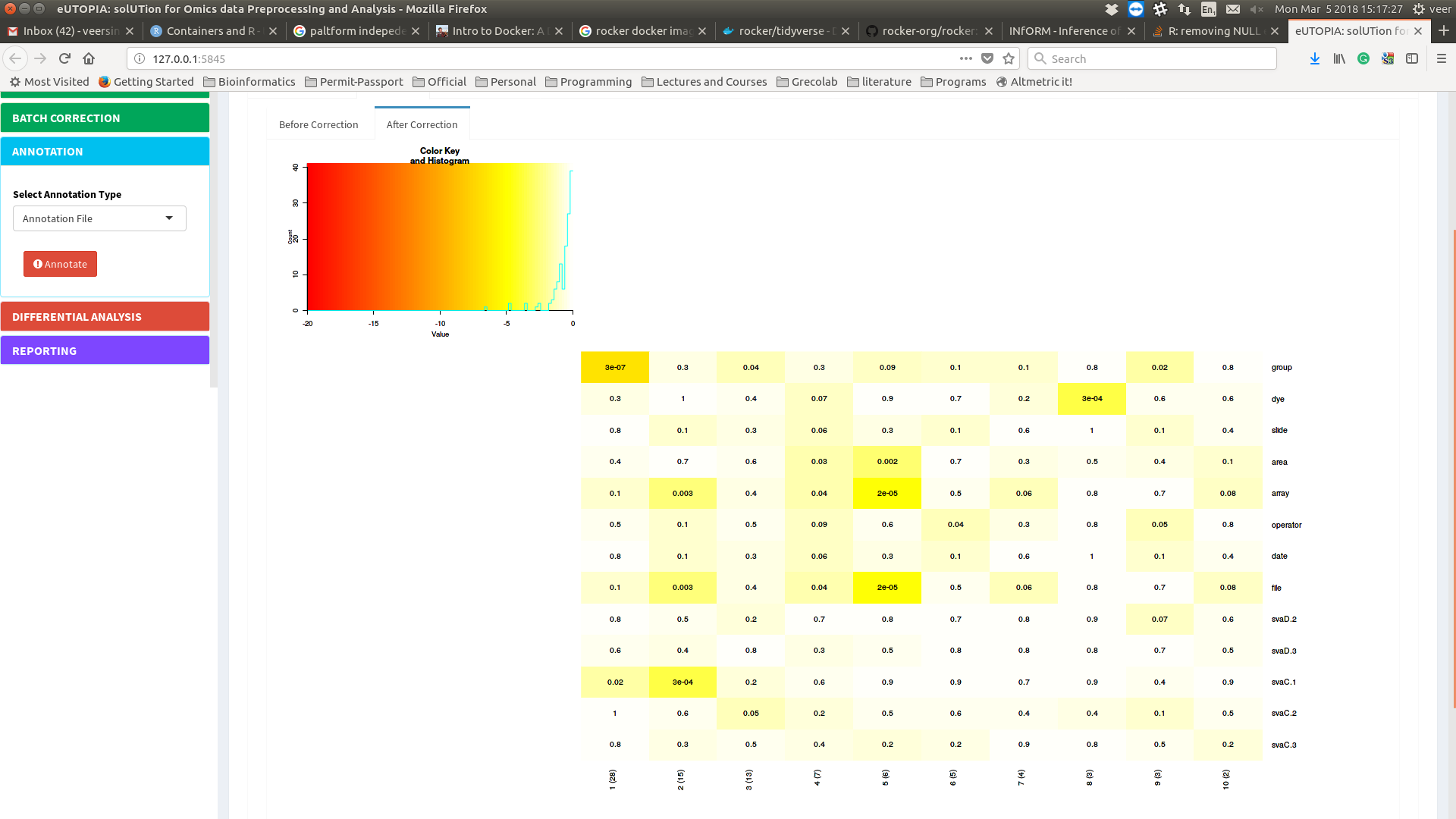
**

# Annotation

## Annotation Options

The user can provide array specific annotation file for the Agilent platforms, alternately intrinsic annotation of the array can be obtained from the raw data. Illumina methylation platform takes intrinsic array annotation by default and CDF annotations are used for Affymetrix expression arrays which are provided while uploading raw data (not shown here).

| Available Options | Use Annotation from Raw Data | Upload Annotation File |
| --- | --- | --- |
| **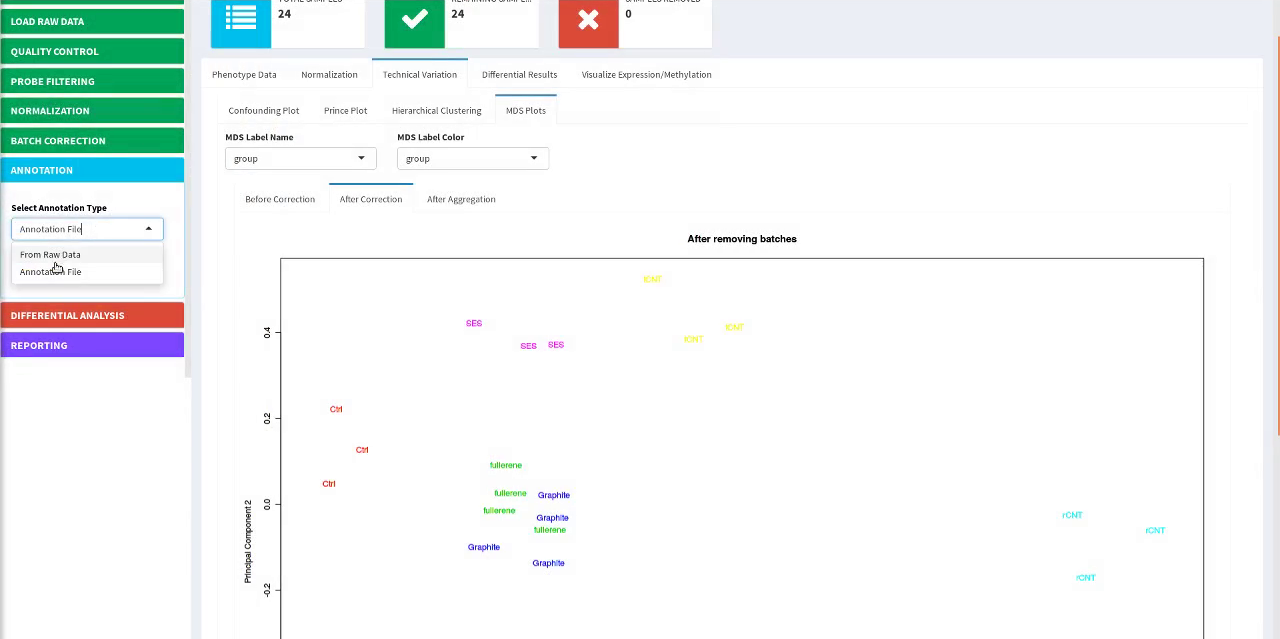** | **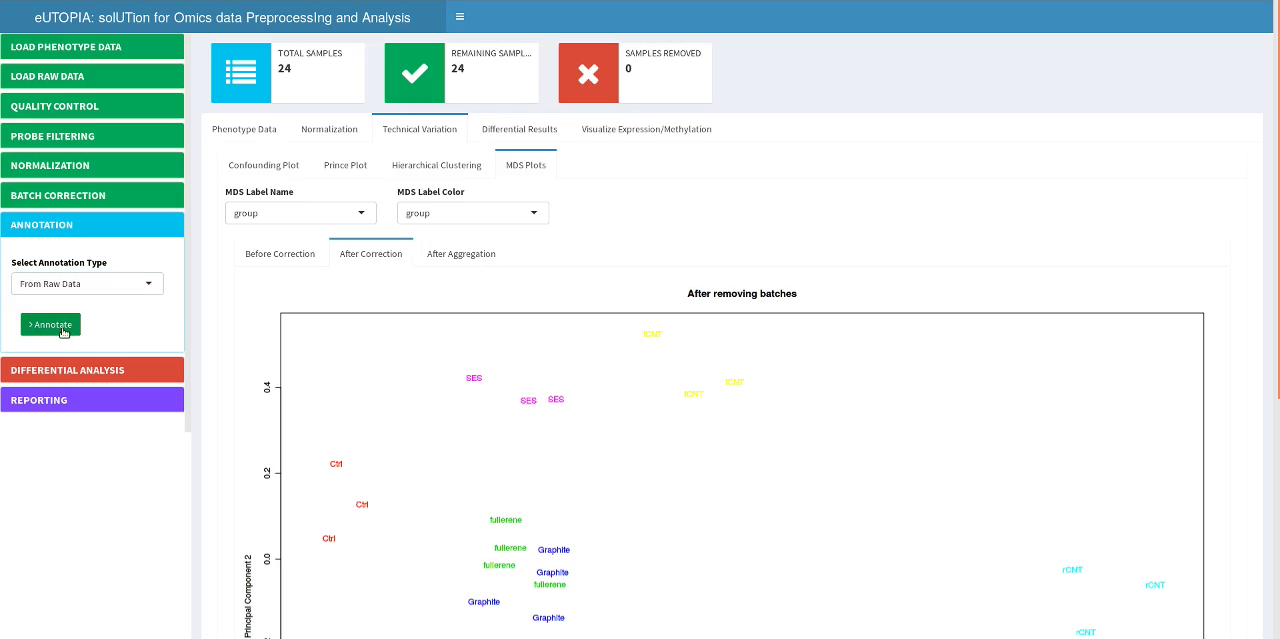** | **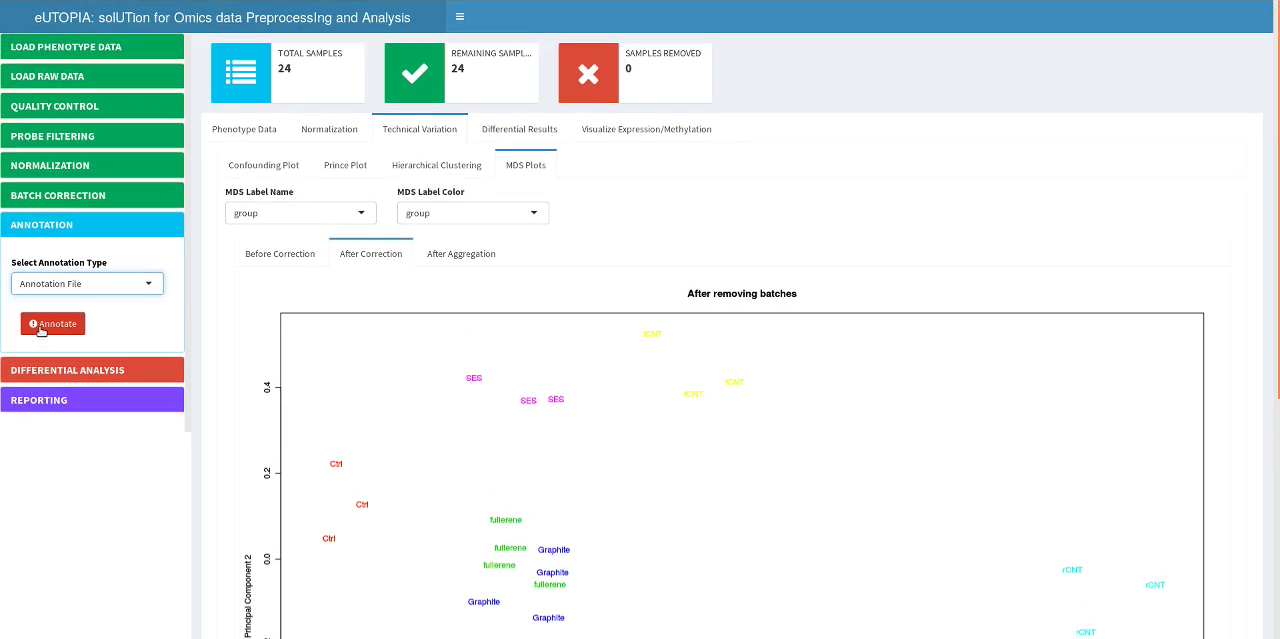** |

## Select Annotation File

*Annotation File* option from the *Select Annotation Type* input launches *Input Annotation* window that lets the user configure and import the annotation file.

**
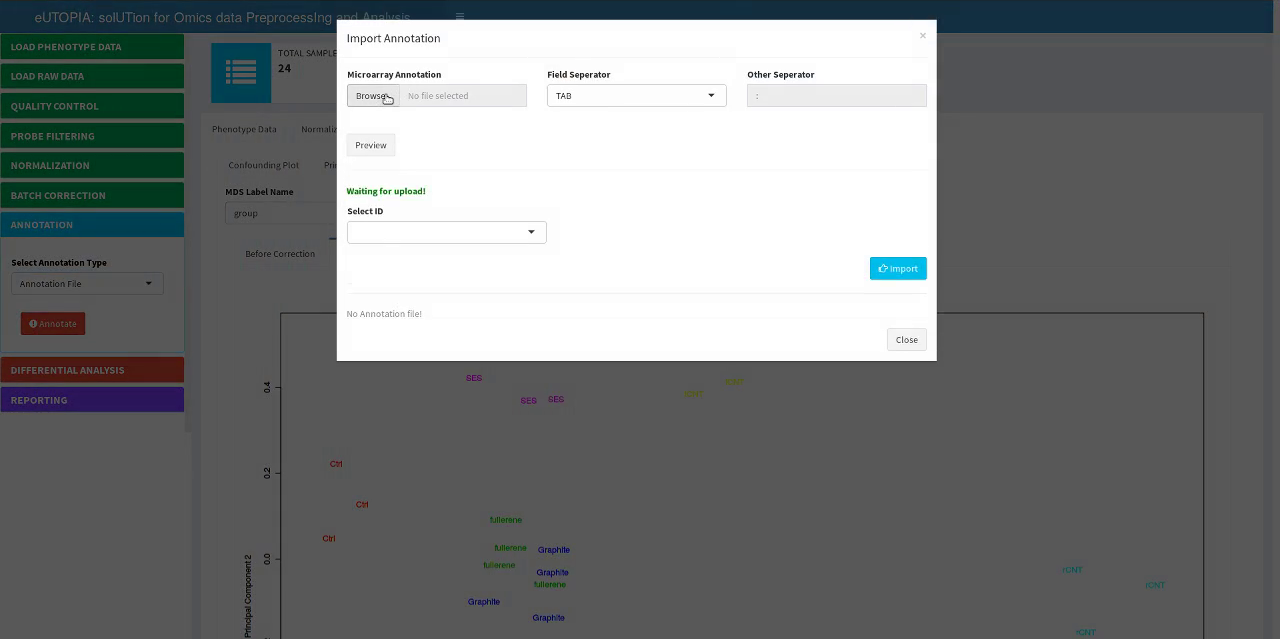
**

## Preview and Import

**
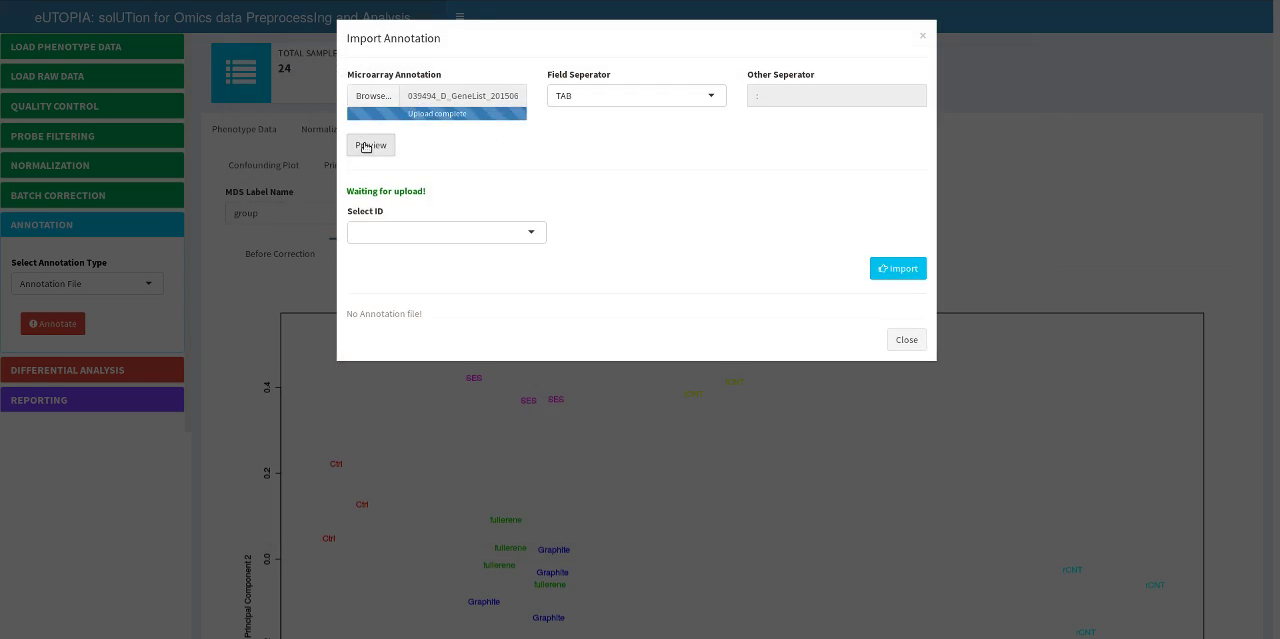
**

## Annotation in Progress

**
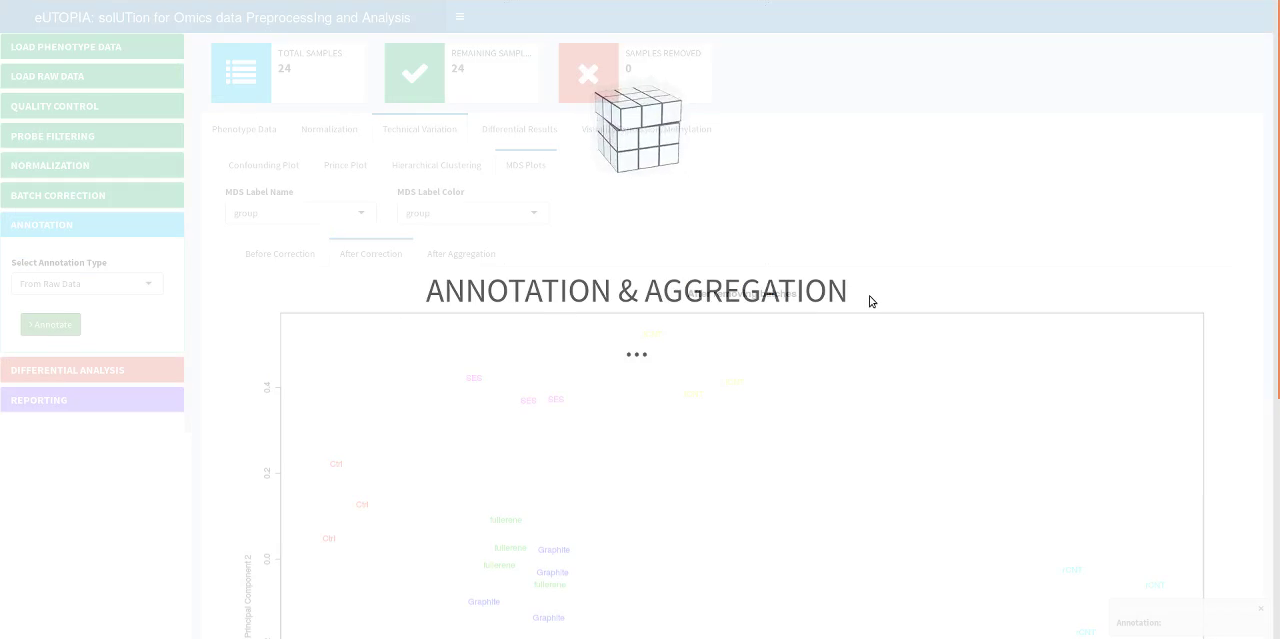
**

## MDS Plot After Aggregation

Multi-Dimensional Scaling plot displaying the distance between samples from the data aggregated by annotation can be viewed from the *After Aggregation* tab nested within the sub-tab *MDS Plots* nested within the *Technical Variation* tab.

**
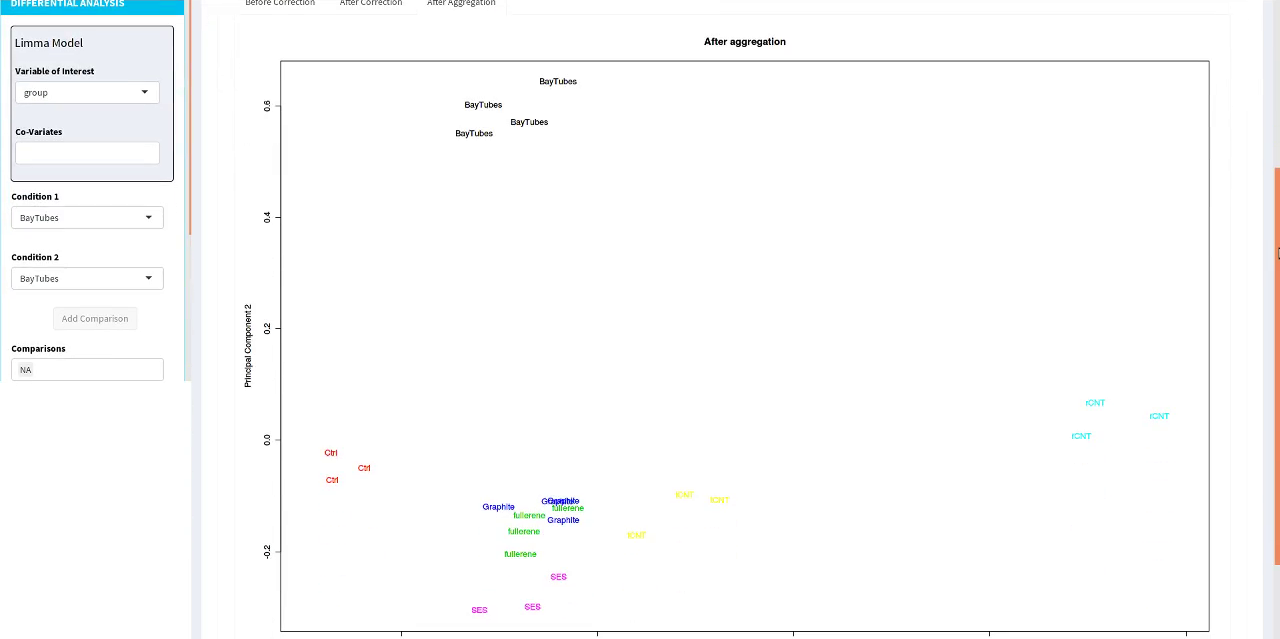
**

# Differential Analysis

## Configure Limma Model

*Differential Analysis* step has *Limma Model* configuration parameters *Variable of Interest* and *Co-Variates*. Limma contrasts can be created by specifying *Condition 1* and *Condition 2* followed by *Add Comparison*. The user can remove any excess contrasts from the *Comparison* input. P-value adjustment methods from Limma are provided as options in *P.value Adjustment Method* input control.

| Variable of Interest | Covariates | SVA Strategy: Covariates | Select Conditions |
| --- | --- | --- | --- |
| **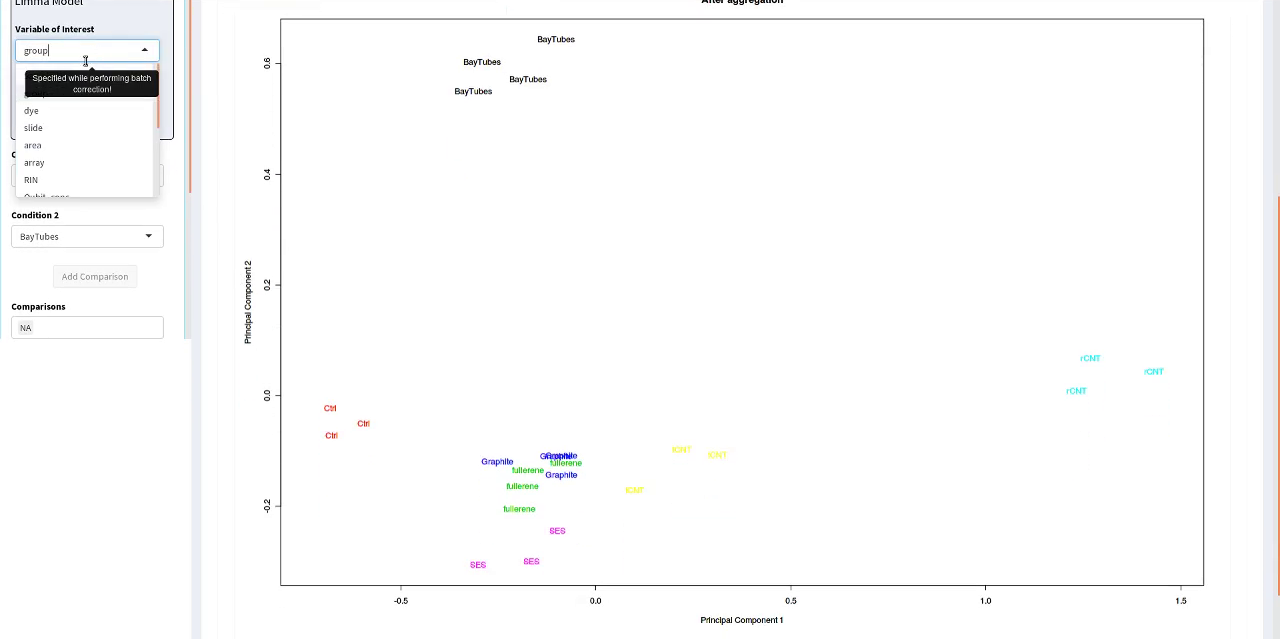** | **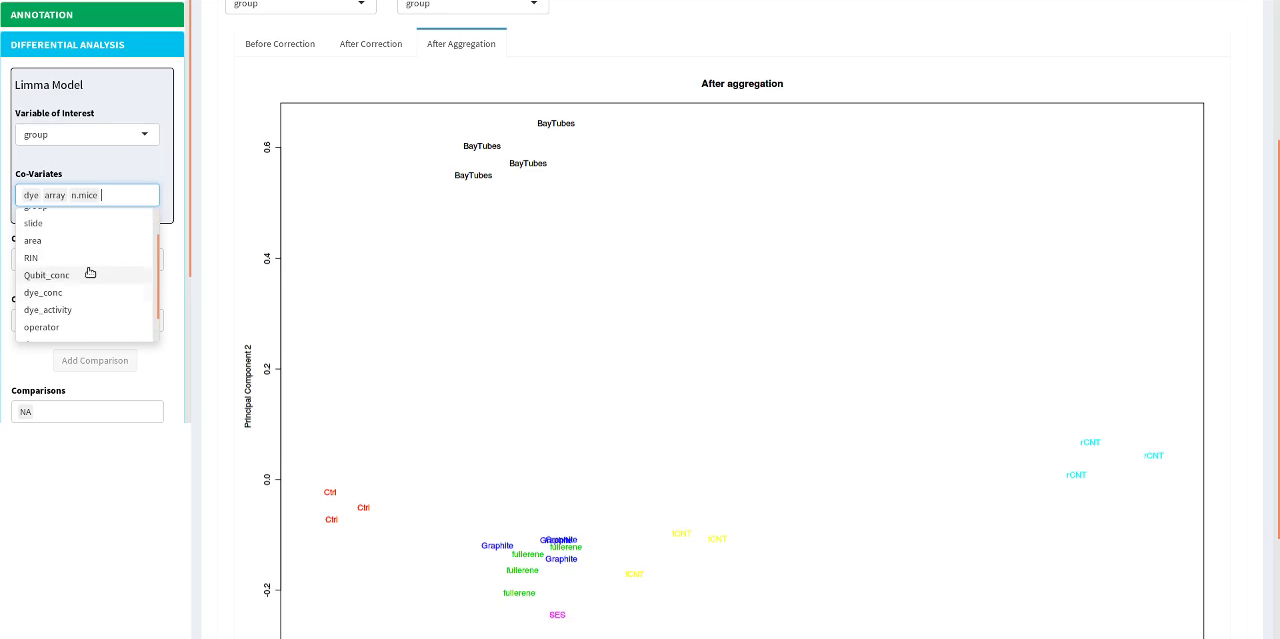** | **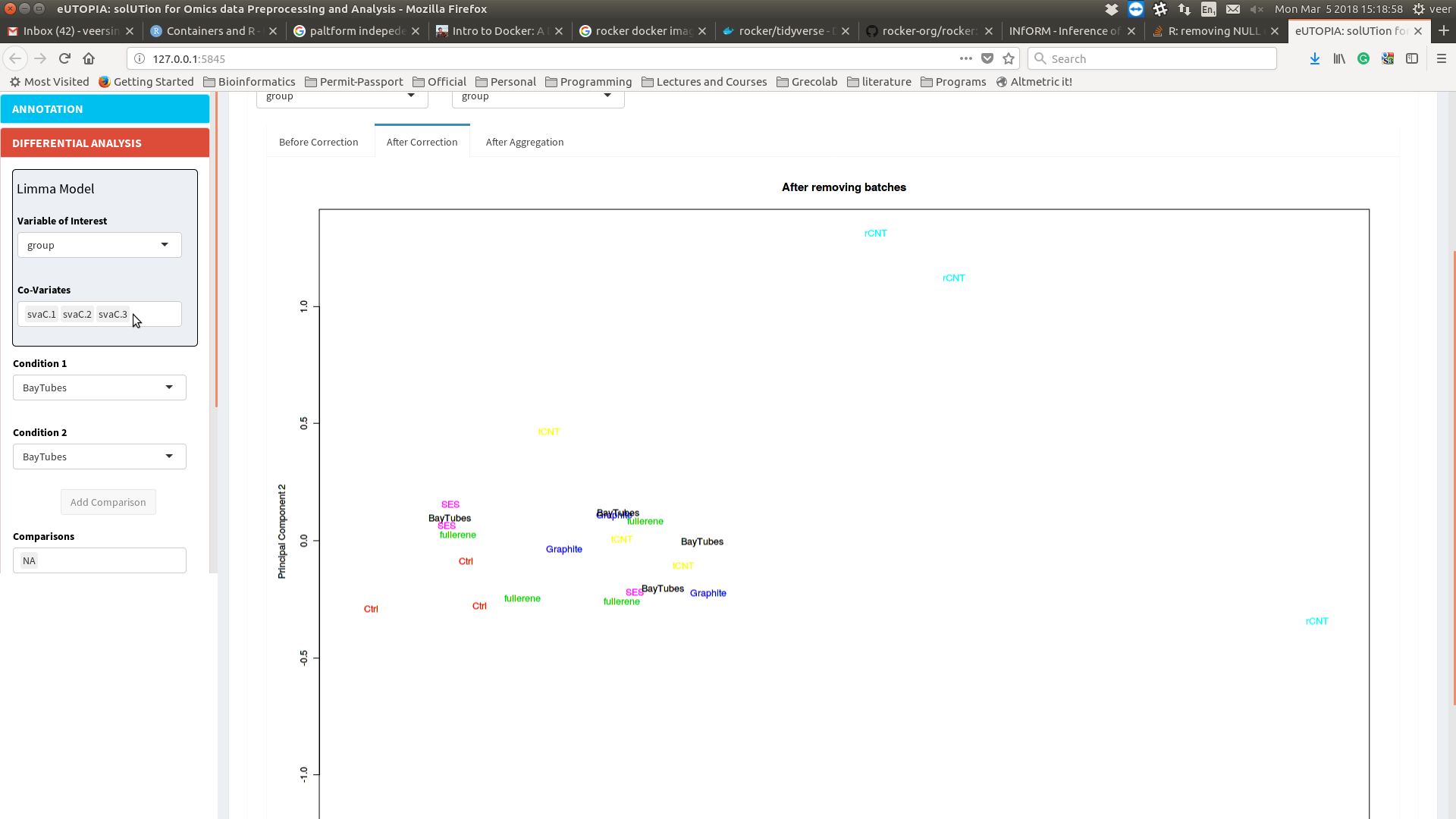** | **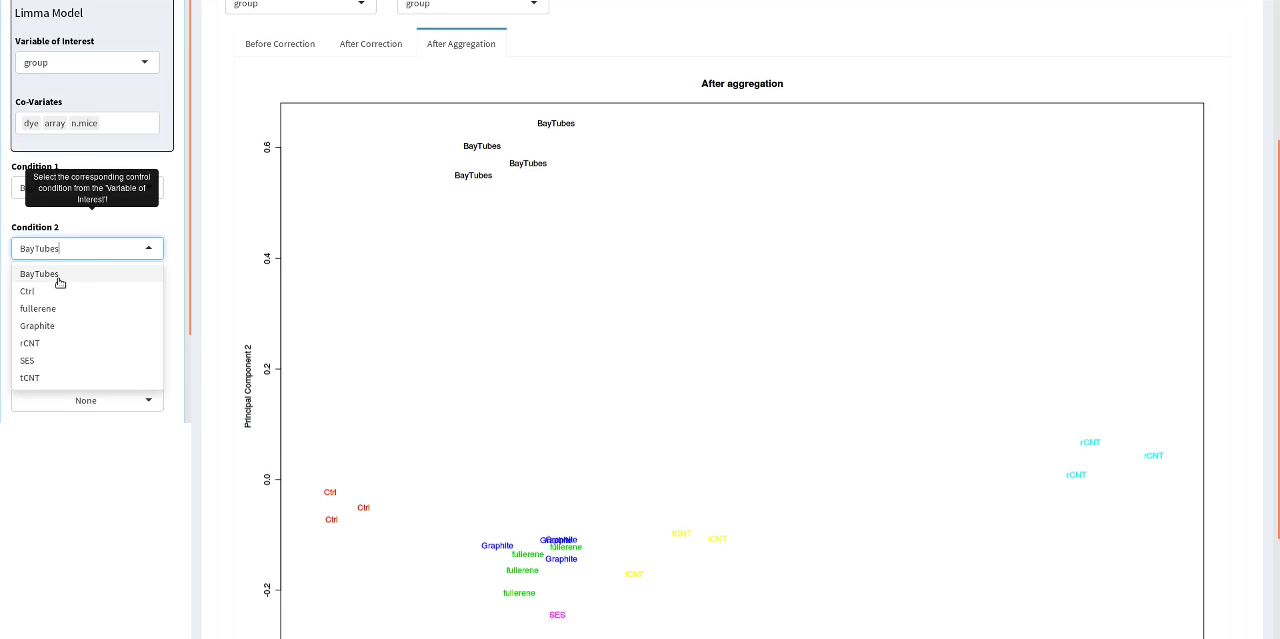** |

| Create Comparison | Add/Remove Created Comparison | Specify PValue Adjustement Method |
| --- | --- | --- |
| **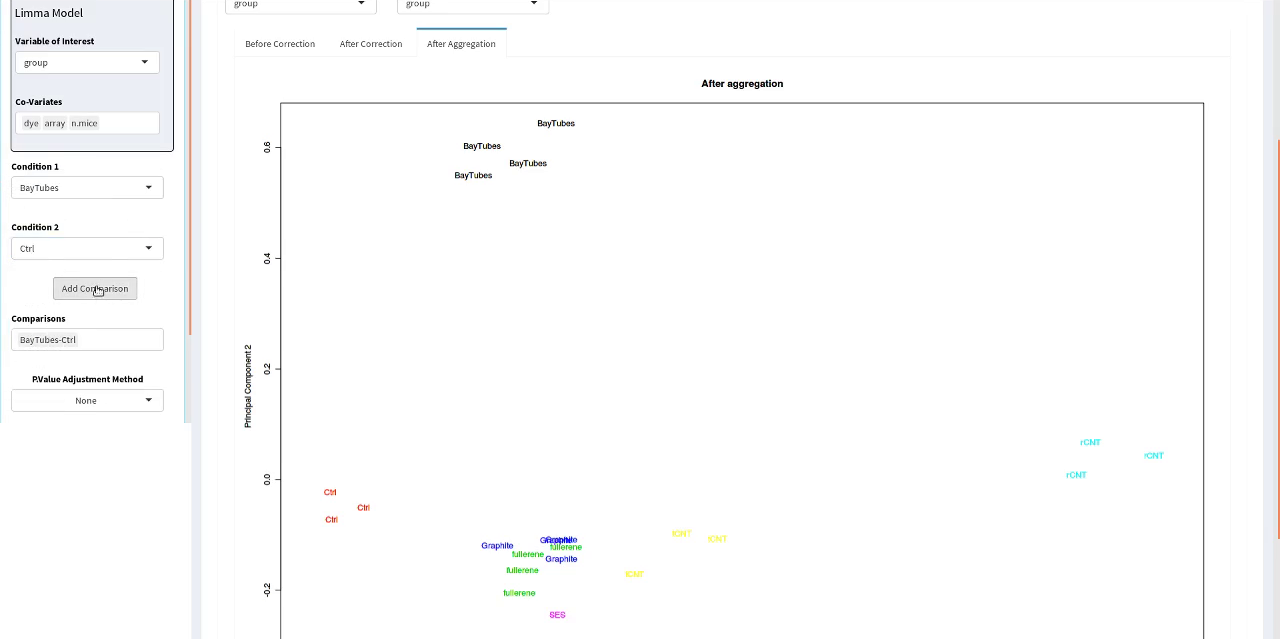** | **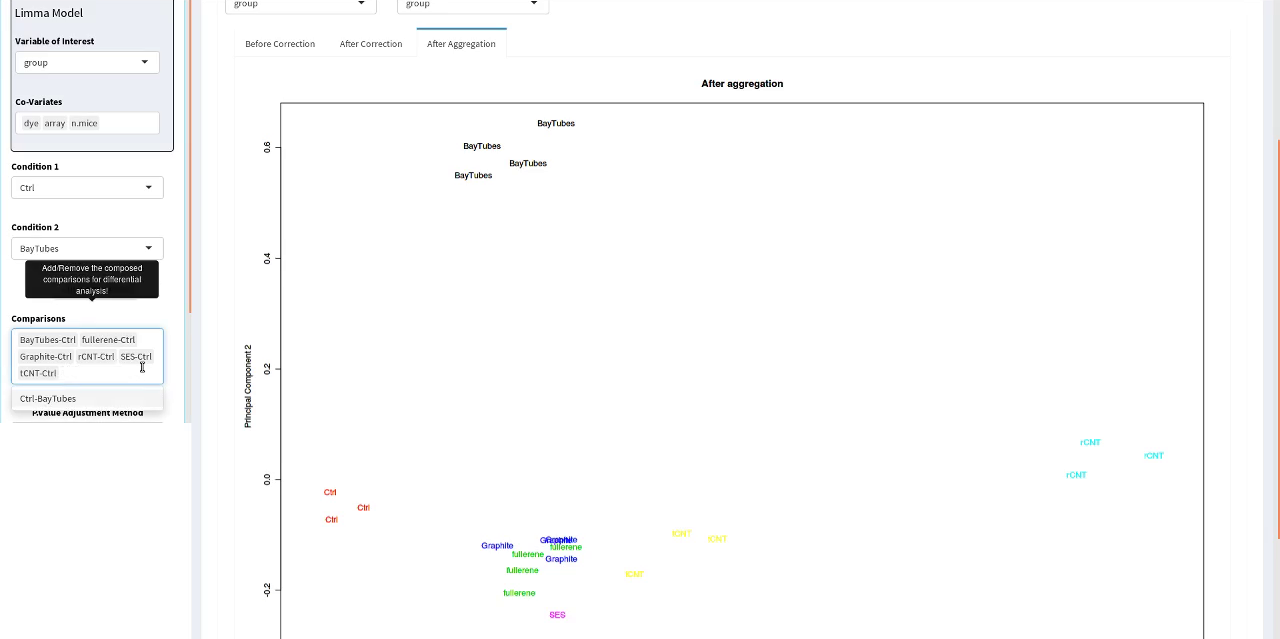** | **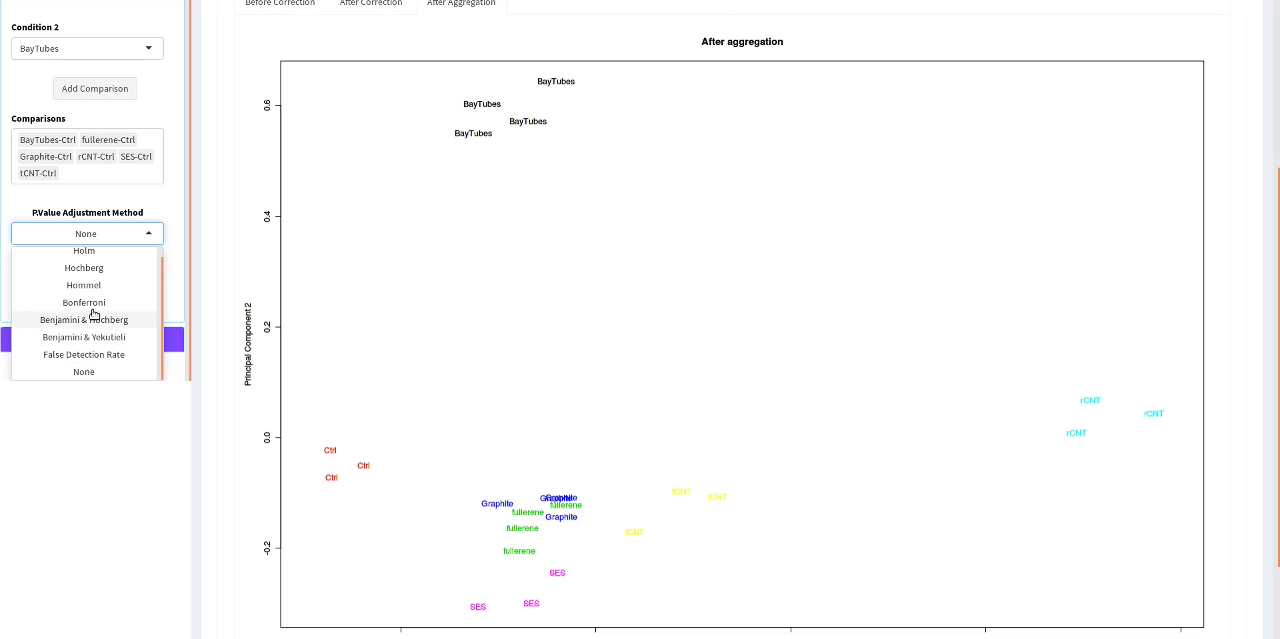** |

## Perform Differential Analysis

**
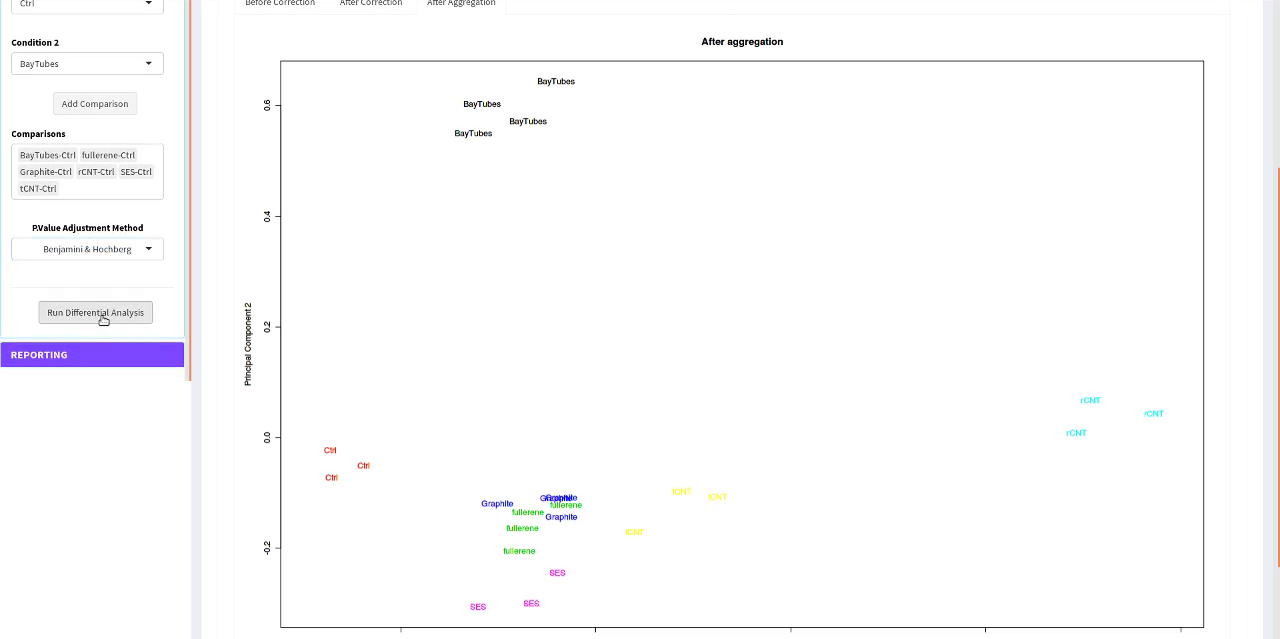
**

## Differential Analysis in Progress

**
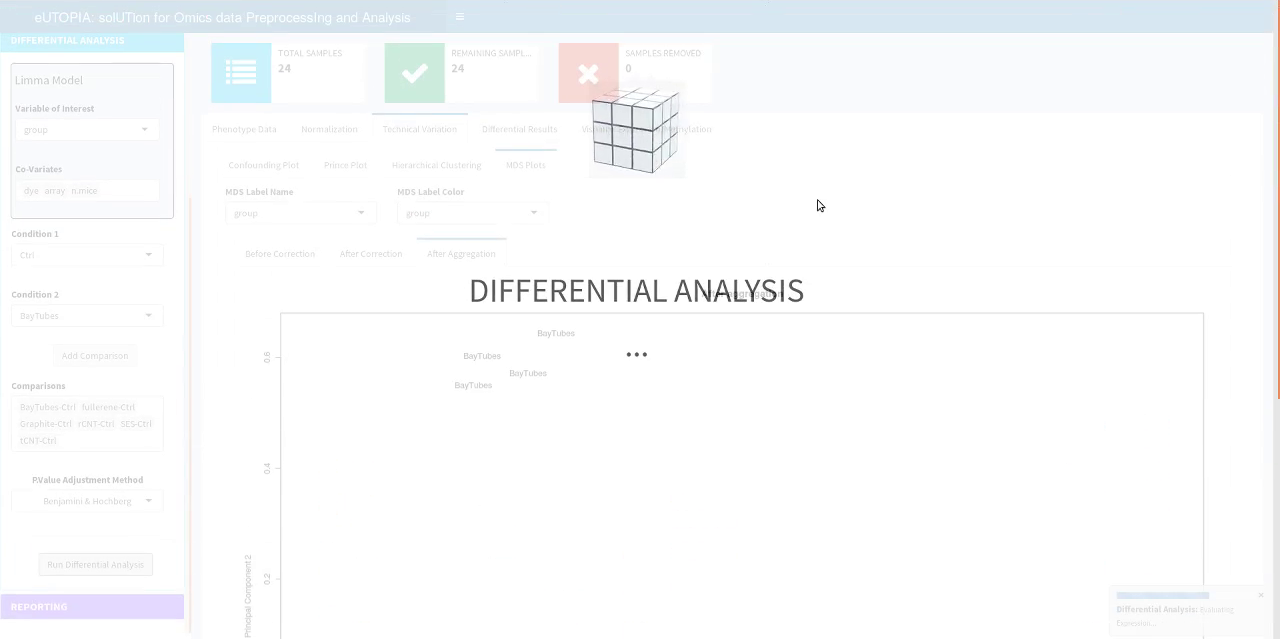
**

# Differential Results

Results of the differential analysis are displayed in the *Differential Results* tab in the main display area.

**
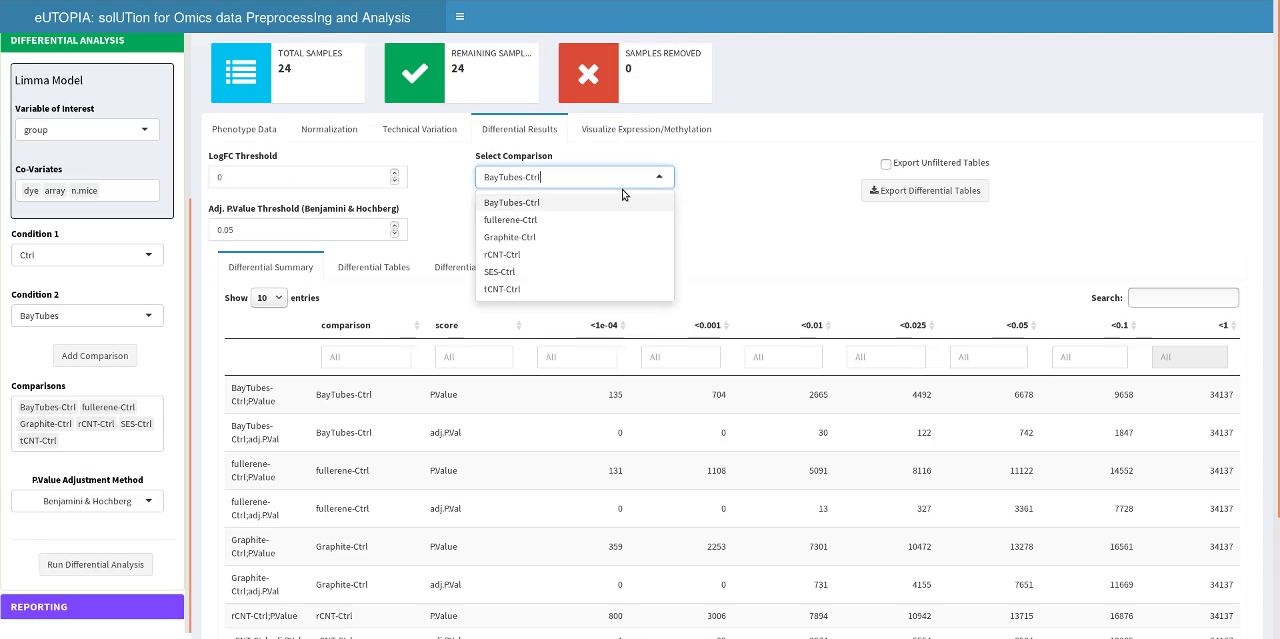
**

## Differential Results Summary

*Differential Summary* sub-tab displays the summary of the differential features by different thresholds of P.Value and adj.PVal. This summary table is updated on changing logFC threshold.

**
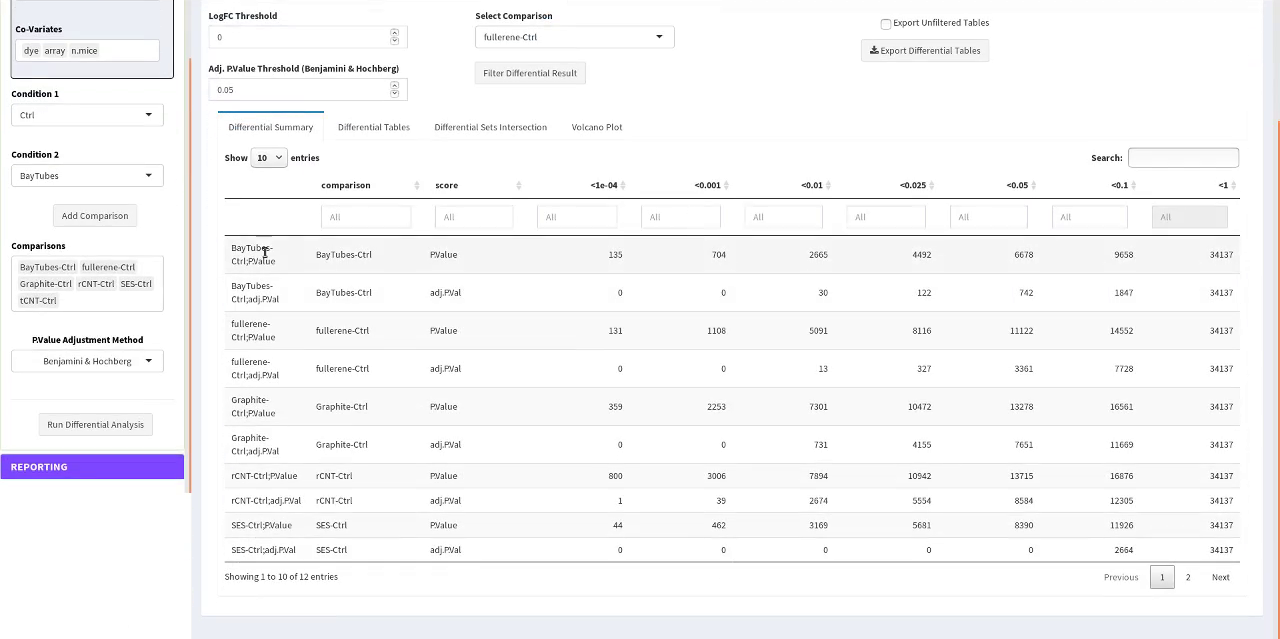
**

## Differential Results Table

*Differential Tables* sub-tab displays the table of the differential features obtained by Limma differential analysis. It shows results filtered by logFC and P.value thresholds for the specific comparison.

**
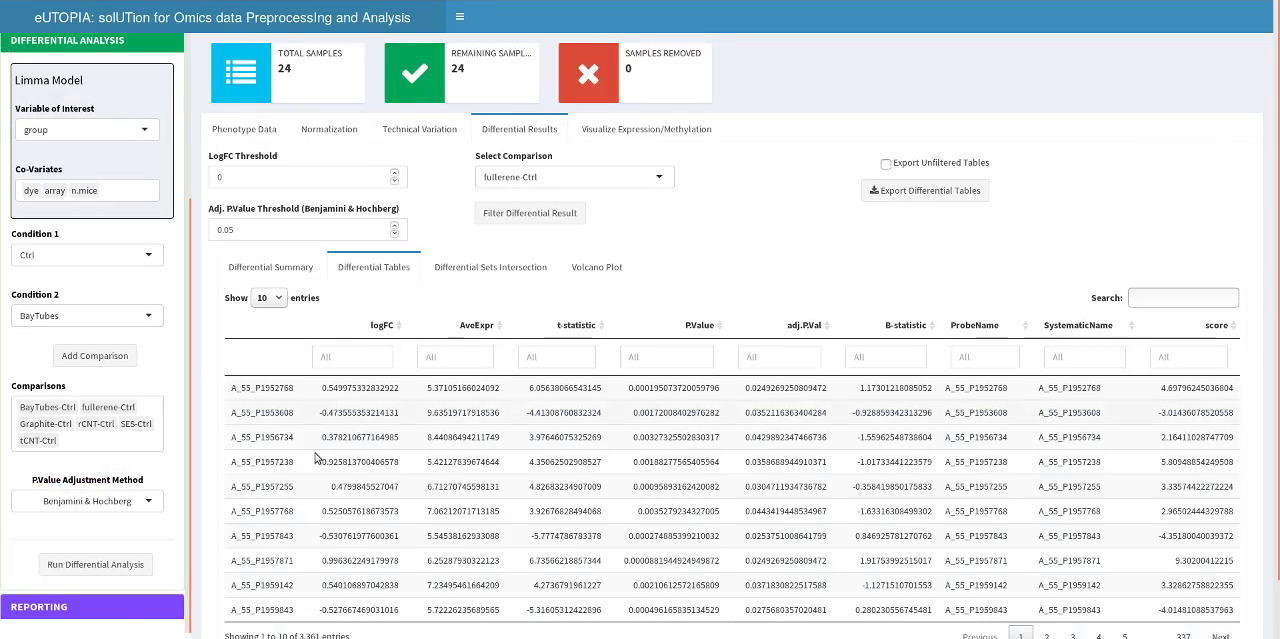
**

## Filter and Export Differential Tables

*Differential Results* can be filtered by using the *LogFC Threshold*, *Adj.P.Value Threshold*, and *Select Comparison* parameters. Differential results can be exported as single combined spreadsheet by clicking on *Export Differential Tables* button; the user can also choose the *Export Unfiltered Tables* option to export tables without filtering by logFC and Adj.P.Value.

| Filter Differential Results | Export Differential Tables | Save Differential Tables as XLS |
| --- | --- | --- |
| **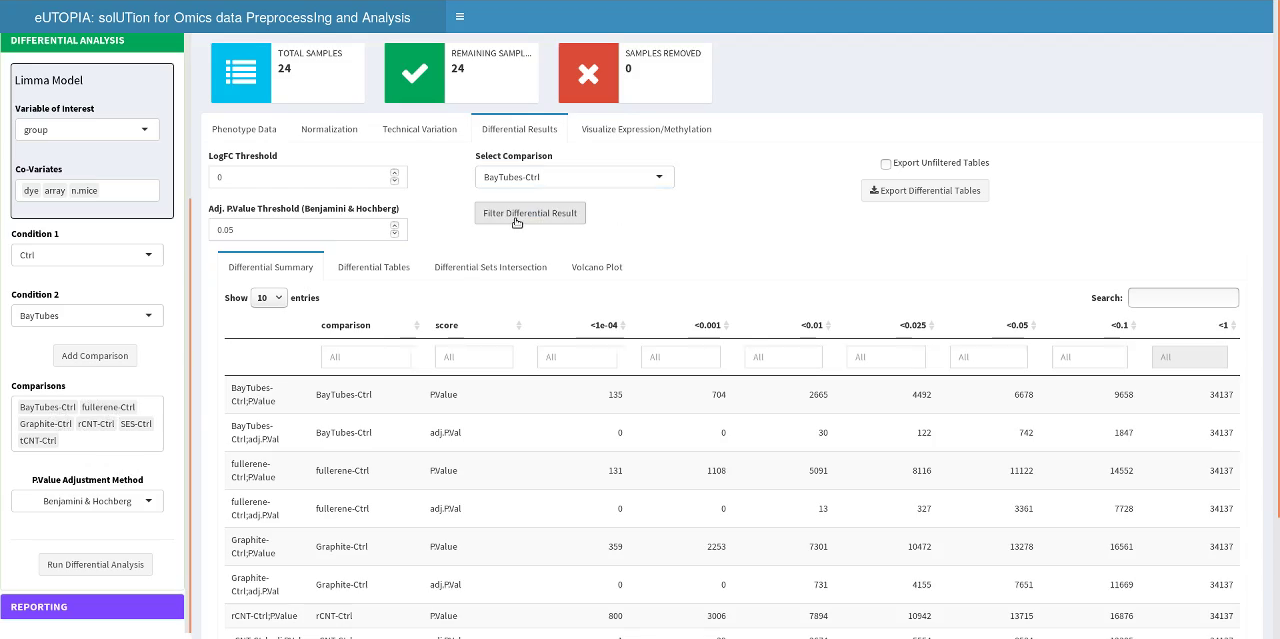** | **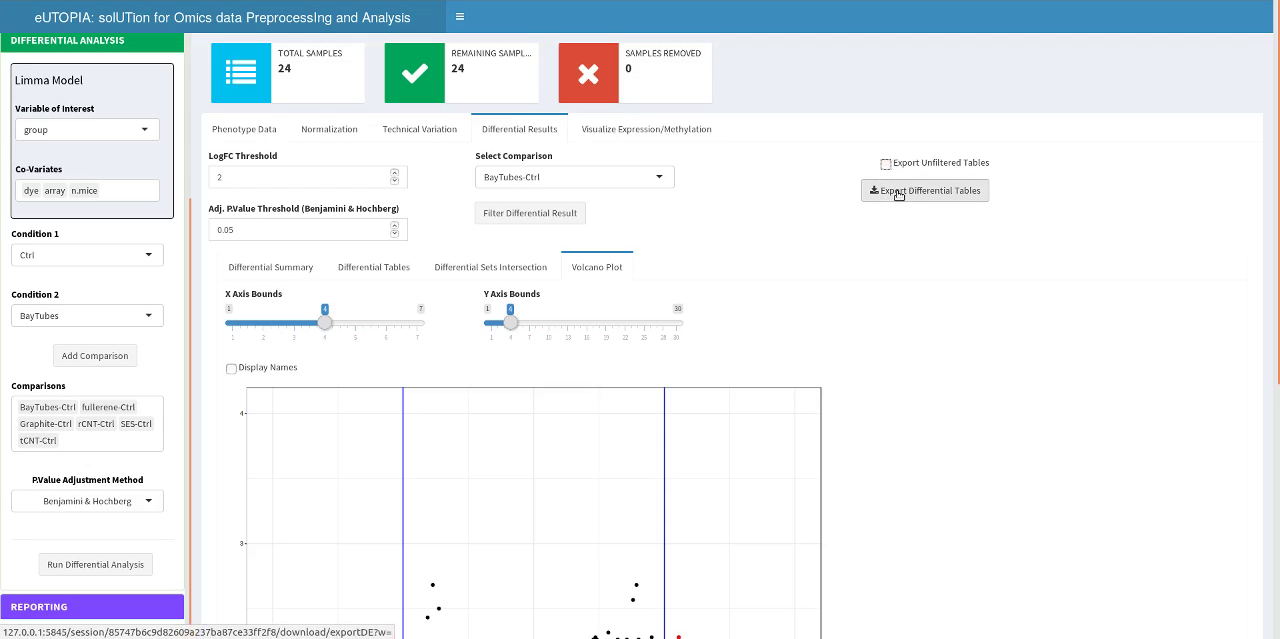** | **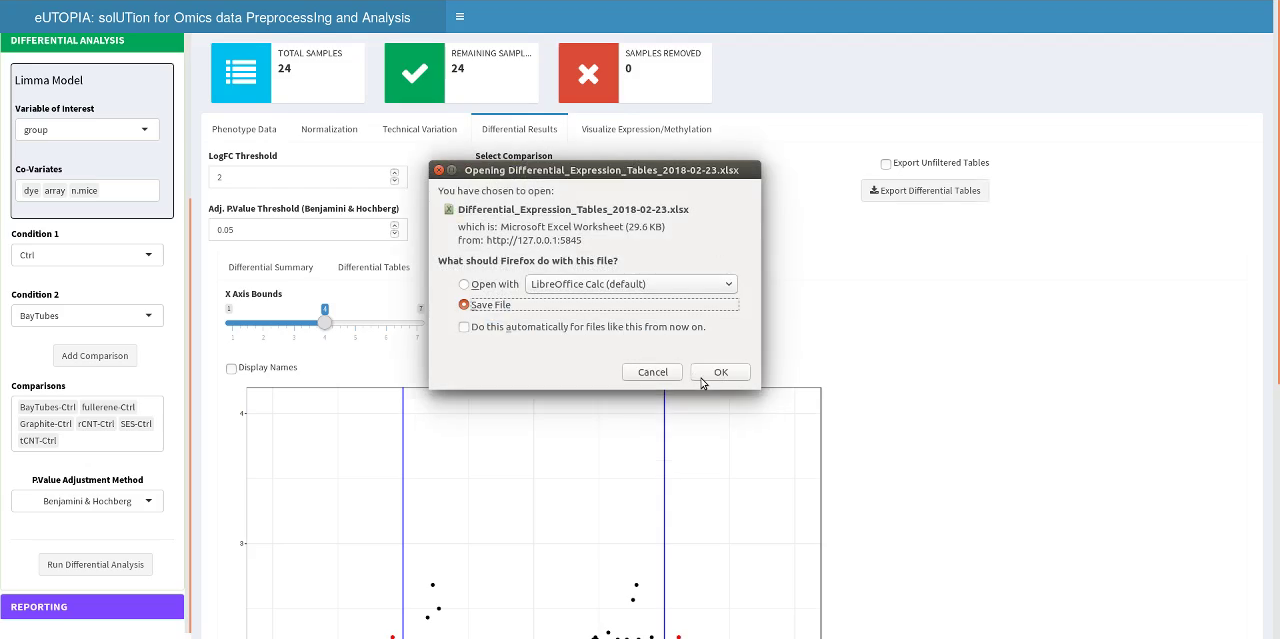** |

## Exported XLS

**
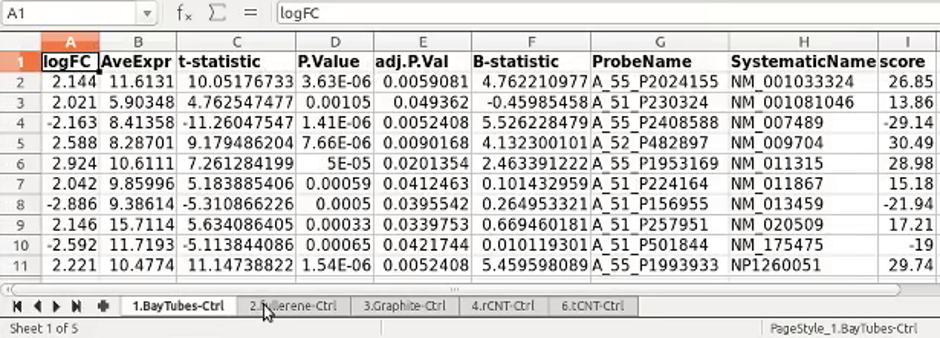
**

# Intersection Plot

*Differential Sets Intersection* sub-tab displays the intersection between the set of differential features between chosen comparisons specified in *Sets to Intersect* multi-select input box. The intersections are represented in two separate forms which are chosen based on the number of chosen sets from intersection.

## UpSet Plot (>4 sets)

For greater than 4 feature sets intersections are represented as an UpSet plot. The intersections are plotted as a bar plot with vertical bars representing the distinct intersections and the intersection size is represented on the y-axis. The feature sets are plotted below as horizontal bars and set size is represented on the corresponding x-axis. The sets to intersection correlation is reported as dot plot where the sets participating in the intersection are highlighted as dark dots and are connected by a thin line.

**
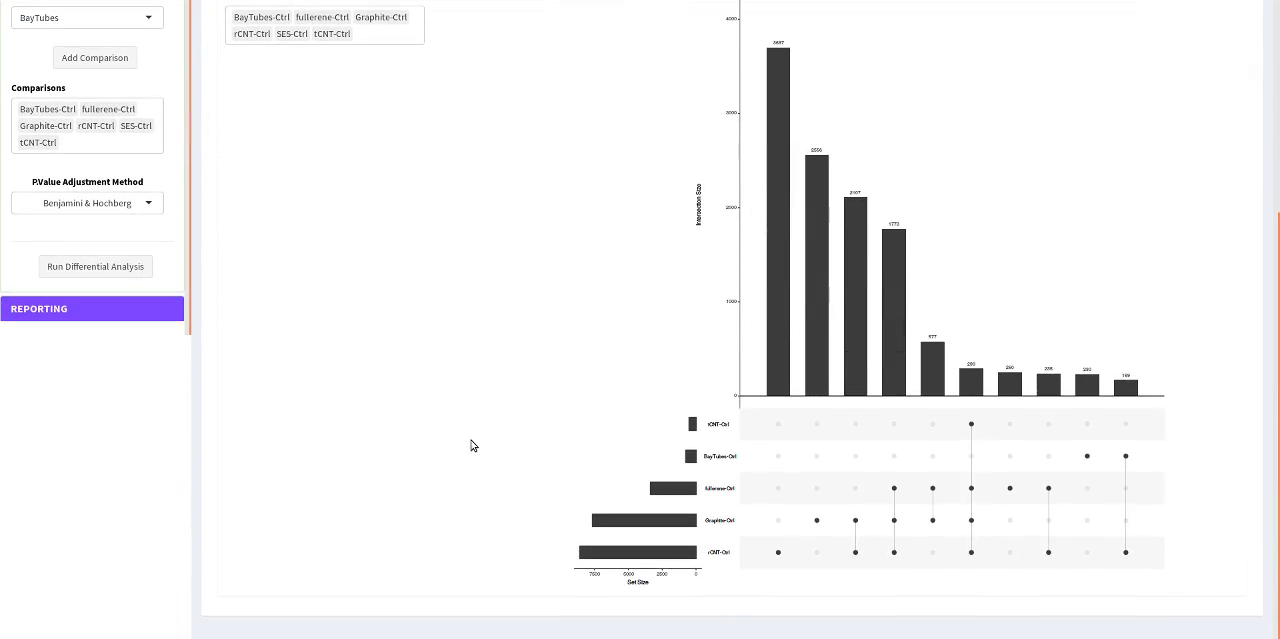
**

## Venn Diagram (<=4 sets)

For 4 or lesser number of feature sets the intersections are represented as a Venn diagram. The sets are plotted as elliptical circles which overlap to form closed curves that represent the logical relationship between the sets. The numbers in each closed curve represent the number of features present in all sets that participate in the formation of that closed curve.

**
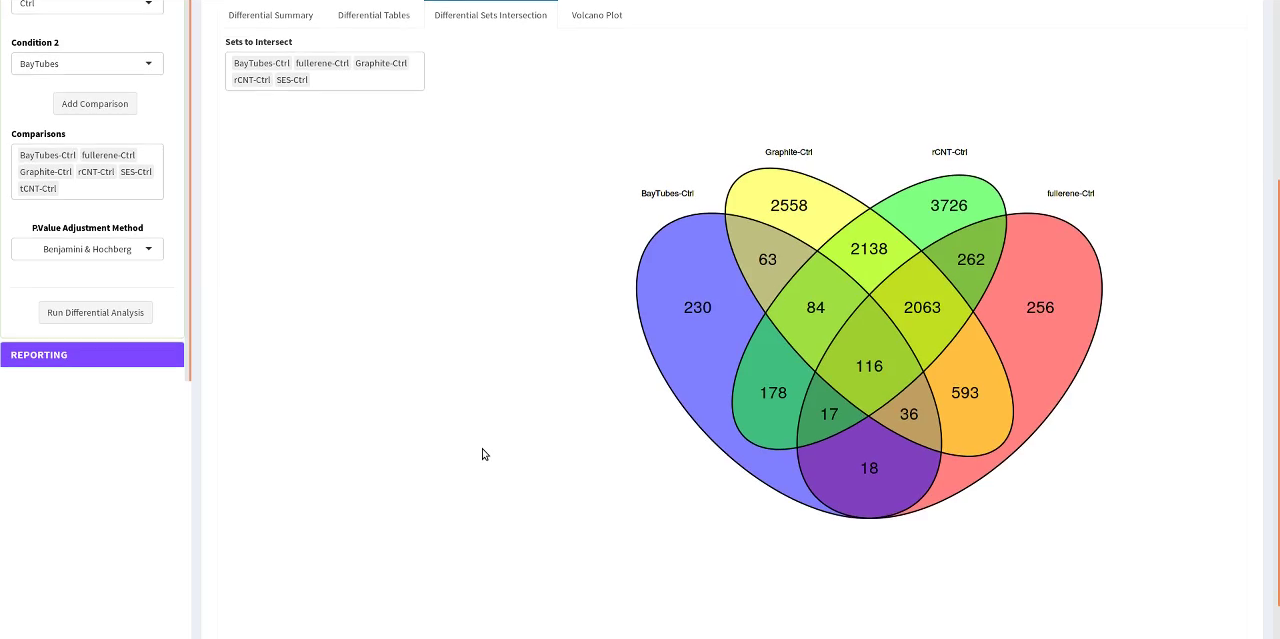
**

# Volcano Plot

*Volcano Plot* sub-tab displays the volcano plot representation of features with logFC on the x-axis and -log10(P.Value) on the y-axis. LogFC threshold is represented by two vertical blue lines, P.Value threshold is represented by the horizontal red line. Features outside of these threshold lines are represented by red colored dots; these red highlighted features are differential features passing the logFC and P.Value thresholds.

**
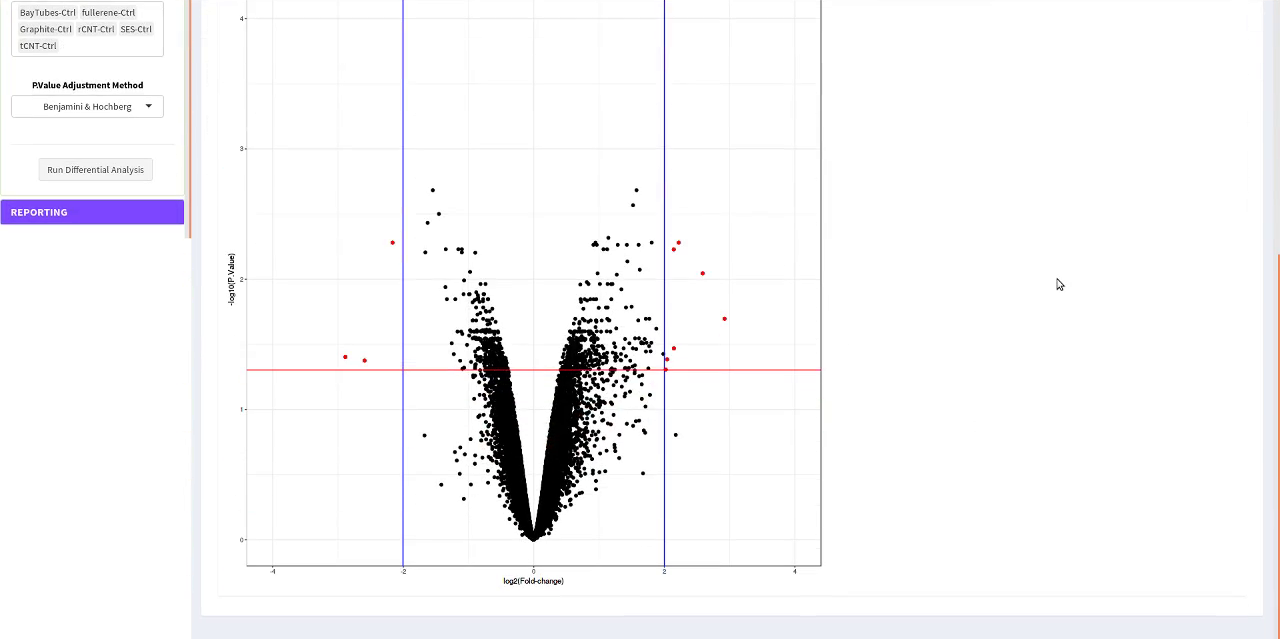
**

## Volcano Plot Controls

The user can customize the display boundaries by adjusting *X Axis Bounds* and *Y Axis Bounds* parameters that control the extent of the x-axis and y-axis to be displayed.

**
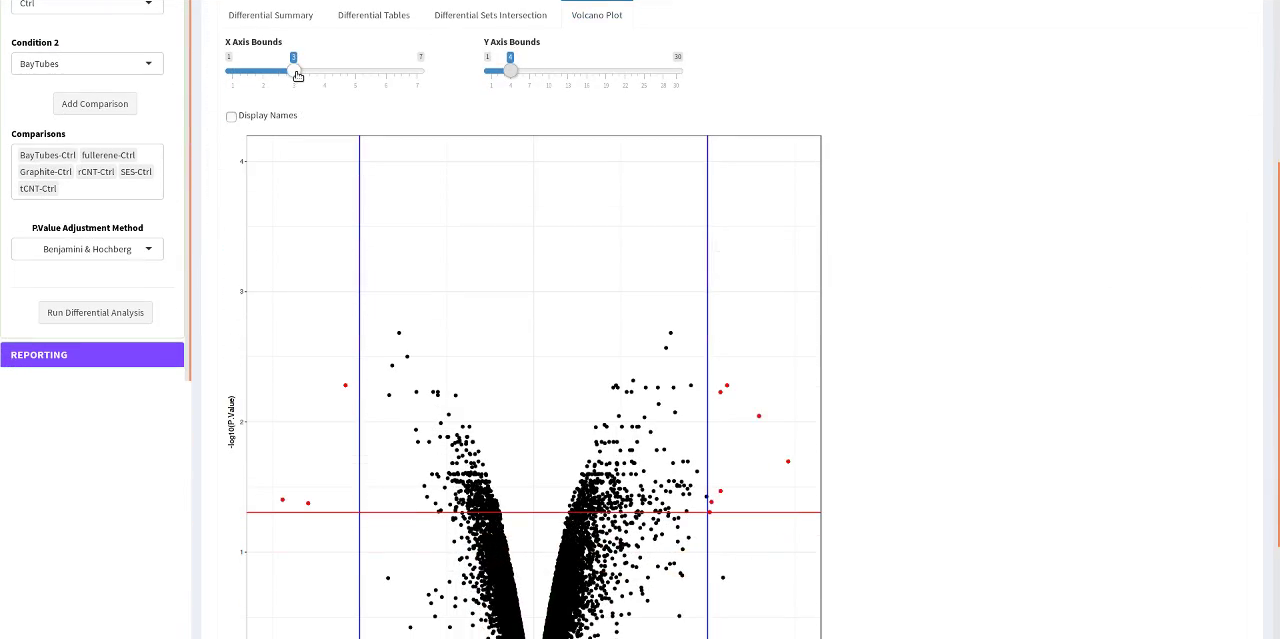
**

## Volcano Plot Feature Names

The user can choose to display the names of the features outside the threshold lines. This is helpful when the filtered features are few in numbers and can be visually inspected.

**
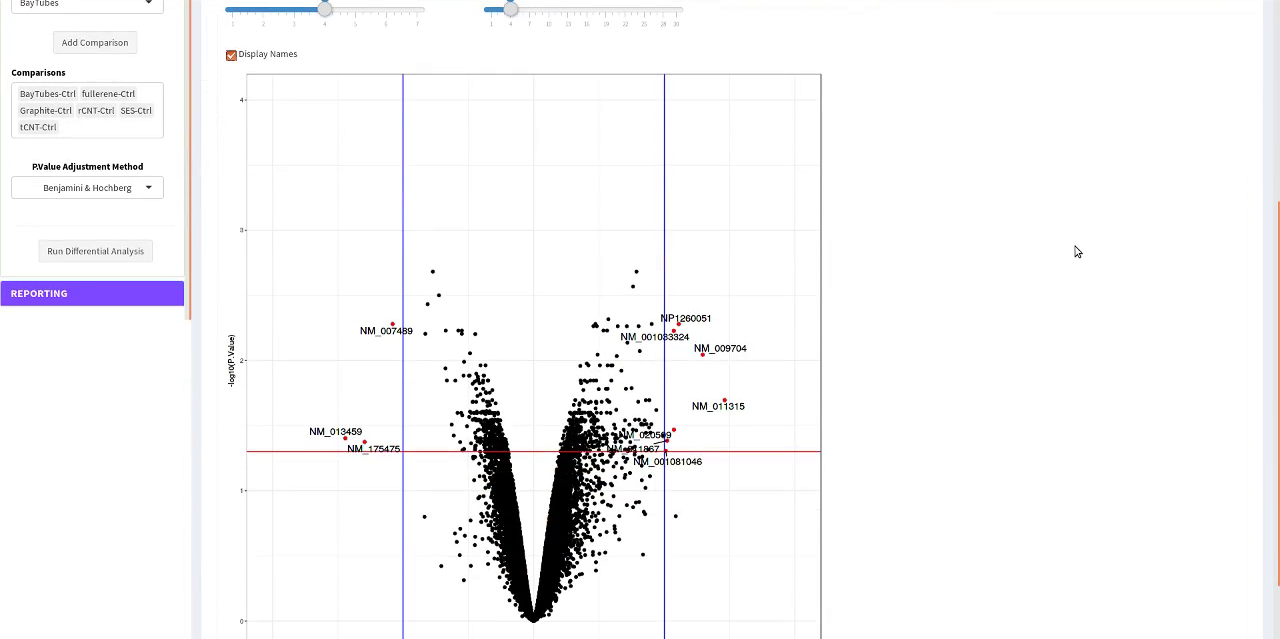
**

# Visualize Expression/Methylation

## Box Plot

Expression/Methylation values can be viewed as box plot from the Box Plot sub-tab nested in the Visualize Expression/Methylation main tab. The latest adjusted data values are used to create the box plots. The user can setup box plot by using three parameters.

**
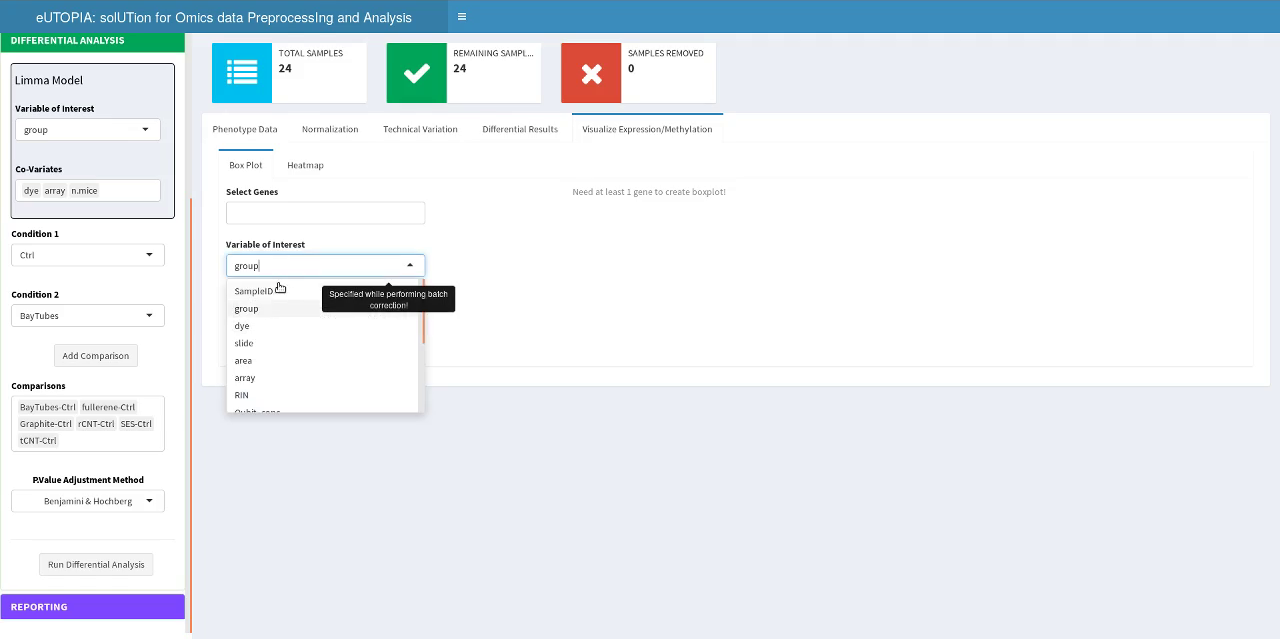
**

## Box Plot Gene Selection

The user can specify the *Variable of Interest* which populates the distinct components from the specified variable in the *Select Conditions* multi-select input box. Chosen conditions from *Select Conditions* in combination with chosen features from *Select Genes* specify the distribution of expression values associated with a feature in selected conditions as box plot.

**
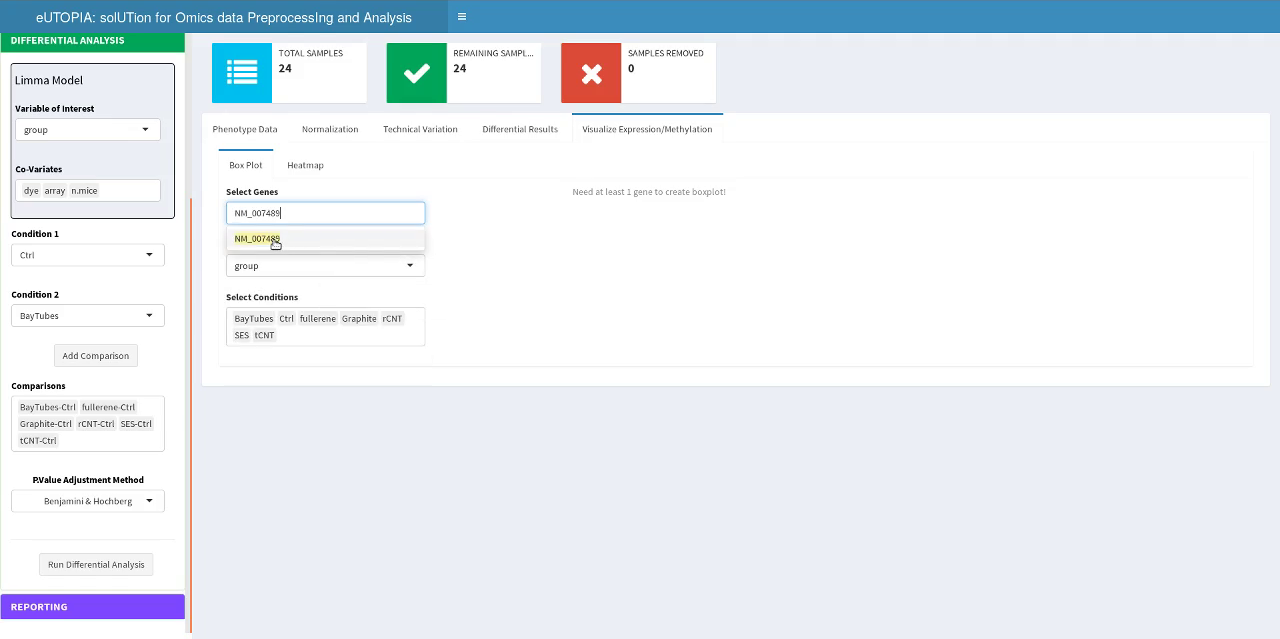
**

## Box Plot Gene Lookup

The *Select Genes* multi-select input box supports auto-lookup of features by typing the prefix of the feature name to assist in search and selection of features from the complete list of feature. One boxplot per condition per feature is plotted with a distinct color.

**
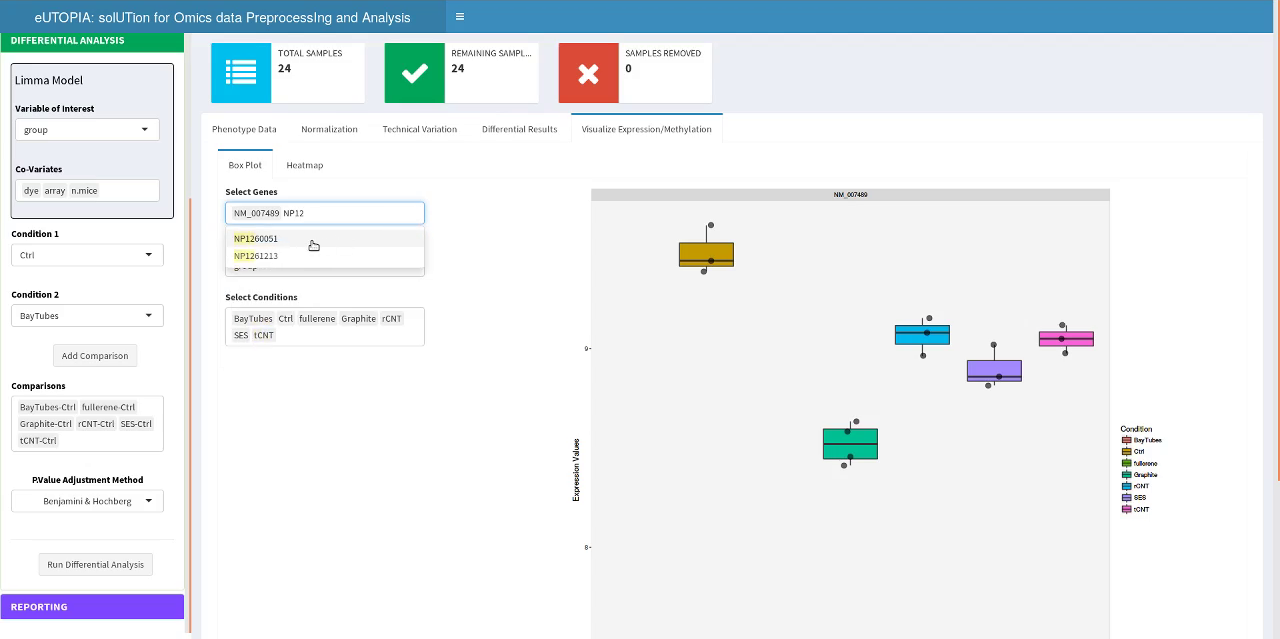
**

## Box Plot (One Gene)

This box plot can be generated for a single feature.

**
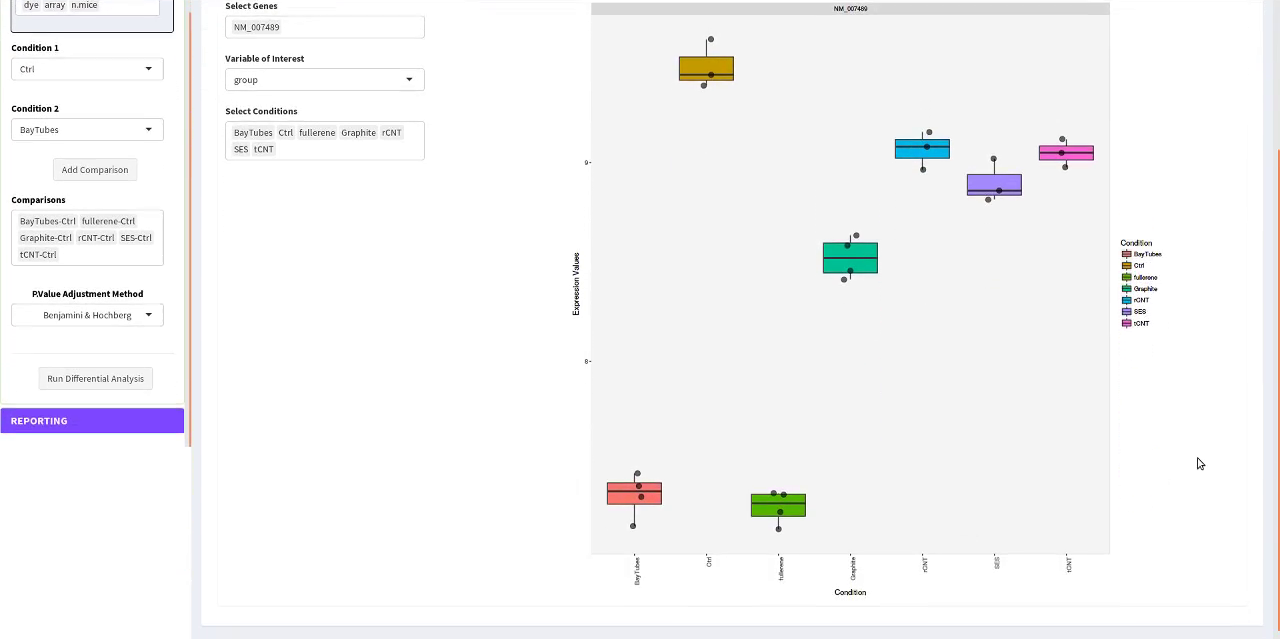
**

## Box Plot (Two Genes)

Multiple feature are represented with a plot per feature adjacent to each other.

**
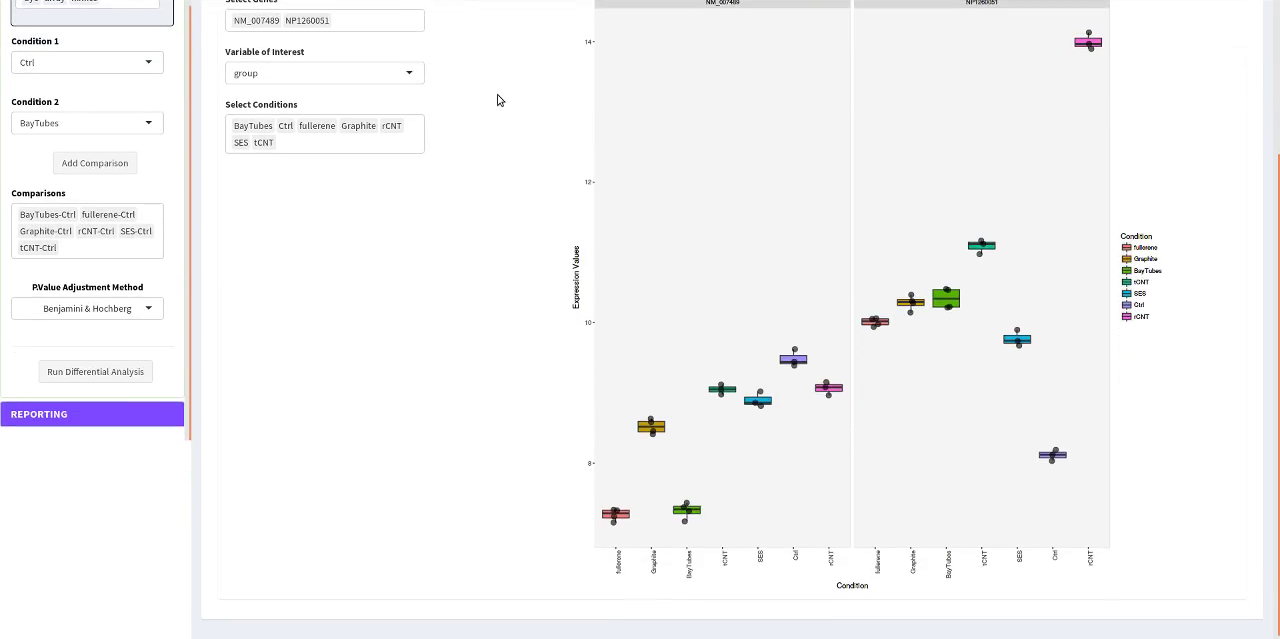
**

## Box Plot (Conditions)

Specific conditions can be chosen to get meaningful representation of the expression value distribution.

**
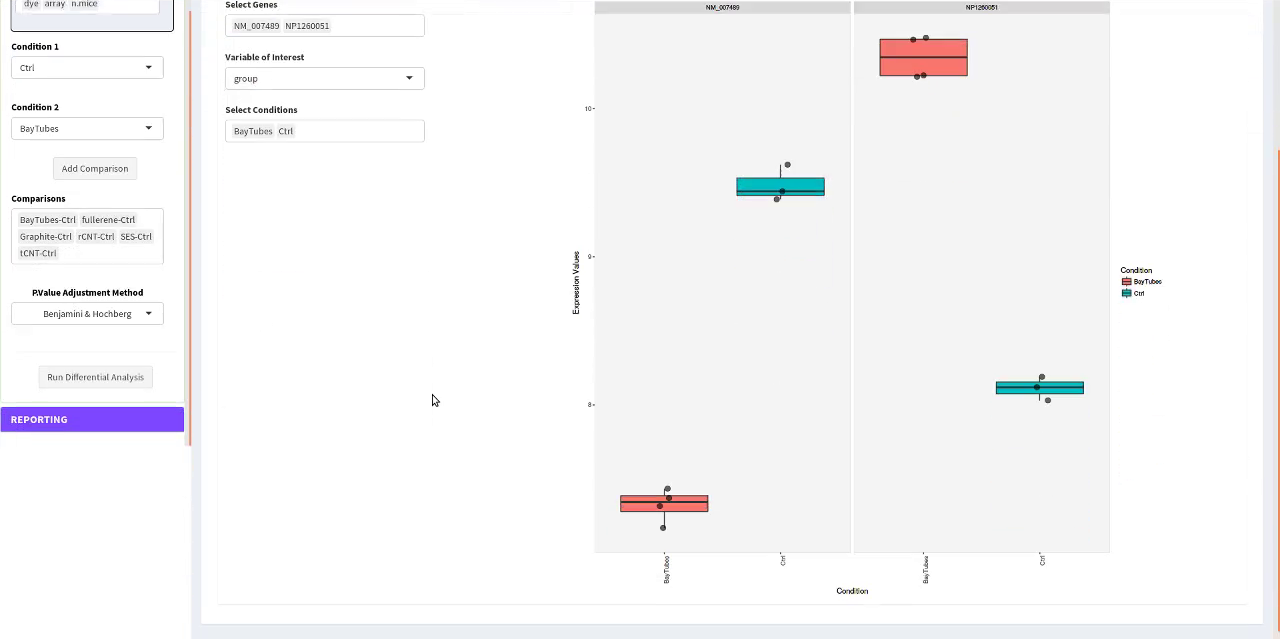
**

## Box Plot (Dye)

This boxplot can be used to explore values distribution in different phenotypic variables for inspection and confirmation.

**
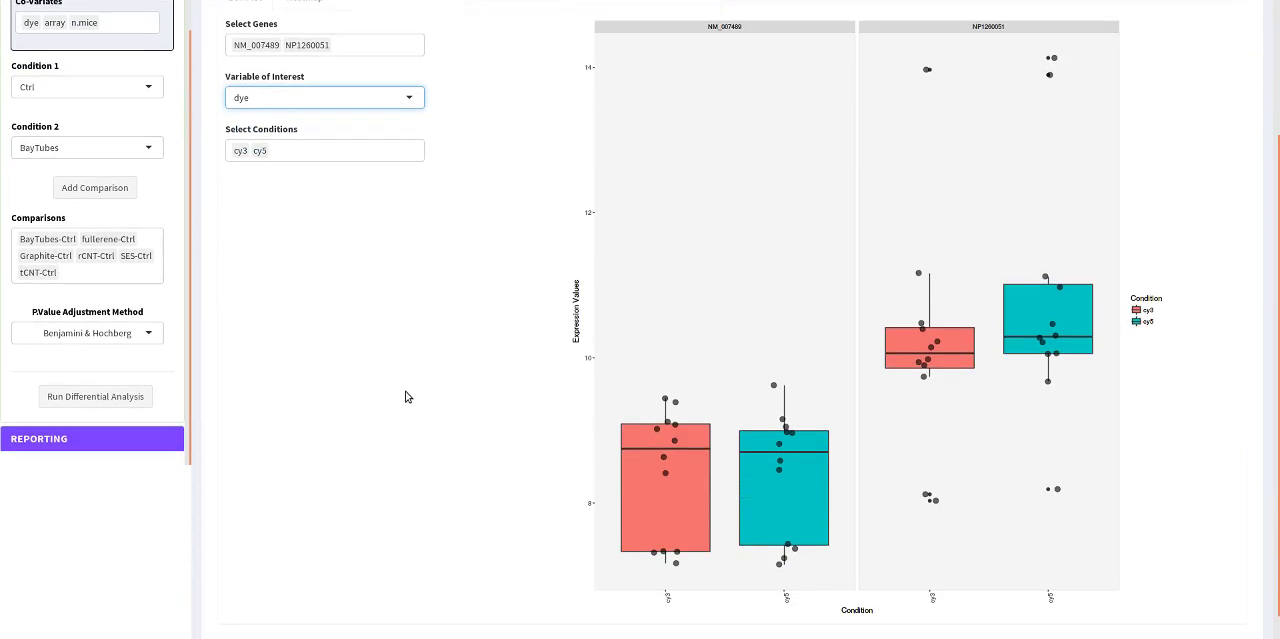
**

## Box Plot (Array)

**
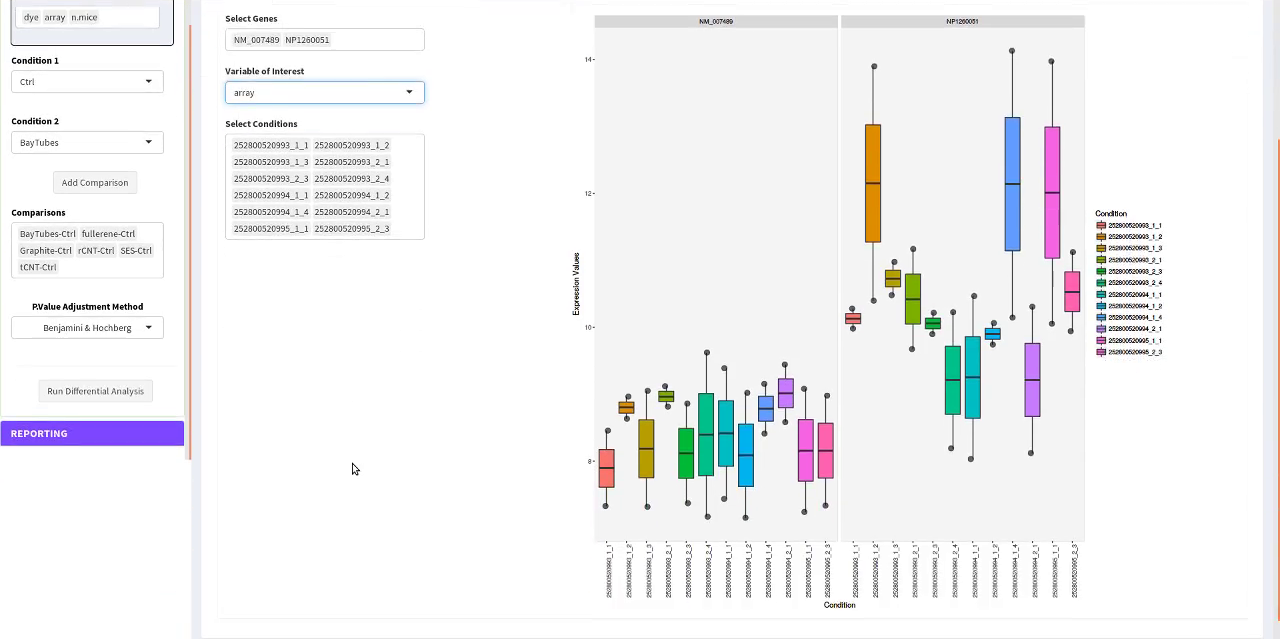
**

## Box Plot (Area)

**
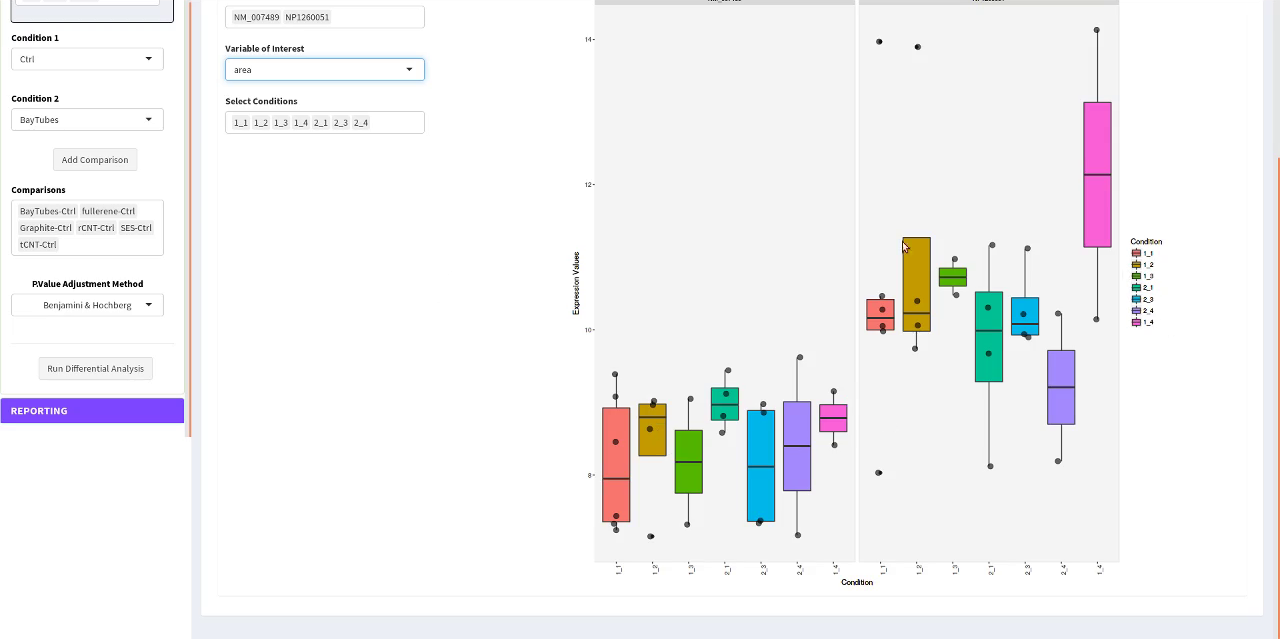
**

## Heatmap

Heatmap of differential features can be viewed from the *Heatmap* sub-tab nested in the *Visualize Expression/Methylation* main tab. The user can setup the heatmap representation by adjusting *Percentage of Highly Differential Genes*, *For Comparison(s)*, and *Select Conditions* parameters. Differential analysis contrasts are specified in *For Comparison(s)* and the conditions from the variable of interest are provided in *Select Conditions*.

**
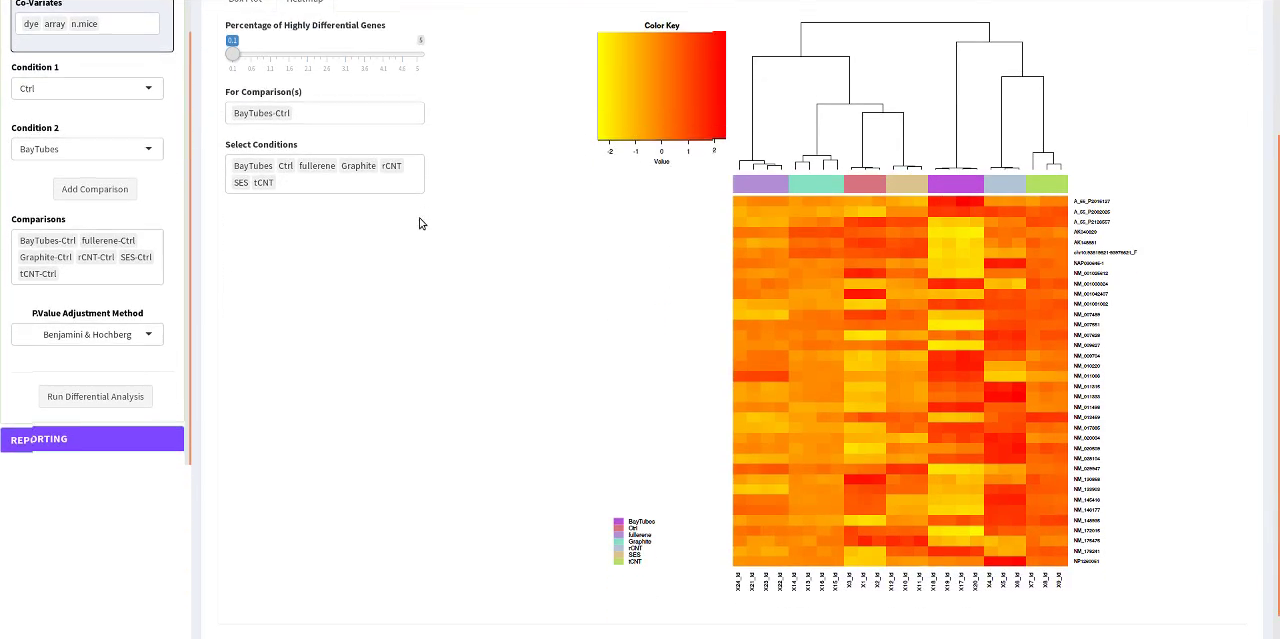
**

## Heatmap (Two Comparisons)

Differential features from multiple comparisons can be displayed together in a single heatmap.

**
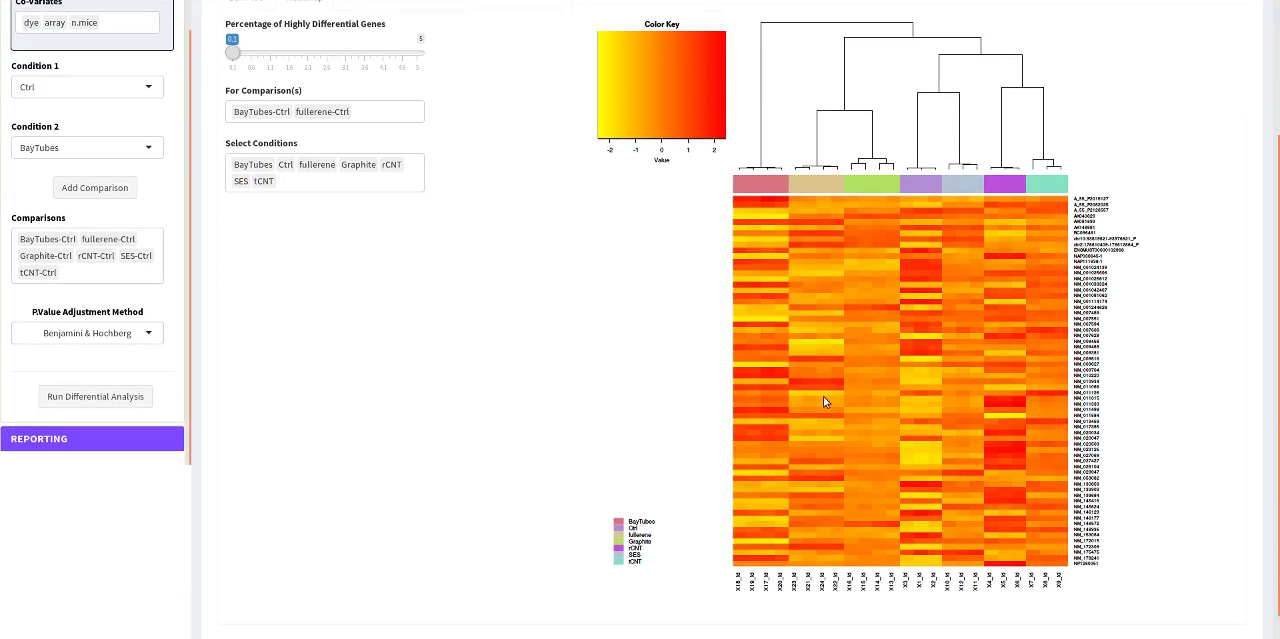
**

## Heatmap (Specific Conditions)

Customizing the set of conditions to match the comparisons creates more readable plots. Samples on the x-axis correspond to the chosen conditions.

**
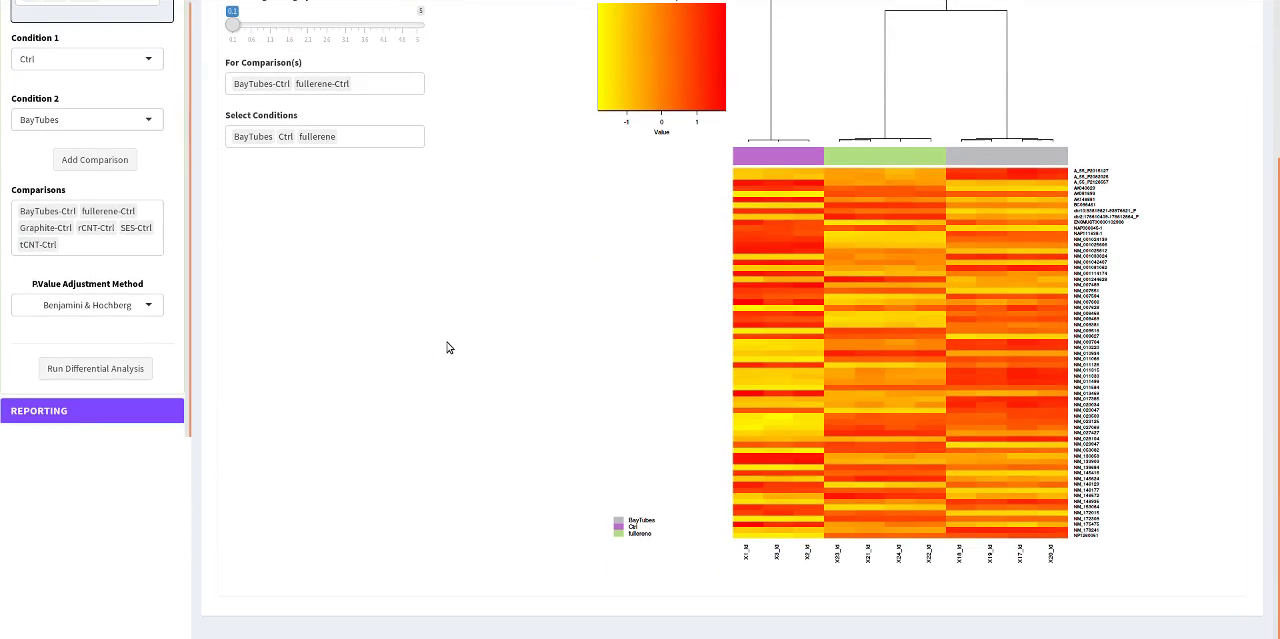
**

## Heatmap (High Percentage)

Adjusting the percentage of differential features accordingly plots the features on the y-axis.

**
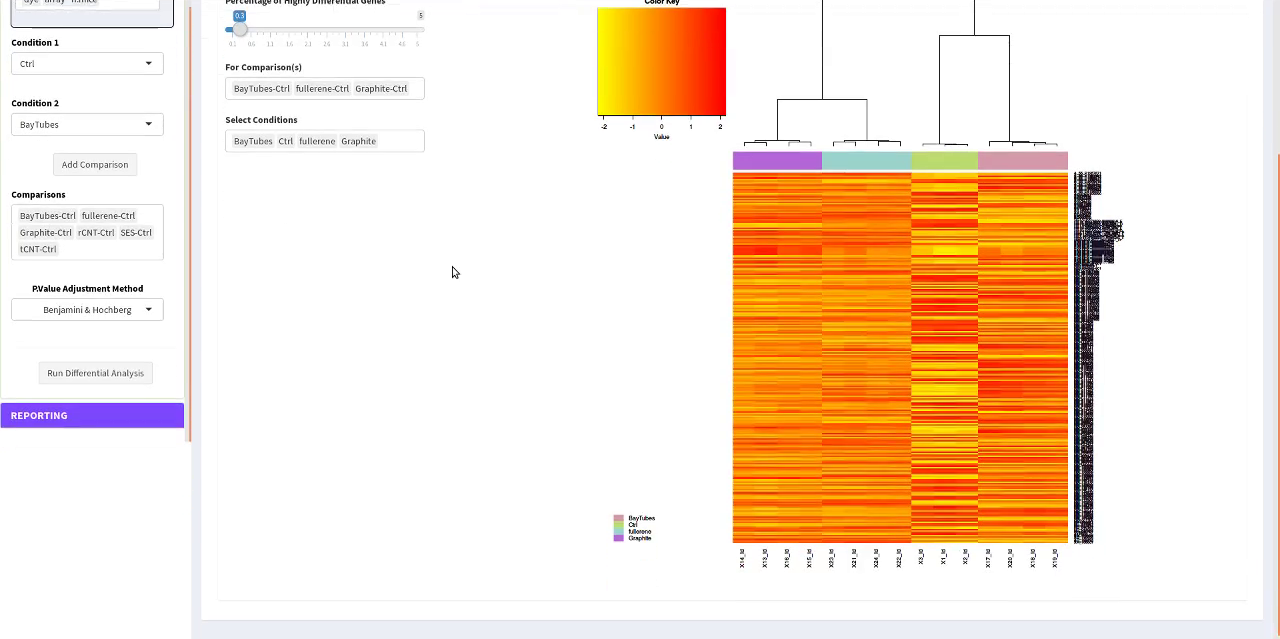
**

# Reporting

REPORTING tab in the sidebar contains buttons to export expression matrices with different levels of processing and button to generate Analysis Report. Analysis report will contain plot representations as configured in the current analysis.

| Available Options | Export Analysis Report |
| --- | --- |
| **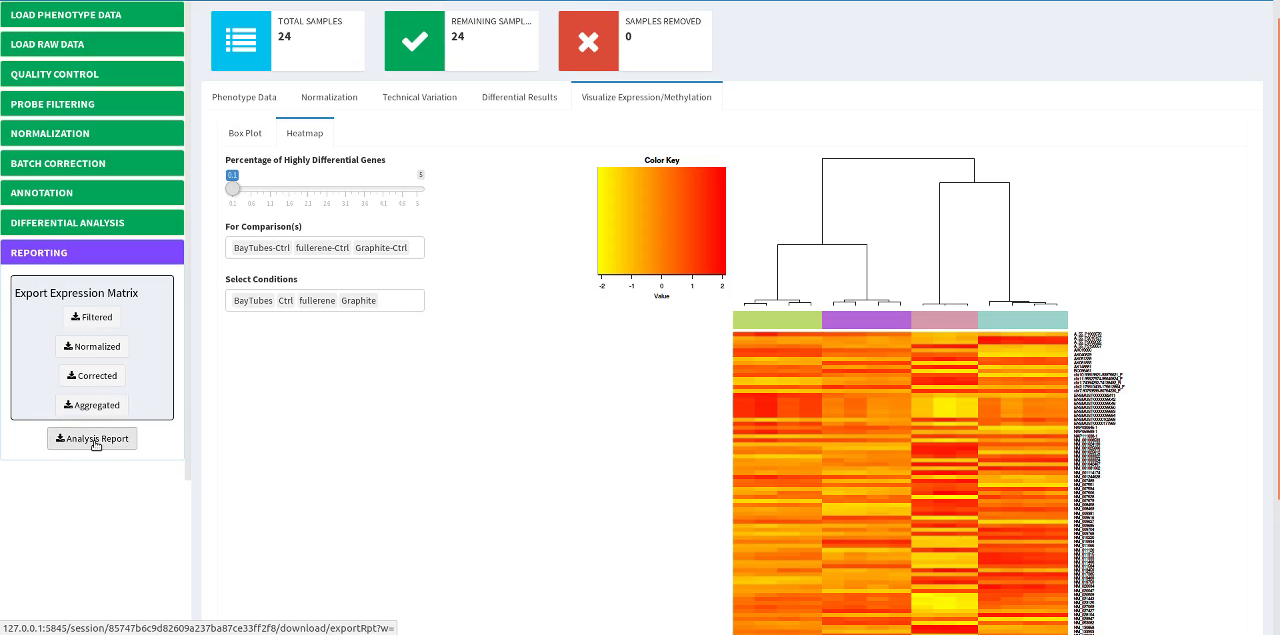** | **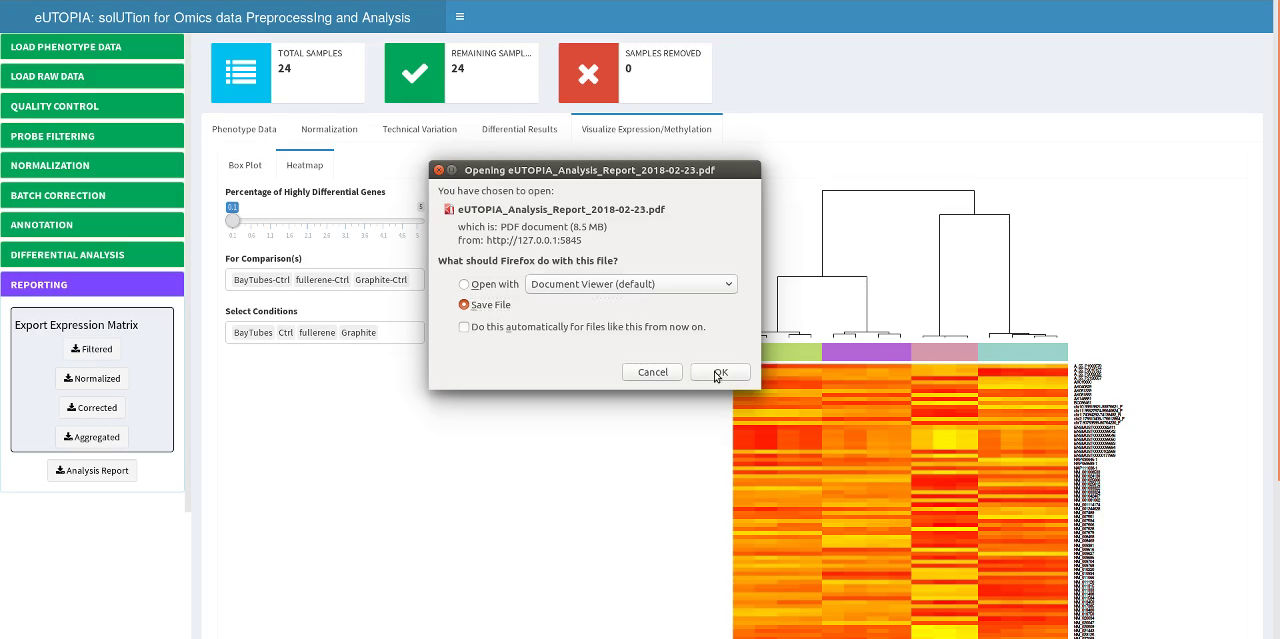** |

## Exporting Analysis Report

**
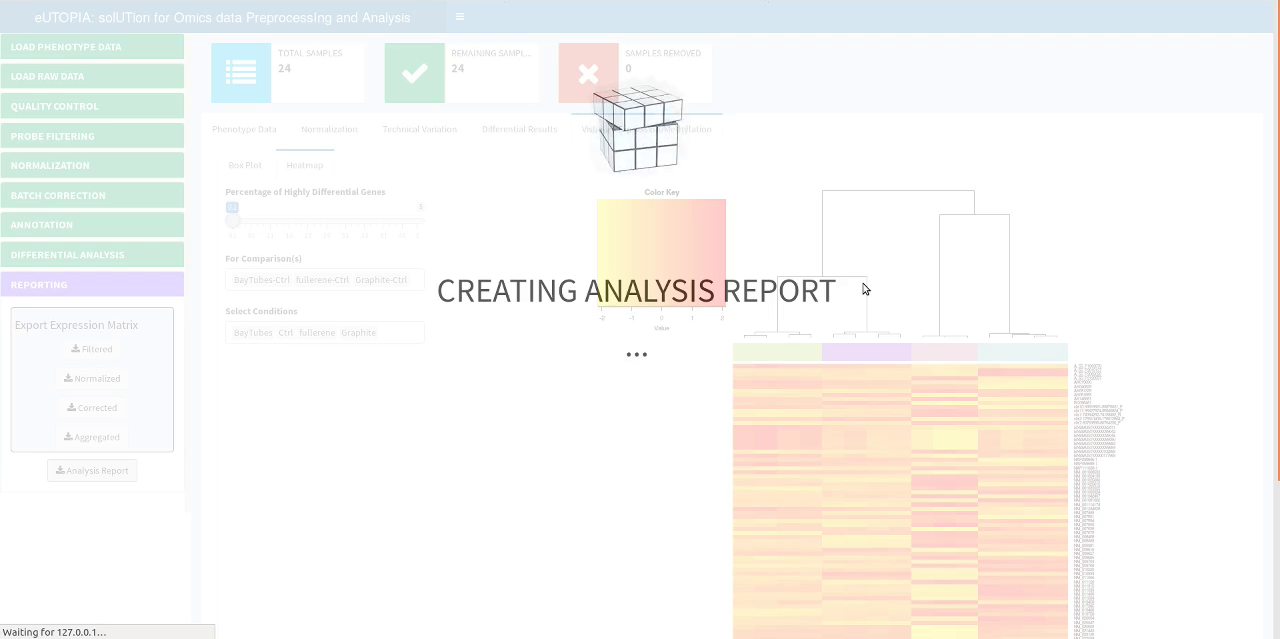
**

## Analysis Report Index

## Analysis Report Content

# Terminate eUTOPIA Session

Finally stop the R session to close eUTOPIA instance.

# eUTOPIA dependencies

| Name | Function | Citation |
| --- | --- | --- |
| R Shiny | Graphical User Interface | (Chang *et al.*, 2017) |
| shinyjs | Dynamic UI features | (Attali, 2018) |
| shinyBS | Dynamic UI features | (Bailey, 2015) |
| shinydashboard | Graphical User Interface | (Chang and Ribeiro, 2018) |
| shinyFiles | Directory Browser UI Component | (Pedersen, 2016) |
| shinycssloader | Plot loading graphics | (Sali, 2017) |
| DT | Tables UI component | (Xie, 2018) |
| rhandsontable | Interactive and formatted table UI component | (Owen, 2018) |
| ggplot2 | Volcano plot representation | (Wickham, 2009) |
| ggrepel | Volcano plot representation | (Slowikowski, 2017) |
| gplots | Heatmap representation | (Warnes *et al.*, 2016) |
| VennDiagram | Venn representation | (Chen, 2018) |
| RColorBrewer | Color gradient | (Neuwirth, 2014) |
| randomcoloR | Distinct unique colors | (Ammar, 2017) |
| WriteXLS | Export of tabular data | (Schwartz *et al.*, 2015) |
| rmarkdown | PDF report creation | (Allaire *et al.*, 2018) |
| reshape2 | Tabular manipulation | (Wickham, 2007) |
| infotheo | Discretize data | (Meyer, 2014) |
| swamp | Adjustment and visualization of expression data | (Lauss, 2017) |
| UpSetR | Set intersection representation as UpSet plot | (Conway *et al.*, 2017) |
| devtools | Installation of Affymetrix CDF annotation from source | (Wickham *et al.*, 2018) |
| limma | Expression normalization, differential analysis, and visualization | (Ritchie *et al.*, 2015) |
| sva | Surrogate variable identification and expression data correction | (Leek *et al.*, 2012) |
| affy | Affymetrix raw data processing and normalization | (Gautier *et al.*, 2004) |
| affyio | Information from Affymetrix CEL files | (Bolstad, 2017) |
| simpleaffy | CDF annotation compatibility with QC | (Miller, 2017) |
| affyQCReport | Quality control of Affymetrix raw data | (Parman *et al.*, 2017) |
| arrayQualityMetrics | Quality control of Agilent raw data | (Kauffmann *et al.*, 2009) |
| yaqcaffy | Quality Control of Affymetrix raw data | (Gatto, 2017) |
| made4 | Heatmap representation | (Culhane *et al.*, 2005) |
| minfi | Illumina methylation raw data processing, normalization, filtering, and visualization | (Aryee *et al.*, 2014) |
| IlluminaHumanMethylation450kmanifest | Manifest for Illumina 's 450k methylation arrays | (Hansen,K.D. and Aryee,M., 2012) |
| IlluminaHumanMethylation450kanno.ilmn12.hg19 | Annotation for Illumina's 450k methylation arrays | (Hansen, 2016a) |
| IlluminaHumanMethylationEPICmanifest | Manifest for Illumina's EPIC methylation arrays | (Hansen, 2016b) |
| IlluminaHumanMethylationEPICanno.ilm10b2.hg19 | Annotation for Illumina's EPIC methylation arrays | (Hansen, 2016c) |
| shinyMethyl | Illumina methylation array qc report | (Fortin *et al.*, 2014) |
| GO.db | Gene Ontology annotation | (Carlson, 2017) |
| GOSemSim | Gene Ontology semantic similarity for summarization | (Yu *et al.*, 2010) |

# References

Allaire,J.J. *et al.* (2018) rmarkdown: Dynamic Documents for R. *R package version 1.9*.

Ammar,R. (2017) randomcoloR: Generate Attractive Random Colors. *R package version 1.1.0*.

Aryee,M.J. *et al.* (2014) Minfi: a flexible and comprehensive Bioconductor package for the analysis of Infinium DNA methylation microarrays. *Bioinformatics*, **30**, 1363–1369.

Attali,D. (2018) shinyjs: Easily Improve the User Experience of Your Shiny Apps in Seconds. *R package version 1.0*.

Bailey,E. (2015) shinyBS: Twitter Bootstrap Components for Shiny. *R package version 0.61*.

Barrett,T. *et al.* (2013) NCBI GEO: archive for functional genomics data sets—update. *Nucleic Acids Res*, **41**, D991–D995.

Bolstad,B. (2017) affyio: Tools for parsing Affymetrix data files. *R package version 1.48.0*.

Carlson,M. (2017) GO.db: A set of annotation maps describing the entire Gene Ontology. *R package version 3.5.0*.

Chang,W. *et al.* (2017) shiny: Web Application Framework for R. *R package version 1.0.5*.

Chang,W. and Ribeiro,B.B. (2018) shinydashboard: Create Dashboards with 'Shiny'. *R package version 0.7.0*.

Chen,H. (2018) VennDiagram: Generate High-Resolution Venn and Euler Plots. *R package version 1.6.20*.

Conway,J.R. *et al.* (2017) UpSetR: an R package for the visualization of intersecting sets and their properties. *Bioinformatics*, **33**, 2938–2940.

Culhane,A.C. *et al.* (2005) MADE4: an R package for multivariate analysis of gene expression data. *Bioinformatics*, **21**, 2789–2790.

Fortin,J.-P. *et al.* (2014) shinyMethyl: interactive quality control of Illumina 450k DNA methylation arrays in R. *F1000Res*, **3**.

Gatto,L. (2017) yaqcaffy: Affymetrix expression data quality control and reproducibility analysis. *R package version 1.38.0*.

Gautier,L. *et al.* (2004) affy—analysis of Affymetrix GeneChip data at the probe level. *Bioinformatics*, **20**, 307–315.

Hansen,K.D. and Aryee,M. (2012) IlluminaHumanMethylation450kmanifest: Annotation for Illumina's 450k methylation arrays. *R package version 0.4.0*.

Hansen,K.D. (2016a) IlluminaHumanMethylation450kanno.ilmn12.hg19: Annotation for Illumina's 450k methylation arrays. *R package version 0.6.0*.

Hansen,K.D. (2016b). IlluminaHumanMethylationEPICmanifest: Manifest for Illumina's EPIC methylation arrays. *R package version 0.3.0*.

Hansen,K.D. (2016c) IlluminaHumanMethylationEPICanno.ilm10b2.hg19: Annotation for Illumina's EPIC methylation arrays. *R package version 0.6.0*.

Kauffmann,A. *et al.* (2009) arrayQualityMetrics—a bioconductor package for quality assessment of microarray data. *Bioinformatics*, **25**, 415–416.

Kinaret,P. *et al.* (2017) Network Analysis Reveals Similar Transcriptomic Responses to Intrinsic Properties of Carbon Nanomaterials in Vitro and in Vivo. *ACS Nano*, **11**, 3786–3796.

Lauss,M. (2017) swamp: Visualization, Analysis and Adjustment of High-Dimensional Data in Respect to Sample Annotations. *R package version 1.3.1*.

Leek,J.T. *et al.* (2012) The sva package for removing batch effects and other unwanted variation in high-throughput experiments. *Bioinformatics*, **28**, 882–883.

Meyer,P.E. (2014) infotheo: Information-Theoretic Measures. *R package version 1.2.0*.

Miller,C.J. (2017) simpleaffy: Very simple high level analysis of Affymetrix data.

Neuwirth,E. (2014) RColorBrewer: ColorBrewer Palettes. *R package version 1.1-2*.

Owen,J. (2018). rhandsontable: Interface to the 'Handsontable.js' Library. *R package version 0.3.6*.

Parman,C. *et al.* (2017) affyQCReport: QC Report Generation for affyBatch objects. *R package version 1.56.0*.

Pedersen,T.L. (2016) shinyFiles: A Server-Side File System Viewer for Shiny. *R package version 0.6.2*.

Ritchie,M.E. *et al.* (2015) limma powers differential expression analyses for RNA-sequencing and microarray studies. *Nucleic Acids Res*, **43**, e47.

Sali,A. (2017) shinycssloaders: Add CSS Loading Animations to 'shiny' Outputs. *R package version 0.2.0*.

Schwartz,M. *et al.* (2015) WriteXLS: Cross-Platform Perl Based R Function to Create Excel 2003 (XLS) and Excel 2007 (XLSX) Files. *R package version 4.0.0*.

Slowikowski,K. (2017) ggrepel: Repulsive Text and Label Geoms for 'ggplot2'. *R package version 0.7.0*.

Yu,G. *et al.* (2010) GOSemSim: an R package for measuring semantic similarity among GO terms and gene products. *Bioinformatics*, **26**, 976–978.

Warnes,G.R. *et al.* (2016) gplots: Various R Programming Tools for Plotting Data. *R package version 3.0.1*.

Wickham,H. (2007) Reshaping Data with the reshape Package. *Journal of Statistical Software,* **21**, 1-20.

Wickham,H. (2009) *ggplot2: Elegant Graphics for Data Analysis*. New York: Springer-Verlag.

Wickham,H. *et al.* (2018) devtools: Tools to Make Developing R Packages Easier. *R package version 1.13.5*.

Xie,Y. (2018) DT: A Wrapper of the JavaScript Library 'DataTables'. *R package version 0.4*.
